# Will the Mediterranean Sea Be a Cul‐de‐Sac for Marine Gastropods Under Climate Change?

**DOI:** 10.1002/ece3.73677

**Published:** 2026-06-09

**Authors:** Arianna Giannini, Chiara Mancino, Luigi Maiorano, Marco Oliverio

**Affiliations:** ^1^ Department of Biology and Biotechnologies “Charles Darwin” Sapienza University of Rome Rome Italy

**Keywords:** global warming, Mediterranean Sea, Mollusca, multitemporal SDM, range shift, species distribution models

## Abstract

Marine ecosystems are undergoing rapid transformation under climate change, yet the responses of many marine invertebrates remain vastly understudied. In particular, for many benthic gastropods there is a striking imbalance between their traditional appreciation by shell collectors—and, consequently, their consistent representation in Natural History Collections—and the limited attention they receive in ecological and conservation studies. Focusing on the northeastern Atlantic and the Mediterranean, the cowries *Luria lurida*, *Naria spurca*, *Zonaria pyrum* and the frog‐shell *Talisman scrobilator* are emblematic examples of this knowledge gap, despite being frequently mentioned as species of conservation concern. Using long‐term occurrence records spanning more than a century, we modelled past and present distributions of these species and explored their potential responses to future climate scenarios through a multi‐temporal Species Distribution Modelling framework. Our results show that intermediate climatic conditions—both in time (2050–2060 vs. 2090–2100) and scenario intensity (moderate SSP2‐4.5 versus high‐emission SSP5‐8.5)—may represent a critical transition phase, leading to habitat contractions without compensatory gains in newly emerging suitable areas. The Mediterranean Sea is expected to increasingly function as a cul‐de‐sac, with the dominant circulation patterns strongly limiting outward movements towards cooler regions for species relying on planktic larvae for dispersal. Furthermore, incorporating larval sensitivity to reduced pH suggests that large areas of the Atlantic Ocean may actually result unsuitable for larval persistence, substantially reducing the habitat effectively available for completion of the full life cycle; this highlights the need to account for connectivity, life‐history constraints and juvenile‐stage sensitivity when assessing climate‐driven range shifts in shelled organisms with planktic larvae.

## Graphical abstract

Marine benthic gastropods remain understudied despite extensive historical records, limiting our understanding of their vulnerability to climate change. Using multi‐temporal species distribution models, we show that intermediate future climate conditions may trigger major habitat contractions for four key cowrie and frog‐shell species in the NE Atlantic and Mediterranean. Restricted dispersal pathways and larval sensitivity to ocean acidification further constrain their potential range shifts, underscoring the importance of incorporating life‐history and connectivity constraints into conservation assessments.

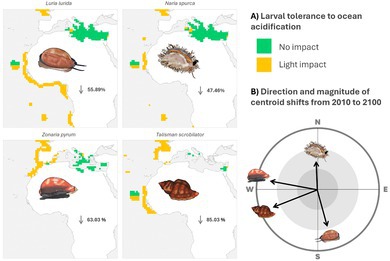

## 1 Introduction

Global environmental conditions are constantly being altered by human activities, which act both directly, by modifying ecosystems (e.g., land use change, deforestation), and indirectly, through pollution (e.g., CO2 emissions, eutrophication) and climate change (e.g., rising temperatures, extreme weather events) (Prakash and Verma 2022). Species and communities are responding to these changes by adapting to new environmental conditions, or changing their distribution to follow the shift of their optimal conditions or, in the extreme case, becoming extinct (Bell and Collins 2008). Therefore, understanding and predicting the responses of species to global changes has a crucial role for biodiversity management and conservation. Species Distribution Models (SDMs) have become invaluable tools for analysing the distribution and bioclimatic niche of species and populations (Franklin 2023). SDMs can also be used to identify the most important predictors that shape the distribution of a species and to forecast its responses to future climate change scenarios (Miller 2010; Franklin 2023). While much progress has been made in studying terrestrial biodiversity via SDM approaches, marine species are much less considered. However, marine species are equally vulnerable to environmental changes (Luypaert et al. 2020), and many have fundamental roles in ecosystem functioning and for the services they provide to people (Barbier 2017).

Gastropods represent an ecologically (Donnarumma et al. 2018) and evolutionarily (Xu et al. 2025) hyper‐diverse group. They provide essential ecosystem services (Rife 2018) and are particularly vulnerable to multiple climate change stressors. These include ocean acidification, which can produce sub‐lethal to lethal effects especially on juveniles (Gazeau et al. 2013; Parker et al. 2013); altered nutrient availability (Moraitis et al. 2018; Reiss et al. 2014); salinisation, which disrupts physiological processes and metabolism (Pourmozaffar et al. 2020; Zettler et al. 2014); and sea warming, which further exacerbates their stress responses (Bosch et al. 2018). However, many (if not most) gastropod species remain poorly studied, and their responses to environmental changes are largely unknown.

**FIGURE 1:**
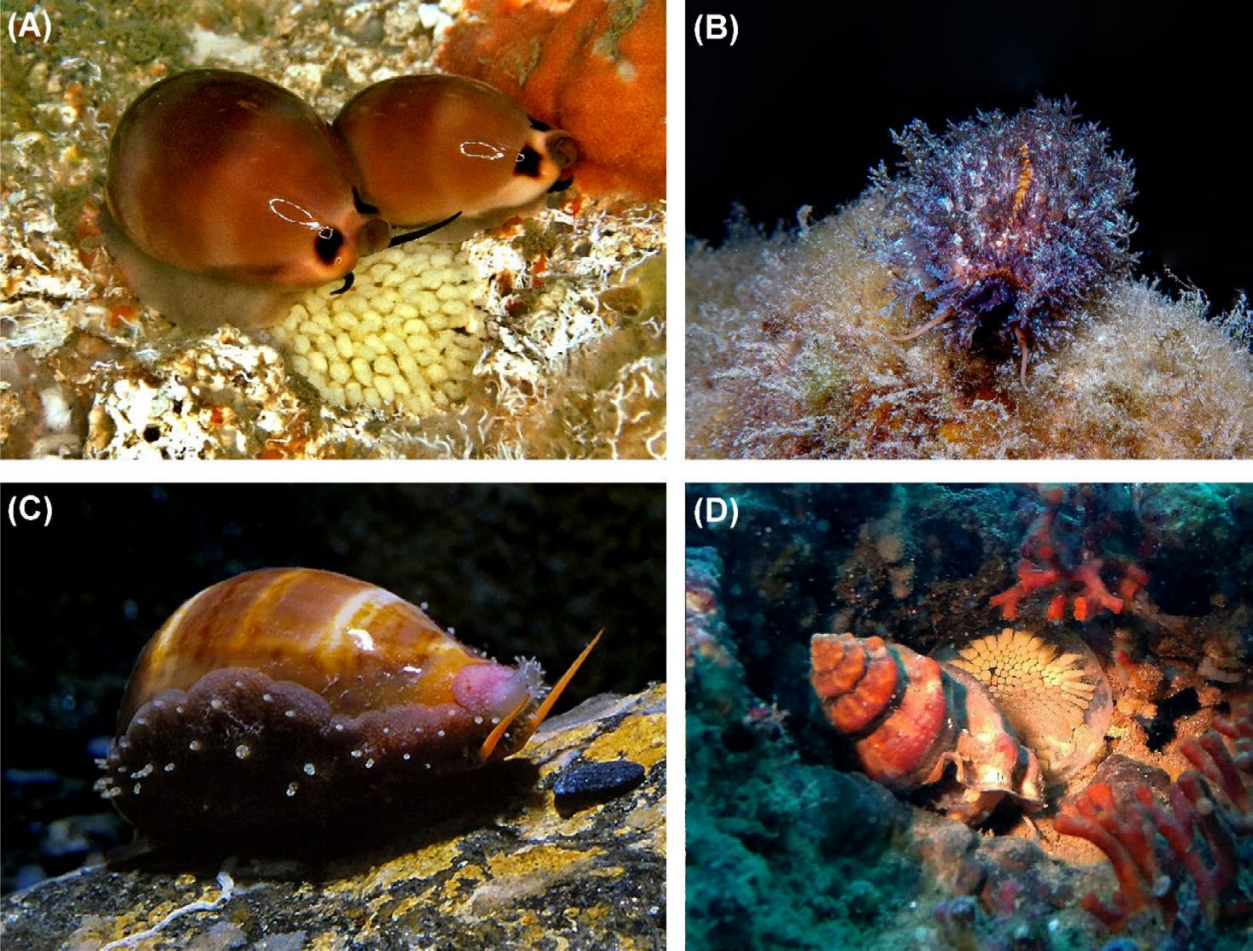
The four gastropod species studied in the present work. (A) Two fallow cowries, *Luria lurida*, female (larger) and male, with egg‐capsules, Almería (southern Spain) [photo by Diego Moreno Lampreave]. (B) The spotted cowrie, *Naria spurca*, on a rocky bottom in Granada (southern Spain) [photo by Luis Sánchez Tocino]. (C) The pear cowry, *Zonaria pyrum*, on a rocky bottom in Granada (southern Spain) [photo by Luis Sánchez Tocino]. (C) The pitted frog snail, *Talisman scrobilator*, a female with the egg‐capsules, Águilas, Murcia (southern Spain) [photo by Brian Cunningham Aparicio].

The Mediterranean cowries (family Cypraeidae) *Luria lurida* (Linnaeus, 1758), *Naria spurca* (Linnaeus, 1758) and *Zonaria pyrum* (Gmelin, 1791), and the frog‐shell *Talisman scrobilator* (Linnaeus, 1758) (family Bursidae) are emblematic examples of this knowledge gap (Figure 1). Despite their long‐standing recognition by shell collectors and, consequently, their consistent representation in both public and private Natural History Collections (NHCs), these iconic species have received limited attention in ecological and conservation studies (Cunningham Aparicio and Verdejo Giurao 2021; Passamonti 2015). To date, none of them has been formally assessed in the IUCN Red List (https://www.iucnredlist.org), and while the three cowries are protected under the Bern (1982, Annex II) and Barcelona (1977, Annex II) Conventions, *Talisman scrobilator*—despite being notably rare—has not been included in any international conservation directive due to insufficient data. At regional scales, however, *Talisman scrobilator* is recognised as threatened, being listed in the Libro Rojo de los Invertebrados de Andalucía (Barea‐Azcón et al. 2008), reported as ‘potentially threatened’ by Scotti and Chemello (2000), and considered a ‘species in decline’ in the western Mediterranean (Verdejo Guirao 2001). This striking imbalance between their cultural prominence and scientific obscurity exemplifies a broader gap in our understanding of marine benthic gastropods—species that are often abundant in NHCs but underrepresented in ecological studies.

These four gastropods share a benthic lifestyle on hard substrata, yet differ in their bathymetric and distribution patterns. *Luria lurida*, *Naria spurca* and *Talisman scrobilator* are mostly associated with shallow subtidal habitats, whereas *Zonaria pyrum* extends its range to the continental shelf and upper slope. Together, they span the Mediterranean Sea and adjacent Atlantic regions, including the main archipelagos and islands. *Luria lurida* and *Naria spurca* range from the Iberian Peninsula along the West African coast to Angola, and occur in the Canary Islands, Azores and Cape Verde (Doneddu 1999; Meyer 2003; Passamonti 2015); *Zonaria pyrum* extends from southern Portugal to the Gulf of Guinea (Doneddu 1999; Meyer 2003; Passamonti 2015); *Talisman scrobilator* reaches as far south as Dakar, Senegal, with the Canary Islands representing an important stronghold, and additional records from the Azores and Cape Verde (Smriglio et al. 2019; Cunningham Aparicio and Verdejo Giurao 2021).

All four species are carnivores, but their trophic niches differ in specialisation. While cowries (*Luria lurida*, *Naria spurca*, *Zonaria pyrum*) feed predominantly on sponges, the diet of the frog‐shell *Talisman scrobilator* is less documented, although three Hawaiian bursids have been observed feeding on polychaetes and sipunculids (Houbrick and Fretter 1969), and one Maldivian species on polychaetes and starfish (Taylor 1978; Kohn 1983). Interestingly, aquarium experiments by Cunningham Aparicio and Verdejo Giurao (2021) suggested a striking prey preference in *Talisman scrobilator*, which consistently targeted the starfish *Marthasterias glacialis* while ignoring other potential prey, even after extended periods of starvation.

In this study, we address the knowledge gap surrounding these species by reconstructing their historical and current distributions and exploring their potential responses to climate change. We model range changes of *Luria lurida*, *Naria spurca*, *Zonaria pyrum* and *Talisman scrobilator* from 1850 to the present, identifying key environmental predictors and projecting future trends through a multi‐temporal SDM. This approach, which explicitly accounts for temporal variability in environmental conditions, allows a more accurate approximation of fundamental niches (Nogués‐Bravo 2009) and captures a wider spectrum of potential responses to climate change than models calibrated on a single time period (Maiorano et al. 2013).

Applications of multi‐temporal SDMs to marine organisms remain scarce, primarily due to the limited availability of long‐term historical data. In this context, NHCs are particularly valuable sources of multi‐temporal species data, since specimens have been accumulated over decades and in some cases over centuries (Lister 2011), since they represent one of the earliest approaches of documenting the spatial distribution of biodiversity. Malacological collections, in particular, have a long‐standing tradition within NHCs, reflecting centuries of scientific and cultural interest in molluscs, and thus constitute an especially rich archive for reconstructing species distributions (Heppell 1995; Oliverio 2003).

By combining long‐term occurrence data with multi‐temporal environmental reconstructions, this study aims to improve our understanding of how the marine benthic gastropods *Luria lurida*, *Naria spurca*, *Zonaria pyrum* and *Talisman scrobilator* respond to changes in environmental conditions over time and highlight the value of historical datasets for multi‐temporal SDM approaches.

## 2 Materials and Methods

### 2.1 Dataset Compilation

The data on species presence were collected from a variety of sources: museum and private NHCs (see Giannini and Oliverio 2022 for data gathering method details), websites, publicly accessible online databases and scientific literature. Although the use of NHC data may be affected by taxonomic uncertainties, particularly for cryptic or morphologically similar marine molluscs (Aubry et al. 2017; Guedes et al. 2025; Troudet et al. 2017), the species selected in this study are widely recognised and easily identified even by amateur collectors, which likely contributes to a high level of identification accuracy. Knowing that strong intraspecific variability can affect responses of taxa to climate change (Jinga et al. 2021; Serra‐Varela et al. 2015), and that the actual status of the recognised subspecies is still debated we only considered records of the nominal subspecies. To clean, georeference and harmonise species occurrences, we followed the pipeline outlined by Giannini et al. (2025). In particular, records were filtered to remove duplicates, occurrences lacking essential information (e.g., missing coordinates or taxonomic identification), and records with high coordinate uncertainty. Additional manual screening excluded occurrences falling outside the study area or corresponding to fossil records, as well as records considered too vague (e.g., broad locality descriptions or identifications above species level) or potentially unreliable (e.g., ambiguous or doubtful identifications and localities). The number of records per species collected from each source is reported in Table S1.

The four taxa we selected for the analyses are mainly distributed in shallow coastal waters, and therefore many occurrences, although georeferenced correctly, fell outside the limits of the environmental raster layers used in the modelling (see below) due to the coarse spatial resolution of the layers. To solve this issue, these occurrences were moved to the nearest cell using the ‘rSDM’ package (Rodriguez‐Sanchez 2024) in R Studio (Posit Team 2024), by setting a maximum displacement equivalent to the length in metres of the side of an environmental layer cell (i.e., 1°). All records without any information on time of collection (e.g., full date or year) were removed. Finally, the resulting dataset was thinned to a maximum of one occurrence of each species per cell/decade. The final number of species occurrences per decade is shown in Table 1.

**TABLE 1:** Final number of occurrences per decade used in the model for each species.

Decade	*L. lurida*	*N. spurca*	*Z. pyrum*	*T. scrobilator*
1850–1860	1	0	0	3
1860–1870	2	0	0	0
1870–1880	4	0	0	4
1880–1890	0	0	1	0
1890–1900	6	0	2	5
1900–1910	16	14	9	6
1910–1920	24	17	11	11
1920–1930	26	18	12	0
1930–1940	31	25	0	14
1940–1950	33	31	13	22
1950–1960	53	47	21	25
1960–1970	84	70	37	29
1970–1980	136	113	69	43
1980–1990	180	153	91	60
1990–2000	224	193	105	77
2000–2010	260	230	122	96
2010–2020	346	316	151	122
**Total**	**1426**	**1227**	**644**	**517**

### 2.2 Environmental Layers Processing

Because the scientific literature on the selected species is scarce, their precise ecological requirements and the influence of environmental variables on their distribution remain poorly understood. Consequently, we focused on predictors known to strongly affect benthic marine gastropods in general. We selected three key variables: seawater acidity, salinity and temperature (Table 2). Ocean acidification is well documented to cause sub‐lethal to lethal effects in molluscs, including reduced growth rates, impaired shell formation and altered metabolic processes (Gazeau et al. 2013; Parker et al. 2013). Salinity plays a central role in osmoregulation and energy allocation, influencing growth, reproduction and survival (Pourmozaffar et al. 2020; Zettler et al. 2014; Schultz et al. 2023). Temperature is widely recognised as one of the primary drivers of marine mollusc distributions, shaping metabolic rates, thermal tolerance and biogeographic patterns (Bosch et al. 2018; Schultz et al. 2023). Despite the obvious importance of bathymetry as a predictor of marine species distribution, this variable was found to be highly correlated with temperature (Pearson's *r* > 0.95; Spearman's ρ > 0.90) and was therefore excluded to avoid multicollinearity and model overfitting (Dormann et al. 2013).

**TABLE 2:** The selected variables and their VIFs.

Variable	Unit	Sources	VIF
Acidity	pH	Past and future data: EC‐Earth3‐CC, CESM2, CMCC‐ESM2, CNRM‐ESM2‐1 (CMIP6); Current data: Surface Ocean Carbon Fields (CMEMS)	1.71
Salinity	Practical Salinity Scale (S)	Past and future data: EC‐Earth3‐CC, CESM2, CMCC‐ESM2, CNRM‐ESM2‐1 (CMIP6); Current data: Global Ocean Physics Reanalysis (CMEMS)	1.01
Temperature	°C	Past and future data: EC‐Earth3‐CC, CESM2, CMCC‐ESM2, CNRM‐ESM2‐1 (CMIP6); Current data: Global Ocean Physics Reanalysis (CMEMS)	1.70

Environmental data were retrieved from two different databases to cover as much as possible of the time range covered by the occurrences: the Coupled Model Intercomparison Project Phase 6 (CMIP6, https://pcmdi.llnl.gov/CMIP6) and the Copernicus Marine Environment Monitoring Service (CMEMS, https://data.marine.copernicus.eu/products) databases. For the 16 decades from 1850 to 2010 (hereinafter referred to as ‘past conditions’), as well as for future scenarios (hereinafter referred to as ‘future conditions’), we used the CMIP6 data. To mitigate biases inherent in any global circulation model (GCM, Phillips et al. 2006; Kwiatkowski et al. 2020), we produced an ensemble model averaging four climate models: EC‐Earth3‐CC (EC‐Earth Consortium 2020), CESM2 (Danabasoglu 2019), CMCC‐ESM2 (Peano et al. 2021) and CNRM‐ESM2‐1 (Voldoire 2019). These models were selected based on data availability for the variables and the period of interest. Past conditions were obtained from models' historical experiments, while for the future conditions we considered two Shared Socioeconomic Pathway scenarios (O'Neill et al. 2014): the ‘Middle of the road’ pathway (SSP2‐4.5) and the ‘Fossil‐fueled development’ pathway (SSP5‐8.5), taken for the 2050–2060 and 2090–2100 decades. For the decade from 2010 to 2020 (hereinafter referred to as ‘current conditions’) we relied on two different CMEMS products: the ‘Global Ocean Physics Reanalysis’ (https://doi.org/10.48670/moi‐00021) for salinity and temperature and the ‘Surface Ocean Carbon Fields’ (https://doi.org/10.48670/moi‐00047) for acidity. Since the two databases provided rasters with differing resolutions and grid alignments, all layers were aligned and resampled at a 1° resolution, matching the coarsest resolution among the datasets used.

All variables were provided at different temporal resolutions (annual or monthly) and at varying depth scales (in centimetres or metres) depending on the source. To standardise the data and reduce potential biases, we averaged each variable across the entire relevant water column—0 to 50 m for *Luria lurida*, *Naria spurca* and *Talisman scrobilator*, and 0 to 100 m for *Zonaria pyrum*. These depth intervals were selected based on published information on species‐specific habitat preferences (Cunningham Aparicio and Verdejo Giurao 2021; Doneddu 1999) and the depth information associated with the occurrences used in the model. This approach allowed us to incorporate depth‐related ecological constraints while avoiding redundancy among predictors. We then aggregated these values by decade, producing a single mean value per decade for each variable. To define the study area, the distribution of occurrence records was compared with the known species ranges reported by WoRMS (2024), GBIF (2024), OBIS (2024), and relevant literature sources (Doneddu 1999; Meyer 2003; Passamonti 2015; Smriglio et al. 2019). For each species, the minimum and maximum latitude and longitude were identified, also taking into account their presence or absence across various islands and archipelagos. A latitudinal buffer of 15° was subsequently added to the resulting range to capture potential southward and northward shifts in species distributions. Finally, deep‐sea areas exceeding 5000 m in depth were excluded from the study area as ecologically irrelevant for the species we are considering.

Variables were tested for collinearity problems based on the Variance Inflation Factor (VIF) using the ‘usdm’ package (Naimi et al. 2014) in R and ensuring that VIFs ≤ 2 (Thompson et al. 2017) (Table 2).

### 2.3 Modelling and Data Analysis

All modelling analyses were performed with the ‘biomod2’ package (Thuiller et al. 2024) in the R environment. To model species distributions, we applied an ensemble approach (Araújo and New 2007) using the weighted average of three non‐parametric algorithms: Maxent (Phillips et al. 2006), Random Forest (RF; Breiman 2001) and Generalised Boosted Model (GBM; Elith et al. 2008). All algorithms were run with the ‘Bigboss’ set of tuning parameters (Thuiller et al. 2024). Since different algorithms can be based on different underlying assumptions and choosing between them can be challenging, ensemble models have been used to represent the general trend in several taxonomic groups (e.g., Benedetti et al. 2021; La Montagna et al. 2023; Loiseau et al. 2020). To better approximate the species' fundamental niche, we applied a multi‐temporal calibration approach (*sensu* Maiorano et al. 2013) by linking each occurrence to the mean environmental conditions in the decade it was recorded. For past periods (1850–2010), separate models were fitted for each decade, each incorporating all occurrence records available up to that period. For the present (2010–2020), a single model was fitted using all available occurrence records (1850–2020). Each model was projected onto the environmental layers of the corresponding period, and the present‐day model was additionally used to project species distribution under future climate scenarios.

Since only presences were available for the selected species, we generated pseudo‐absences using the random method. The total number of pseudo‐absences per algorithm was determined based on Barbet‐Massin et al. (2012) recommendations: 10,000 for Maxent, and a number equal to the available presences for RF and GBM. The environmental conditions at each pseudoabsence location were sampled across different decades, with their temporal distribution proportional to that of the presences. Presences and pseudo‐absences were given the same weights during modelling.

Models were cross‐validated with the k‐fold method implemented in biomod2 (*k* = 10). Data were split randomly into 10 equal‐sized subsets, each successively used for validation while the remaining nine were used for calibration, resulting in 10 calibration/validation runs (90% calibration, 10% validation). Variable importance was assessed using a permutation‐based approach, in which each predictor was randomly permuted 30 times. Model validation was performed with the True Skill Statistic (TSS) and Area Under the Curve (AUC). Only models with AUC ≥ 0.8 were averaged to compute the ensemble forecast. Continuous maps of probability of occurrence were projected for each decade from 1850 to 2020 and for 2050–2060 and 2090–2100 future scenarios. These maps were binarized in Habitat Suitability Maps using the threshold that maximises the TSS value and were used to assess potential range changes in time by calculating the proportion of suitable cells within the total study area for each species across the decades considered.

Binary Suitability Maps were also used to quantify potential large‐scale distributional shifts between past (1850–2010), current (2010–2020) and future (2050–2060 and 2090–2100, under both SSP2‐4.5 and SSP5‐8.5 scenarios) conditions. All raster suitable cells (value = 1) were converted into point coordinates and projected to an equal‐area coordinate reference system (EPSG:6933). Centroids of each group were then calculated as the arithmetic mean of all projected x and y coordinates, providing a comparable measure of the spatial “centre of mass” of the species' predicted range, and then reprojected to geographic coordinates (WGS84, EPSG:4326) for further analysis. Centroids displacement was computed as geodesic distance (in kilometres) using the ‘geosphere’ package in R (Hijmans 2026). Directionality was expressed as geographic bearing.

To assess whether areas environmentally suitable for adults may also remain viable for early life stages under future ocean conditions, we also considered the information available on larval sensitivity to ocean acidification. No physiological data are available for our four target species. Although numerous experimental studies have investigated the effects of acidification on marine molluscs, studies identifying explicit pH thresholds and their corresponding effects on larval stages remain largely confined to bivalves, whereas comparable evidence for marine gastropods is still scarce. Given the substantial biological and ecological differences between these groups, we considered that extrapolating sensitivity thresholds from bivalves to gastropods could be misleading. In addition, the use of different pH scales across studies often hampers direct comparison of results. Among the limited literature available for marine gastropods, the study by Kimura et al. (2011) on the benthic gastropod *Haliotis discus hannai* represents one of the few providing explicit pH thresholds linked to larval performance. This subspecies lives in cold‐temperate waters of northern‐central Japan, part of the Korean peninsula and of the eastern China Sea (Sun et al. 2024), with sea temperature ranges very similar to those of the Mediterranean and eastern Atlantic. We therefore adopted these thresholds as a reference, based on experiments exposing early larvae to varying pCO_2_ levels and corresponding pH conditions, while recognising that this species may not fully represent the sensitivities of those under study. The first experiment showed no detectable effects on fertilisation or early development down to pH 7.73, whereas the second experiment revealed the onset of developmental impairment at pH 7.54 and severe effects at pH 7.43. These experimentally derived values were used as biological reference points to define pH tolerance classes. To enable classification and avoid discontinuities between classes, thresholds were slightly rounded to two decimal values and organised into contiguous intervals. Accordingly, we defined four biologically informed pH classes: (i) no impact (pH ≥ 7.70), (ii) light impact (7.55 ≤ pH < 7.70), (iii) medium impact (7.44 ≤ pH < 7.55) and (iv) severe impact (pH < 7.44). These classes should be interpreted as exploratory categories of potential larval stress rather than exact physiological thresholds, and are intended to provide an indication of vulnerability under future conditions rather than precise predictions of larval tolerance. Future potential distributions (CMIP6, SSP2‐4.5 and SSP5‐8.5, decades 2050–2060 and 2090–2100) of the four species were reclassified into the four tolerance categories described above, producing categorical maps of larval risk levels restricted to areas suitable for adult populations. These maps allowed us to identify spatial mismatches between adult habitat suitability and larval viability under future acidification scenarios.

## 3 Results

A total of 5412 occurrences were gathered (Table S1), of which 3814 were used for modelling the past, current and future distribution of the four species (Table 1). RF was the algorithm that performed best on average (average AUC: 0.95; average TSS: 0.77), followed by GBM (average AUC: 0.95; average TSS: 0.63) and Maxent (average AUC: 0.83; average TSS: 0.54) (Table S2).

**FIGURE 2:**
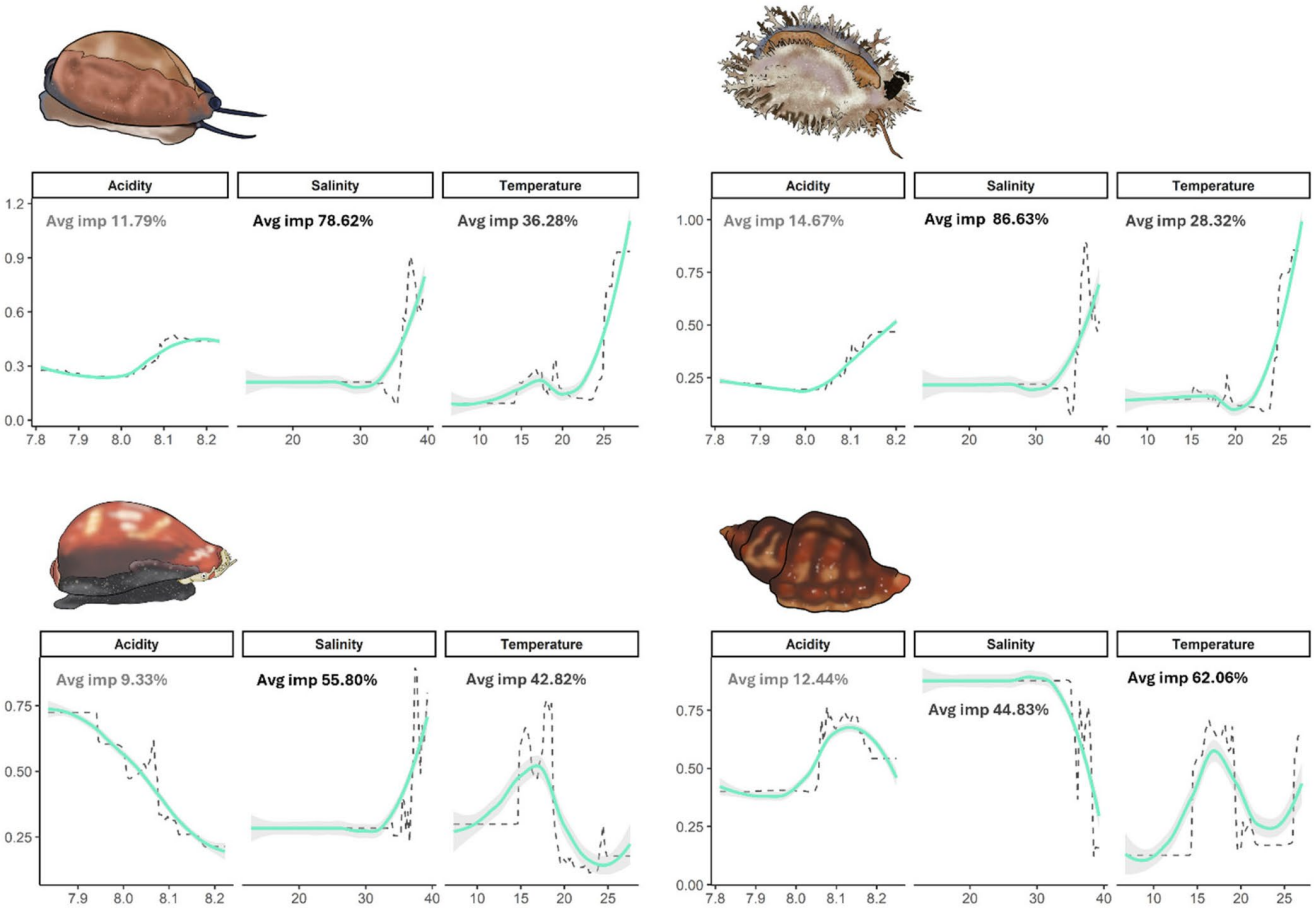
Species response curves and average variable importance (Avg imp). The importance of the variables for each species is visually represented, ranging from bold (indicating greater importance) to increasingly transparent (indicating lesser importance). From top left to bottom right, the species are *Luria lurida*, *Naria spurca*, *Zonaria pyrum*, *Talisman scrobilator*. [Species illustrations by Noemi D'Ottavi].

Salinity and temperature proved to be the two most important variables for all four species (Figure 2), but with different levels of relevance. In particular, *Luria lurida* and *Naria spurca* are primarily influenced by salinity, with an average percentage of importance of 78.62% and 86.63% respectively, far ahead of both temperature ( *L. lurida* : 36.28%; *N. spurca*: 28.32%) and acidity ( *L. lurida* : 11.79%; *N. spurca*: 14.67%). The difference in importance between salinity and temperature is less marked in the other two species. In *Zonaria pyrum*, salinity is still the most important predictor (55.80%), but it is immediately followed by temperature (42.82%). *Talisman scrobilator* is the only species in which the main predictor is temperature, with an average importance of 62.06%, followed by salinity (44.83%). Even in the last two species, acidity is of very limited importance (*Zonaria pyrum*: 9.33%; *Talisman scrobilator*: 12.44%). All species are associated with slightly alkaline waters (between pH 7.8 and 8.5), with a positive response above pH 8 for *Luria lurida*, *Naria spurca* and *Talisman scrobilator*, while *Zonaria pyrum* shows the opposite trend. The response to salinity is similar in the three cowries, with a clear preference for salinity > 30 S, while the response curve of the frog‐shell *Talisman scrobilator* shows a plateau between 10 S and 30 S, with a subsequent decrease in suitability at values above this threshold. The response to temperature is similar in *Luria lurida* and *Naria spurca*, with increasing suitability above 20°C, and in *Zonaria pyrum* and *Talisman scrobilator*, which show peak suitability between approximately 15°C and 20°C.

Considering past and current conditions (1850–2020; Figure S1), projections for *Luria lurida* show two sectors of high probability of occurrence: the northern Mediterranean, including most of the southern coast of France, the entire Italian coast and the Aegean Sea and the northern coast of the Gulf of Guinea. Over the last few decades, there has been a noticeable increase in suitability along the southern coast of the Mediterranean Sea and in the Levantine Basin. Environmental suitability is also stable in the Madeira Archipelago, part of the Canary Islands, Cape Verde and Ascension Island, while in the 2010–2020 decade the species loses suitability in St Helena. In the Azores, suitability is highly variable over time and always consists of very limited areas. In all future scenarios, most of the north‐western Mediterranean, the Azores, Madeira and the Canary Islands are no longer suitable for the species, which instead remains in the south‐eastern sector, while a new suitable area appears at higher latitudes in the Atlantic Ocean. For *Naria spurca* (Figure S2), environmental suitability remains constant throughout the Mediterranean basin, the Canary Islands, Cape Verde, Ascension Island and Saint Helena in both past and present conditions. However, it shows greater fragmentation and instability along the African coast. Under future conditions, the suitability decreases from most of the Atlantic Ocean and the western Mediterranean. In 2090, new suitable areas are predicted at higher latitudes in the Atlantic Ocean. Suitable areas for *Zonaria pyrum* (Figure S3) are limited to the Mediterranean basin until 2010, with sporadic potential occurrences in the Atlantic emerging only in the early 20th century. For this species, a potential expansion of the suitable range in the Atlantic Ocean is modelled in all future scenarios, covering higher latitudes already in the 2050–2060 ‘Fossil‐fueled development’ scenario. For *Talisman scrobilator* (Figure S4), projections indicate high suitability in the western Mediterranean basin and along the African coast south of Dakar under both past and present conditions. Around the first decades of the 20th century, suitable areas also appeared in Cape Verde and the Canary Islands, while around the second half of the century, new suitable areas appeared in Madeira, the Azores and the northern Aegean Sea. Under future conditions, the species loses suitability in almost the entire Mediterranean Sea, while suitability will remain along the African coast south of Dakar and in Cape Verde. At the same time, new suitable areas appear further north in the Atlantic Ocean.

**FIGURE 3:**
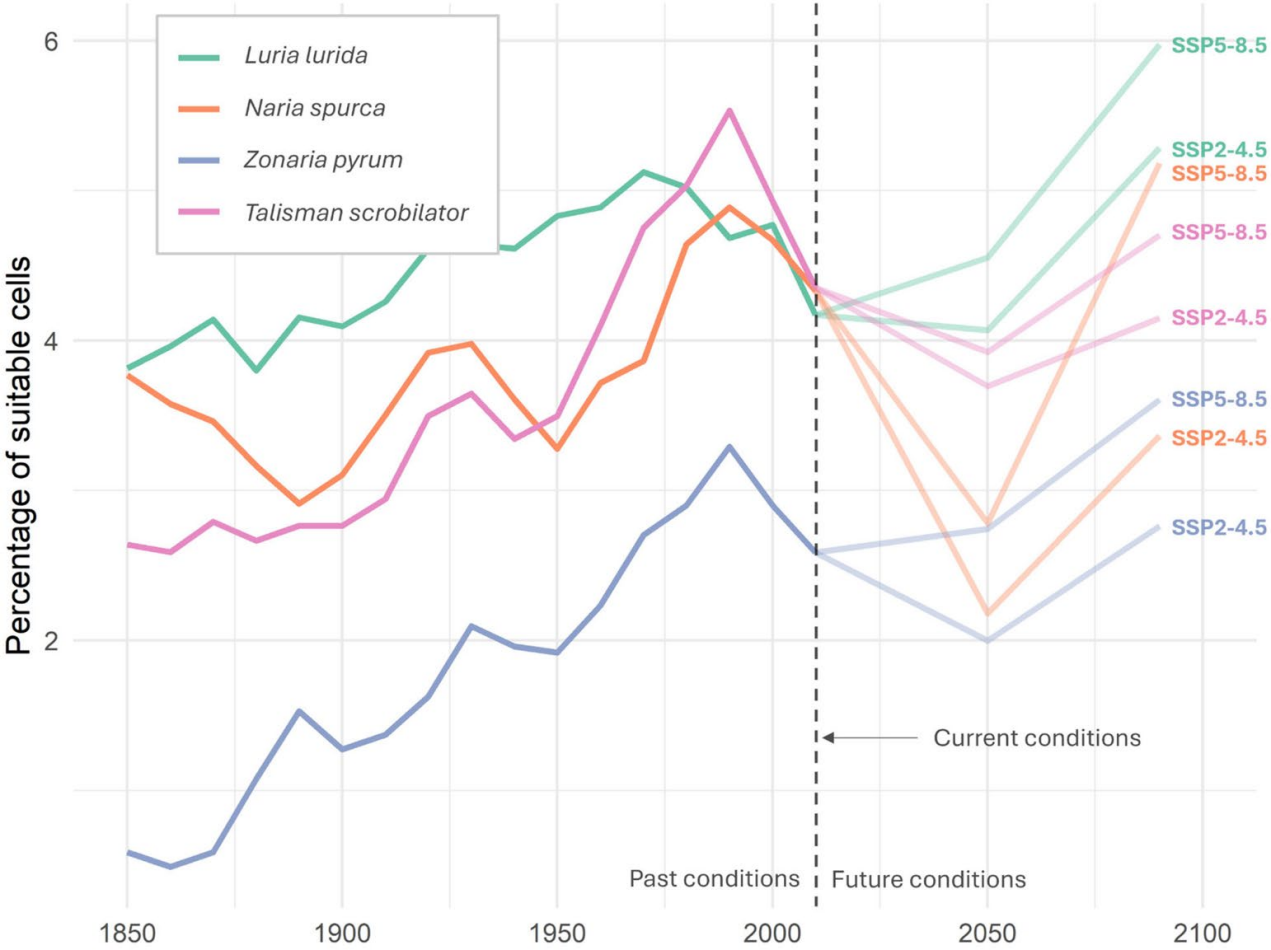
Percentage of suitable cells over time for each species, considering past, present and future (‘Middle of the road’ SSP2‐4.5 and ‘Fossil‐fueled development’ SSP5‐8 scenarios) conditions.

For each species, the proportion of suitable habitat within the total study area across time is represented in Figure 3. All suitable ranges exhibited an overall expansion trend from 1850 to the early 21st century, reaching its peak between the 1970s and the first decade of the new millennium, followed by a marked contraction in the 2010–2020 decade. The ‘Fossil‐fueled development’ scenarios proved to be more favourable for all species compared to the ‘Middle of the road’ scenario, with larger suitable areas. The suitable ranges of *Talisman scrobilator*, and particularly *Naria spurca*, undergo a sharp decline between the present and the 2050–2060 decade, followed by a recovery in the 2090–2100 period. The area suitable for the species *Luria lurida* remains relatively stable in the decade 2050–2060 and then expands in 2090–2100. In 2050–2060, *Zonaria pyrum* experiences a very favourable SSP5‐8.5 scenario compared to a stable SSP2‐4.5 one, while in 2090–2100 a new expansion takes place.

**FIGURE 4:**
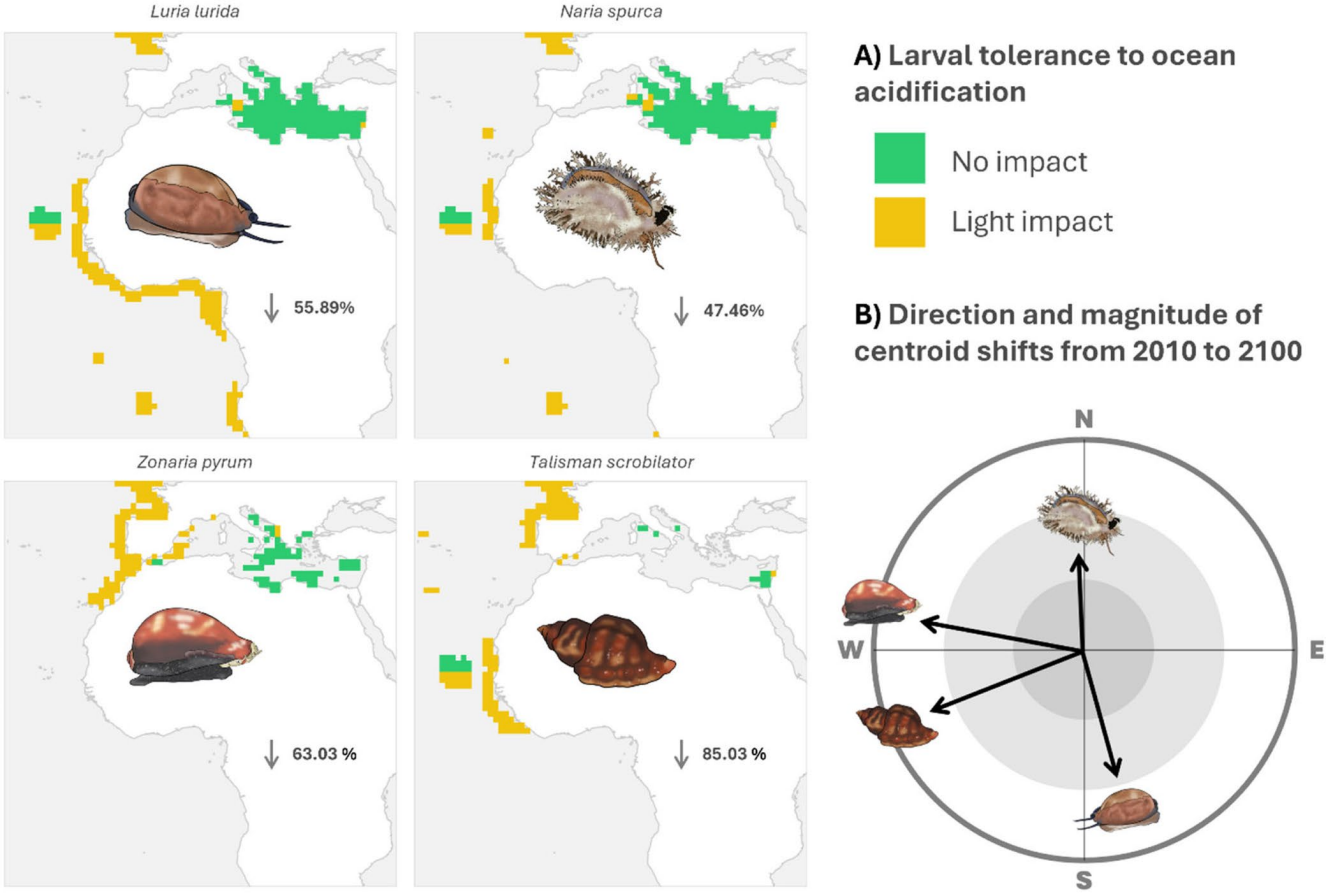
(A) Spatial mismatches between adult habitat suitability (the entire coloured area) and larval viability (no impact: PH ≥ 7.70, green; light impact: 7.55 ≤ pH < 7.70, yellow) in the 2090–2100 SSP5‐8.5 scenario, incorporating larval tolerance to ocean acidification. No areas with medium impact (7.44 ≤ pH < 7.55) or severe impact (pH < 7.44) were found. Each map displays the percentage reduction in the species' potential suitable area after excluding regions classified as having light larval impact. (B) Shift direction and distance of potential distribution centroids between current (2010–2020) and future (2090–2100) conditions under the SSP5‐8.5 scenario. [Species illustrations by Noemi D'Ottavi].

The analysis of centroid displacements revealed pronounced species‐specific differences in both the magnitude and direction of distributional shifts (Table S3; Figure 4B). From the historical to the current period, centroid positions shifted consistently northward for all species, with the exception of *Zonaria pyrum*, which remained largely stable. This shared pattern suggests a common baseline response, while more pronounced divergences among species become evident under future scenarios. *Luria lurida* exhibited consistent southward shifts under both scenarios. Displacement ranged from 723 km (SSP5‐8.5) to 783 km (SSP2‐4.5) by 2090–2100, with trajectories oriented towards the south to south‐southeast. Under mid‐century conditions, movements were even larger under SSP2 (~1019 km southward), while SSP5 showed a slightly reduced displacement (~791 km, south‐southwest). *Naria spurca* displayed more variable responses depending on the scenario. Under SSP2‐4.5, centroid shifts were predominantly eastward to east‐northeast (494 km by 2090–2100), whereas under SSP5‐8.5 the long‐term trajectory shifted northward (~548 km). Mid‐century projections showed a strong eastward displacement under SSP2‐4.5 (~738 km) and a weaker east‐northeast movement under SSP5‐8.5 (~326 km). In contrast, *Zonaria pyrum* exhibited a marked westward displacement, particularly under SSP5‐8.5. By 2090–2100, centroid shifts reached ~618 km under SSP2‐4.5 (west‐northwest) and increased to ~908 km under SSP5‐8.5 (westward). Mid‐century patterns differed between scenarios, with limited displacement under SSP2‐4.5 (~150 km south‐southeast) but a pronounced westward shift under SSP5‐8.5 (~553 km). *Talisman scrobilator* showed the largest overall centroid displacements among the four taxa. Under both scenarios, shifts were consistently oriented towards the west‐southwest. By 2090–2100, centroid displacement reached approximately 1083 km under SSP2‐4.5 and 899 km under SSP5‐8.5. Similarly, mid‐century projections indicated substantial movements exceeding 1100 km under both scenarios.

Across all scenarios, areas identified as suitable for adults largely remained above the threshold for larval viability (Figure S5), with impacts emerging only under the most severe future conditions (Figure 4A). In particular, regions classified as experiencing light larval impact (pH 7.55–7.69) appeared exclusively under the 2090–2100 SSP5‐8.5 scenario, while no areas reached the medium or severe impact thresholds (pH < 7.55). When excluding cells with light impact and retaining only those fully within the no impact category (pH ≥ 7.70), the extent of larval‐viable potential distribution was reduced substantially. Under the 2090–2100 SSP5‐8.5 scenario, the proportion of the areas suitable for adults that remained viable also for the larvae was 44.12% for *Luria lurida* (55.89% reduction), 52.53% for *Naria spurca* (47.46% reduction), 36.96% for *Zonaria pyrum* (63.03% reduction) and 14.96% for *Talisman scrobilator* (85.03% reduction) (Figure 4A).

## 4 Discussion

By integrating nearly two centuries of data into multi‐temporal species distribution models, this study uncovers a striking pattern: intermediate climate change scenarios—both in terms of timeframe (e.g., the relatively near future of 2050–2060 compared with the more distant 2090–2100) and Socioeconomic Pathways (e.g., the less extreme SSP2‐4.5, representing a moderate scenario, compared with the high‐emission SSP5‐8.5)—may represent a ‘worst‐case’ transition for some marine gastropods, resulting in habitat contractions within the current distributions without compensatory gains outside (Figure 3). Furthermore, the actual viability of the areas remaining or becoming suitable is uncertain, as the portion of habitat capable of supporting the complete life cycle of these species is likely to be much smaller than what is suggested by the correlative models (Figure 4A).

Overall, the combination of multi‐temporal SDMs with occurrences from NHCs and historical literature allowed the use of nearly two centuries of data. These records are often underutilised in ecological forecasting (Smith et al. 2023), but can be especially valuable for all those taxa (like molluscan) which are well represented in legacy collections (Heppell 1995). However, such opportunistic data sources are often affected by spatial sampling biases (García‐Roselló et al. 2023). Although we mitigated these effects through data cleaning and spatial thinning, more advanced approaches to account for uneven sampling effort were not implemented, and results should therefore be interpreted with caution. Multi‐temporal SDMs, by capturing a wider range of adaptive responses species have exhibited through time if compared to traditional SDMs, provide a powerful framework for predicting future responses to global change (e.g., Mancino et al. 2022). Salinity and temperature emerged as primary environmental drivers (Figure 2) of the distributions of the four species we considered, confirming their critical roles in shaping marine gastropods suitable habitats, as already shown in other studies (Gallagher and Albano 2023; Mellin et al. 2012; Saupe et al. 2014; Schultz et al. 2023).

### 4.1 Species Responses to Environmental Variables

*Luria lurida* and *Naria spurca* are particularly dependent on salinity, reflecting their affinity for high‐salinity marine environments. While temperature is widely recognised as the primary environmental factor governing the broad‐scale biogeographical distribution of marine organisms (Bosch et al. 2018), salinity has often been studied for its role in determining local communities of organisms (De Jesús‐Carrillo et al. 2020; Selly et al. 2018), but much less for its large‐scale effects induced by climate change (Treherne 1985). In the Mediterranean Sea, salinity follows a longitudinal gradient, with the highest values occurring in the easternmost regions. The basin is projected to experience further salinisation over the course of the 21st century (Parras‐Berrocal et al. 2020). By contrast, salinity in the Atlantic Ocean is strongly modulated by oceanic circulation patterns (Frazão et al. 2022; Mason et al. 2011) and the inflow of freshwater from major African river systems. Although changes in salinity are expected across the Atlantic, they are likely to be more spatially variable and less extreme than those anticipated in semi‐enclosed seas such as the Mediterranean (Bindoff et al. 2019).

In both *Talisman scrobilator* and *Zonaria pyrum*, temperature appears to be a particularly important limiting factor, especially in the context of climate change, with a narrow peak of high suitability between 15°C and 20°C average water temperature (Figure 2).

The importance of pH in determining the distribution of all four species is limited (Figure 2). This is consistent with the notion that adult molluscs can often tolerate moderately reduced pH, whereas early life‐history stages are generally more sensitive to acidification (Gazeau et al. 2013; Parker et al. 2013). It is important to note that the SDMs were performed using only data of adult individuals, and, consequently, the effects of acidity derived from our analyses do not account for the vulnerability observed in juveniles.

It is important to consider that environmental stressors may interact in complex and potentially non‐linear ways. For example, the combined effects of ocean warming and acidification can generate highly variable biological responses across taxonomic groups and life‐history stages. However, the interaction between these stressors generally results in stronger biological effects than those observed under each stressor in isolation. Molluscs appear to exhibit negative responses when exposed to the combined effects of warming and acidification compared to either stressor alone (Harvey et al. 2013). The modelling framework adopted here does not explicitly account for mechanistic interactions among stressors or for potential emergent responses under novel environmental conditions and should be interpreted with caution. Addressing such interactions would require experimental or mechanistic data, which are currently lacking at the spatial and temporal scales considered in this study. Another limitation of this work lies in the use of decadal mean values rather than extreme conditions. Acute environmental events are often responsible for mortality in marine organisms (e.g., Gatì et al. 2024) and may therefore play a key role in shaping species responses. In this study, decadal means were adopted primarily to ensure comparability across datasets, harmonising present‐day environmental data (from CMEMS) with historical reconstructions derived from CMIP6 GCMs, which are inherently subject to higher levels of uncertainty and bias due to technical and modelling limitations (Kwiatkowski et al. 2020).

### 4.2 Range Changes Between Current and Future Conditions

The current (2010–2020) potential distribution for *Talisman scrobilator* (Figure S4) aligns well with most recent observations that, despite the rarity of the species, witness for a consistent presence in the coastal and insular eastern Atlantic Ocean (Vega Luz 1996) but also in the western Mediterranean Sea (Cunningham Aparicio and Verdejo Giurao 2021; Smriglio et al. 2019; Tarruella Ruestes and Lopez Soriano 2004; Lopez Soriano and Tarruella Ruestes 2002). The presence of the species in the Adriatic Sea is certainly more scattered (Smriglio et al. 2019), and becomes very sporadic in the Aegean Sea (Poursanidis & Crocetta, in Santin et al. 2021). For the other three species, their current distribution (Figures S1–S3) is well documented throughout the Mediterranean and along the western coast of Africa from Morocco to Angola (Meyer 2003). The potential distribution of all species along the oceanic coasts after the Strait of Gibraltar going northwards is limited by low temperatures, while southwards the less marked suitability could be a consequence of the freshwater supply of the large African rivers and various oceanic currents altering chemical and environmental conditions of the coastline (Frazão et al. 2022; Mason et al. 2011).

Future projections indicate that the ‘Middle of the road’ scenario (SSP2‐4.5) appears particularly unfavourable for all species (Figure 3), as environmental conditions are in an intermediate phase where some areas are no longer as favourable as in the recent past, but higher latitudes have not yet warmed enough to allow the species to thrive. In fact, the broader suitability under SSP5‐8.5 is primarily driven by the emergence of new areas with favourable environmental conditions—particularly temperature—at higher latitudes in the Atlantic Ocean (Figures S1–S4). This pattern is consistent with the well‐documented poleward expansion of marine thermophilous species in response to sea warming (Albano 2014; Bianchi 2007; Schultz et al. 2023). Among the four species, *Talisman scrobilator* and *Naria spurca* are projected to be the most strongly affected, whereas *Luria lurida* shows only a modest reduction in potential range. *Zonaria pyrum* seems to be in a favourable position under all projected scenarios, exhibiting a general trend of suitable range expansion confirming Schultz et al. (2023) findings. In fact, whereas the first three species are mainly adapted to shallow intertidal and subtidal environments, *Zonaria pyrum* prefers deeper habitats such as the continental shelf and upper slope, increasing its potential to migrate into cooler waters below the euphotic zone and making the species more resilient to rising temperature (Schultz et al. 2023).

Both *Zonaria pyrum* and *Talisman scrobilator* showed a marked westward shift between current and 2090–2100 climate projections, with suitability increasing in the Atlantic and declining across much of the Mediterranean basin (Figure 3B; Figures S3 and S4), suggesting a redistribution towards cooler waters. The situation differs for the two species most closely associated with salinity (i.e., *Luria lurida* and *Naria spurca*), for which future scenarios indicate a persistence of suitable conditions within the Mediterranean, accompanied by an eastward shift towards more saline regions, particularly the Levantine Basin and the Aegean Sea (Figures S1 and S2). Concurrently, rising temperatures are predicted to render high latitudes and the southwestern coast of Africa suitable, where today the cold Benguela Current acts as a barrier to the dispersal of larvae (Passamonti 2015). As a result, two disjointed suitable areas would emerge: one in the Atlantic, where new suitable habitats appear, and another one in the eastern Mediterranean. As SDMs assume species equilibrium and synchronous responses to climate change—without accounting for geographic barriers, dispersal limitations, or trophic resource availability—these results cannot be interpreted as early indications of range disjunction in the two species, but it is possible to recognise the Levantine Basin as an important refugium for the local conservation of these species in the Mediterranean.

### 4.3 Dispersal Constraints in a Changing Ocean

The new suitable areas projected under the future scenarios (Figures S1–S4) raise important questions about the ability of these species to effectively shift their range (Assis et al. 2026). Indeed, the colonisation success will also depend on dispersal and on other local habitat requirements not considered within the model. For example, the availability of suitable prey species and the presence of hard, rocky substrata are essential for the persistence of populations of these species (Cunningham Aparicio and Verdejo Giurao 2021; Casoli et al. 2019).

In many marine gastropods, connectivity is largely determined by dispersal during the early life‐history stages, as adults are typically not highly mobile (Cowen and Sponaugle 2009; Ellingson and Krug 2016). The duration of the planktic larval phase—that is, the period spent in the water column before settlement—represents a key factor influencing dispersal potential. All species investigated in this study have a relatively long planktic larval stage (Crocetta et al. 2020; Passamonti 2015; Paulay and Meyer 2006), which is likely to enhance their dispersal capacity. Nevertheless, larval dispersal is largely passive, being driven by oceanic currents, waves, tidal dynamics and wind (Röhrs et al. 2014; Demmer et al. 2022). In particular, while the entry of propagules into the Mediterranean is feasible under the current circulation regime, their dispersal in the opposite direction—from the Mediterranean towards the Atlantic Ocean—is highly improbable, as the prevailing currents across the Strait of Gibraltar flow strongly eastward (Global Ocean Physics Reanalysis 2025). Similarly, escape through the eastern gateways of the basin appears unlikely. Biotic exchanges through the Suez Canal towards the Red Sea and through the Bosphorus towards the Sea of Marmara and the Black Sea are characterised by dominant flows in the opposite direction, effectively limiting outward dispersal from the Mediterranean (Global Ocean Physics Reanalysis 2025). Given that the coldest regions of the Mediterranean—potentially acting as climatic refugia—are located in semi‐enclosed sub‐basins such as the Adriatic Sea and the Gulf of Lion, which function as dispersal dead ends, the Mediterranean Sea may behave as a cul‐de‐sac for its resident species. As future environmental conditions become less favourable, long‐term persistence within the Mediterranean may be compromised, while opportunities for dispersal beyond the basin remain highly constrained (Ben Rais Lasram et al. 2010).

Furthermore, under future climate change, multiple factors may affect the dispersal potential of these species. Changes in speed and direction of currents could reshape larval transport routes, whereas increasing temperatures may alter reproductive timing and reduce the duration of larvae within the plankton (Andrello et al. 2025). For *Luria lurida* and *Naria spurca*, which are predicted to retain suitable areas in the eastern Mediterranean under future scenarios, such hydrodynamic changes could have significant effects, potentially isolating the remaining eastern populations from the Atlantic ones. Indeed, Andrello et al. (2025) modelled a marked weakening of the Tunisian Atlantic Current—one of the major pathways for Atlantic inflow towards the eastern Mediterranean—accompanied by a strengthening of the Ionian Atlantic Current in the Strait of Sicily by 2090–2100, thus exacerbating the already present geographic barriers (Pascual et al. 2017).

This uncertainty is further amplified by the lack of information on the physiological tolerances of juveniles to environmental stressors in the species examined here, a gap that is common to many organisms with larval stages, and which further limits our ability to assess the true viability of newly suitable areas. Ocean acidification has been shown to induce a wide range of biological responses in calcifying organisms, including both lethal and sublethal effects, although the magnitude and direction of these responses can vary substantially among taxa and environmental contexts (Kroeker et al. 2013; Gazeau et al. 2013). In marine molluscs, early life‐history stages are often considered particularly sensitive (e.g., Barclay et al. 2019), with experimental studies documenting reduced survival, impaired development and altered calcification. However, responses are not uniform: some studies reported negligible effects under comparable pH conditions in certain bivalve species (e.g., *Crassostrea ariakensis* in Miller et al. 2009; *Crassostrea gigas* in Frieder et al. 2017), highlighting substantial interspecific variability.

Several experimental works on other shelled benthic molluscs have demonstrated lethal effects on larvae at pH values around or below 7 (Bamber 1987; Calabrese and Davis 1966), with some exceptions reported even at higher values (Parker et al. 2011; Talmage and Gobler 2009; Van Colen et al. 2012). It is, however, well established that under low‐pH conditions (e.g., pH 7.6; Bogan et al. 2019), shell formation is impaired in early developmental stages. Since the shell represents the main defence against predation, such alterations may have indirect yet significant consequences on the survival of juvenile individuals and, ultimately, on population persistence.

Thus, while adult habitat suitability persisted under future climate projections, the incorporation of larval pH sensitivity (Kimura et al. 2011) substantially reduced the proportion of habitat that can be considered viable for juvenile stages under the 2090–2100 high‐emissions scenario (Figure 4A). This indicates that, even where adults may continue to persist, larval vulnerability to acidification could substantially limit the area effectively available for completing the life cycle. This situation may be particularly detrimental to the future persistence of *Talisman scrobilator* and *Zonaria pyrum*, which under the 2090–2100 scenarios exhibit a markedly reduced Mediterranean range (Figures S3 and S4) together with a pronounced shift towards the more acidic environments of the Atlantic Ocean (Figure 4A). At the same time, the use of *Haliotis discus hannai* as a proxy and the focus on conservative thresholds imply that our larval risk estimates are preliminary and should be interpreted as indicative rather than definitive constraints.

## 5 Conclusion

Overall, these findings provide preliminary insights on the conservation status of these species and raise potential conservation concerns for Mediterranean shelled organisms with planktic larval stages. The Mediterranean Sea lacks current‐favoured pathways towards colder regions (Global Ocean Physics Reanalysis 2025), limiting the potential for climate‐driven range shifts. Moreover, for species that already have Atlantic populations, or that are capable of dispersing beyond the Mediterranean into the Atlantic Ocean, ocean acidification is projected to be more intense in these regions (Figure 4A), potentially placing additional constraints on larval development and long‐term persistence.

If hydrodynamic and habitat conditions were to allow effective connectivity between present and newly suitable areas, it is plausible that, despite a contraction within the Mediterranean, these species could naturally reach regions offering favourable environmental conditions by the end of the century. Under such circumstances, none of the species would be at immediate risk of extinction; on the contrary, some—particularly *Zonaria pyrum* and *Luria lurida*—may benefit from climate‐driven changes, expanding into areas that will become more suitable under warmer scenarios. Nevertheless, while the persistence of viable populations in the Atlantic may ensure species‐level conservation, this could coincide with the loss of the Mediterranean evolutionary unit of *Talisman scrobilator*, as recolonisation from the Atlantic is more likely than successful dispersal in the opposite direction. Conversely, if hydrodynamic processes and larval duration fail to maintain connectivity between current and future suitable areas, the situation could become more critical, potentially requiring human‐mediated translocation interventions to preserve viable populations. Furthermore, even where temperature, salinity and pH permit survival, the availability of other essential habitat features—including adequate substrates, prey resources and conditions supporting larval survival and development—may further constrain the effective extent of suitable habitat. Consequently, although the results of this study suggest that the four species could theoretically benefit from extreme climate conditions such as those projected under the ‘Fossil‐fueled development’ (SSP5‐8.5) scenario, the actual viability of these areas remains uncertain. Should the necessary ecological and physiological conditions not co‐occur, the ultimate outcome could be a substantial contraction of potential range for all species, both within the Mediterranean and the Atlantic.

## Author Contributions

**Arianna Giannini:** conceptualization (equal), data curation (lead), formal analysis (lead), methodology (equal), visualization (lead), writing – original draft (lead), writing – review and editing (equal). **Chiara Mancino:** methodology (supporting), writing – review and editing (equal). **Luigi Maiorano:** methodology (supporting), supervision (supporting), writing – review and editing (equal). **Marco Oliverio:** conceptualization (equal), funding acquisition (lead), resources (lead), supervision (lead), writing – review and editing (equal).

## Funding

This work was supported by Programma Operativo Nazionale Ricerca e Innovazione (PON), grant number B85F21005360001 and by the European Union – NextGenerationEU, through the National Recovery and Resilience Plan (NRRP), grant number B83C22002950007 (Project code CN_00000033, National Biodiversity Future Center – NBFC).

## Conflicts of Interest

The authors declare no conflicts of interest.

## Supporting information


**Table S1:** Number of records collected from each source.
**Table S2:** Single models evaluation scores.
**Table S3:** Shift distance (km) and direction (degrees) of potential distribution centroids between past (1850–2010), current (2010–2020) and future (2050–2060 and 2090–2100, under SSP2‐4.5 and SSP5‐8.5 scenarios) conditions.
**Figure S1:** Probability of occurrence of *L. lurida* from 1850 to 2020, and for 2050–2060 and 2090–2100 future scenarios (‘Middle of the Road’ SSP2‐4.5 and ‘Fossil‐fueled Development’ SSP5‐8).
**Figure S2:** Probability of occurrence of *N. spurca* from 1850 to 2020, and for 2050–2060 and 2090–2100 future scenarios (‘Middle of the Road’ SSP2‐4.5 and ‘Fossil‐fueled Development’ SSP5‐8).
**Figure S3:** Probability of occurrence of *Z. pyrum* from 1850 to 2020, and for 2050–2060 and 2090–2100 future scenarios (‘Middle of the Road’ SSP2‐4.5 and ‘Fossil‐fueled Development’ SSP5‐8).
**Figure S4:** Probability of occurrence of *T. scrobilator* from 1850 to 2020, and for 2050–2060 and 2090–2100 future scenarios (‘Middle of the Road’ SSP2‐4.5 and ‘Fossil‐fueled Development’ SSP5‐8).
**Figure S5:** Spatial mismatches between adult habitat suitability (the entire coloured area) and larval viability (no impact: pH ≥ 7.70, green; light impact: 7.55 ≤ pH < 7.70, yellow), incorporating larval tolerance to ocean acidification. No areas with medium impact (7.44 ≤ pH < 7.55) or severe impact (pH < 7.44) were found.

## Data Availability

Datasets and codes are available from the Zenodo repository and can be accessed at: https://doi.org/10.5281/zenodo.18200589.
