# Supplementary material for: Will the Mediterranean Sea Be a Cul‐de‐Sac for Marine Gastropods Under Climate Change?
Source: Ecol Evol. 2026 Jun 9;16(6):e73677. doi: 10.1002/ece3.73677 (PMC13249805; doi:10.1002/ece3.73677)
Supplement: Supplementary file 1 — Table S1: Number of records collected from each source. Table S2: Single models evaluation scores. Table S3: Shift distance (km) and direction (degrees) of potential distribution centroids between past (1850–2010), current (2010–2020) and future (2050–2060 and 2090–2100, under SSP2‐4.5 and SSP5‐8.5 scenarios) conditions. Figure S1: Probability of occurrence of L. lurida from 1850 to 2020, and for 2050–2060 and 2090–2100 future scenarios (‘Middle of the Road’ SSP2‐4.5 and ‘Fossil‐fueled Development’ SSP5‐8). Figure S2: Probability of occurrence of N. spurca from 1850 to 2020, and for 2050–2060 and 2090–2100 future scenarios (‘Middle of the Road’ SSP2‐4.5 and ‘Fossil‐fueled Development’ SSP5‐8). Figure S3: Probability of occurrence of Z. pyrum from 1850 to 2020, and for 2050–2060 and 2090–2100 future scenarios (‘Middle of the Road’ SSP2‐4.5 and ‘Fossil‐fueled Development’ SSP5‐8). Figure S4: Probability of occurrence of T. scrobilator from 1850 to 2020, and for 2050–2060 and 2090–2100 future scenarios (‘Middle of the Road’ SSP2‐4.5 and ‘Fossil‐fueled Development’ SSP5‐8). Figure S5: Spatial mismatches between adult habitat suitability (the entire coloured area) and larval viability (no impact: pH ≥ 7.70, green; light impact: 7.55 ≤ pH < 7.70, yellow), incorporating larval tolerance to ocean acidification. No areas with medium impact (7.44 ≤ pH < 7.55) or severe impact (pH < 7.44) were found. [file ECE3-16-e73677-s001.docx]

# Supporting information

## Tables

**Table S1** Number of records collected from each source. For institutions, their institution codes from the Global Registry of Scientific Collections (Grosjean et al., 2022) were used: MNHN – Muséum national d'Histoire naturelle, Paris; MZRO – Museo civico di Zoologia di Roma; MZUB – Museo di Zoologia dell'Alma Mater Studiorum Università di Bologna; MZUR – Museo di Zoologia di Sapienza Università di Roma; MLKS – Museo Malakos, Città di Castello.

| **Source** | ***L. lurida*** | ***N. spurca*** | ***Z. pyrum*** | ***T. scrobilator*** |
| --- | --- | --- | --- | --- |
|  |  |  |  |  |
| **Institutions** |  |  |  |  |
| MLKS | 15 | 17 | 16 | 1 |
| MNHN | 0 | 0 | 0 | 18 |
| MZRO (Meli, Monterosato, Piersanti and Settepassi collections) | 129 | 56 | 26 | 21 |
| MZUB | 10 | 14 | 0 | 0 |
| MZUR (BAU collection) | 0 | 1 | 0 | 5 |
|  |  |  |  |  |
| **Private collectors** |  |  |  |  |
| Belletti C. | 3 | 2 | 3 | 0 |
| Bellucci P. | 0 | 0 | 0 | 6 |
| Bergonzoni M. | 40 | 68 | 61 | 0 |
| Delfiero E. | 0 | 0 | 0 | 7 |
| Devauchelle G. | 8 | 10 | 1 | 0 |
| Hoarau A. | 11 | 14 | 13 | 0 |
| Horst D. | 0 | 0 | 1 | 0 |
| Mariottini P. | 0 | 0 | 0 | 2 |
| Oliverio M. | 17 | 1 | 5 | 14 |
| Orsina P. | 36 | 66 | 16 | 0 |
| Ovalis P. | 0 | 0 | 0 | 2 |
| Pagli A. | 0 | 0 | 0 | 37 |
| Passamonti M. | 35 | 56 | 602 | 2 |
| Pelorce J. | 5 | 8 | 6 | 13 |
| Romani L. | 2 | 0 | 2 | 5 |
| Russo P. | 1 | 0 | 0 | 0 |
| Sanna S. | 1 | 0 | 0 | 0 |
| Sbrana C. | 0 | 0 | 0 | 12 |
| Scuderi A. | 0 | 0 | 0 | 10 |
| Templado J. | 46 | 44 | 20 | 14 |
|  |  |  |  |  |
| **Published records** |  |  |  |  |
| Arduino et al. 2010 | 18 | 6 | 19 | 7 |
| Crocetta et al. 2020 | 0 | 0 | 0 | 18 |
| Cunningham Aparicio et al., 2021 | 0 | 0 | 0 | 6 |
| GBIF.org 2024 | 1253 | 1056 | 297 | 47 |
| Giannini et al. 2024 | 97 | 135 | 33 | 15 |
| Lopez & Tarruella 2002 | 0 | 0 | 0 | 2 |
| Santin et al. 2021 | 0 | 0 | 0 | 1 |
| Shultz et al. 2024 | 268 | 271 | 138 | 0 |
| Smriglio et al. 2019 | 0 | 0 | 0 | 62 |
| Tarruella & Lopez 2004 | 0 | 0 | 0 | 2 |
| [WoRMS Editorial Board 2024](http://www.marinespecies.org/) | 0 | 0 | 0 | 4 |
|  |  |  |  |  |
| **Total collected records** | **1995** | **1825** | **1259** | **333** |

**Table S2** Single models evaluation scores.

| **Species** | **Run** | **Algorithm** | **AUC** | | **TSS** | |
| --- | --- | --- | --- | --- | --- | --- |
|  |  |  | **Cutoff** | **Score** | **Cutoff** | **Score** |
| *L. lurida* | 1 | GBM | 0.51 | 0.90 | 0.51 | 0.60 |
| *L. lurida* | 1 | MAXENT | 0.44 | 0.85 | 0.44 | 0.57 |
| *L. lurida* | 1 | RF | 0.49 | 0.97 | 0.53 | 0.82 |
| *L. lurida* | 2 | GBM | 0.50 | 0.90 | 0.51 | 0.68 |
| *L. lurida* | 2 | MAXENT | 0.44 | 0.83 | 0.43 | 0.57 |
| *L. lurida* | 2 | RF | 0.50 | 0.97 | 0.51 | 0.82 |
| *L. lurida* | 3 | GBM | 0.52 | 0.91 | 0.52 | 0.60 |
| *L. lurida* | 3 | MAXENT | 0.42 | 0.82 | 0.49 | 0.51 |
| *L. lurida* | 3 | RF | 0.52 | 0.97 | 0.53 | 0.82 |
| *L. lurida* | 4 | GBM | 0.53 | 0.87 | 0.53 | 0.60 |
| *L. lurida* | 4 | MAXENT | 0.41 | 0.83 | 0.40 | 0.48 |
| *L. lurida* | 4 | RF | 0.51 | 0.96 | 0.52 | 0.78 |
| *L. lurida* | 5 | GBM | 0.55 | 0.92 | 0.55 | 0.71 |
| *L. lurida* | 5 | MAXENT | 0.31 | 0.82 | 0.31 | 0.52 |
| *L. lurida* | 5 | RF | 0.47 | 0.97 | 0.52 | 0.83 |
| *L. lurida* | 6 | GBM | 0.52 | 0.90 | 0.52 | 0.64 |
| *L. lurida* | 6 | MAXENT | 0.42 | 0.85 | 0.41 | 0.55 |
| *L. lurida* | 6 | RF | 0.53 | 0.97 | 0.53 | 0.85 |
| *L. lurida* | 7 | GBM | 0.53 | 0.89 | 0.53 | 0.65 |
| *L. lurida* | 7 | MAXENT | 0.44 | 0.84 | 0.43 | 0.51 |
| *L. lurida* | 7 | RF | 0.49 | 0.97 | 0.51 | 0.82 |
| *L. lurida* | 8 | GBM | 0.50 | 0.92 | 0.55 | 0.71 |
| *L. lurida* | 8 | MAXENT | 0.49 | 0.78 | 0.49 | 0.42 |
| *L. lurida* | 8 | RF | 0.49 | 0.99 | 0.49 | 0.87 |
| *L. lurida* | 9 | GBM | 0.53 | 0.90 | 0.53 | 0.63 |
| *L. lurida* | 9 | MAXENT | 0.47 | 0.83 | 0.47 | 0.51 |
| *L. lurida* | 9 | RF | 0.50 | 0.98 | 0.49 | 0.83 |
| *L. lurida* | 10 | GBM | 0.51 | 0.92 | 0.51 | 0.75 |
| *L. lurida* | 10 | MAXENT | 0.42 | 0.85 | 0.42 | 0.56 |
| *L. lurida* | 10 | RF | 0.49 | 0.98 | 0.48 | 0.88 |
| *L. lurida* | 11 | GBM | 0.48 | 0.89 | 0.48 | 0.64 |
| *L. lurida* | 11 | MAXENT | 0.38 | 0.83 | 0.37 | 0.51 |
| *L. lurida* | 11 | RF | 0.53 | 0.95 | 0.53 | 0.78 |
| *L. lurida* | 12 | GBM | 0.51 | 0.90 | 0.51 | 0.64 |
| *L. lurida* | 12 | MAXENT | 0.32 | 0.83 | 0.31 | 0.50 |
| *L. lurida* | 12 | RF | 0.50 | 0.98 | 0.51 | 0.86 |
| *L. lurida* | 13 | GBM | 0.51 | 0.88 | 0.49 | 0.58 |
| *L. lurida* | 13 | MAXENT | 0.41 | 0.81 | 0.41 | 0.50 |
| *L. lurida* | 13 | RF | 0.49 | 0.96 | 0.49 | 0.77 |
| *L. lurida* | 14 | GBM | 0.48 | 0.94 | 0.51 | 0.73 |
| *L. lurida* | 14 | MAXENT | 0.47 | 0.82 | 0.47 | 0.53 |
| *L. lurida* | 14 | RF | 0.52 | 0.99 | 0.52 | 0.90 |
| *L. lurida* | 15 | GBM | 0.53 | 0.87 | 0.52 | 0.64 |
| *L. lurida* | 15 | MAXENT | 0.42 | 0.85 | 0.42 | 0.58 |
| *L. lurida* | 15 | RF | 0.50 | 0.96 | 0.51 | 0.80 |
| *L. lurida* | 16 | GBM | 0.55 | 0.91 | 0.55 | 0.69 |
| *L. lurida* | 16 | MAXENT | 0.46 | 0.85 | 0.46 | 0.54 |
| *L. lurida* | 16 | RF | 0.45 | 0.98 | 0.47 | 0.84 |
| *L. lurida* | 17 | GBM | 0.51 | 0.90 | 0.52 | 0.66 |
| *L. lurida* | 17 | MAXENT | 0.34 | 0.83 | 0.33 | 0.50 |
| *L. lurida* | 17 | RF | 0.50 | 0.96 | 0.49 | 0.84 |
| *L. lurida* | 18 | GBM | 0.51 | 0.92 | 0.51 | 0.64 |
| *L. lurida* | 18 | MAXENT | 0.48 | 0.84 | 0.47 | 0.54 |
| *L. lurida* | 18 | RF | 0.49 | 0.97 | 0.48 | 0.83 |
| *L. lurida* | 19 | GBM | 0.52 | 0.93 | 0.52 | 0.68 |
| *L. lurida* | 19 | MAXENT | 0.44 | 0.82 | 0.44 | 0.51 |
| *L. lurida* | 19 | RF | 0.47 | 0.99 | 0.47 | 0.88 |
| *L. lurida* | 20 | GBM | 0.50 | 0.89 | 0.51 | 0.66 |
| *L. lurida* | 20 | MAXENT | 0.48 | 0.80 | 0.47 | 0.44 |
| *L. lurida* | 20 | RF | 0.49 | 0.97 | 0.53 | 0.79 |
| *L. lurida* | 21 | GBM | 0.51 | 0.91 | 0.54 | 0.64 |
| *L. lurida* | 21 | MAXENT | 0.46 | 0.86 | 0.46 | 0.59 |
| *L. lurida* | 21 | RF | 0.47 | 0.97 | 0.47 | 0.83 |
| *L. lurida* | 22 | GBM | 0.52 | 0.91 | 0.51 | 0.64 |
| *L. lurida* | 22 | MAXENT | 0.48 | 0.81 | 0.47 | 0.49 |
| *L. lurida* | 22 | RF | 0.52 | 0.98 | 0.53 | 0.83 |
| *L. lurida* | 23 | GBM | 0.54 | 0.87 | 0.53 | 0.61 |
| *L. lurida* | 23 | MAXENT | 0.36 | 0.82 | 0.42 | 0.50 |
| *L. lurida* | 23 | RF | 0.51 | 0.95 | 0.49 | 0.80 |
| *L. lurida* | 24 | GBM | 0.52 | 0.93 | 0.56 | 0.69 |
| *L. lurida* | 24 | MAXENT | 0.48 | 0.83 | 0.47 | 0.55 |
| *L. lurida* | 24 | RF | 0.47 | 0.99 | 0.47 | 0.86 |
| *L. lurida* | 25 | GBM | 0.53 | 0.91 | 0.53 | 0.73 |
| *L. lurida* | 25 | MAXENT | 0.35 | 0.82 | 0.34 | 0.50 |
| *L. lurida* | 25 | RF | 0.50 | 0.98 | 0.53 | 0.84 |
| *L. lurida* | 26 | GBM | 0.53 | 0.91 | 0.53 | 0.66 |
| *L. lurida* | 26 | MAXENT | 0.41 | 0.82 | 0.41 | 0.48 |
| *L. lurida* | 26 | RF | 0.52 | 0.98 | 0.52 | 0.85 |
| *L. lurida* | 27 | GBM | 0.47 | 0.92 | 0.47 | 0.67 |
| *L. lurida* | 27 | MAXENT | 0.43 | 0.85 | 0.43 | 0.55 |
| *L. lurida* | 27 | RF | 0.49 | 0.98 | 0.48 | 0.87 |
| *L. lurida* | 28 | GBM | 0.50 | 0.88 | 0.52 | 0.59 |
| *L. lurida* | 28 | MAXENT | 0.43 | 0.86 | 0.43 | 0.58 |
| *L. lurida* | 28 | RF | 0.49 | 0.97 | 0.49 | 0.83 |
| *L. lurida* | 29 | GBM | 0.53 | 0.91 | 0.52 | 0.66 |
| *L. lurida* | 29 | MAXENT | 0.48 | 0.82 | 0.47 | 0.49 |
| *L. lurida* | 29 | RF | 0.50 | 0.97 | 0.51 | 0.79 |
| *L. lurida* | 30 | GBM | 0.47 | 0.88 | 0.48 | 0.62 |
| *L. lurida* | 30 | MAXENT | 0.42 | 0.82 | 0.41 | 0.53 |
| *L. lurida* | 30 | RF | 0.50 | 0.96 | 0.49 | 0.83 |
| *L. lurida* | 31 | GBM | 0.54 | 0.93 | 0.53 | 0.72 |
| *L. lurida* | 31 | MAXENT | 0.46 | 0.85 | 0.46 | 0.55 |
| *L. lurida* | 31 | RF | 0.46 | 0.98 | 0.48 | 0.82 |
| *L. lurida* | 32 | GBM | 0.54 | 0.88 | 0.55 | 0.58 |
| *L. lurida* | 32 | MAXENT | 0.40 | 0.81 | 0.37 | 0.45 |
| *L. lurida* | 32 | RF | 0.48 | 0.96 | 0.48 | 0.77 |
| *L. lurida* | 33 | GBM | 0.53 | 0.91 | 0.53 | 0.65 |
| *L. lurida* | 33 | MAXENT | 0.47 | 0.84 | 0.34 | 0.55 |
| *L. lurida* | 33 | RF | 0.49 | 0.97 | 0.49 | 0.80 |
| *L. lurida* | 34 | GBM | 0.52 | 0.90 | 0.51 | 0.67 |
| *L. lurida* | 34 | MAXENT | 0.46 | 0.81 | 0.47 | 0.48 |
| *L. lurida* | 34 | RF | 0.49 | 0.97 | 0.48 | 0.88 |
| *L. lurida* | 35 | GBM | 0.52 | 0.91 | 0.51 | 0.69 |
| *L. lurida* | 35 | MAXENT | 0.44 | 0.86 | 0.43 | 0.61 |
| *L. lurida* | 35 | RF | 0.50 | 0.97 | 0.51 | 0.84 |
| *L. lurida* | 36 | GBM | 0.53 | 0.89 | 0.53 | 0.63 |
| *L. lurida* | 36 | MAXENT | 0.40 | 0.84 | 0.38 | 0.56 |
| *L. lurida* | 36 | RF | 0.52 | 0.97 | 0.52 | 0.83 |
| *L. lurida* | 37 | GBM | 0.48 | 0.88 | 0.48 | 0.57 |
| *L. lurida* | 37 | MAXENT | 0.47 | 0.84 | 0.47 | 0.55 |
| *L. lurida* | 37 | RF | 0.53 | 0.98 | 0.53 | 0.85 |
| *L. lurida* | 38 | GBM | 0.51 | 0.89 | 0.51 | 0.63 |
| *L. lurida* | 38 | MAXENT | 0.41 | 0.82 | 0.40 | 0.50 |
| *L. lurida* | 38 | RF | 0.51 | 0.96 | 0.52 | 0.78 |
| *L. lurida* | 39 | GBM | 0.50 | 0.90 | 0.53 | 0.67 |
| *L. lurida* | 39 | MAXENT | 0.43 | 0.84 | 0.42 | 0.53 |
| *L. lurida* | 39 | RF | 0.49 | 0.97 | 0.48 | 0.82 |
| *L. lurida* | 40 | GBM | 0.50 | 0.94 | 0.50 | 0.73 |
| *L. lurida* | 40 | MAXENT | 0.45 | 0.82 | 0.35 | 0.46 |
| *L. lurida* | 40 | RF | 0.47 | 0.98 | 0.51 | 0.87 |
| *L. lurida* | 41 | GBM | 0.53 | 0.93 | 0.54 | 0.70 |
| *L. lurida* | 41 | MAXENT | 0.47 | 0.82 | 0.47 | 0.51 |
| *L. lurida* | 41 | RF | 0.47 | 0.97 | 0.51 | 0.84 |
| *L. lurida* | 42 | GBM | 0.51 | 0.86 | 0.50 | 0.61 |
| *L. lurida* | 42 | MAXENT | 0.32 | 0.83 | 0.39 | 0.50 |
| *L. lurida* | 42 | RF | 0.50 | 0.95 | 0.49 | 0.74 |
| *L. lurida* | 43 | GBM | 0.49 | 0.95 | 0.49 | 0.80 |
| *L. lurida* | 43 | MAXENT | 0.46 | 0.82 | 0.46 | 0.48 |
| *L. lurida* | 43 | RF | 0.49 | 0.98 | 0.51 | 0.88 |
| *L. lurida* | 44 | GBM | 0.52 | 0.89 | 0.52 | 0.61 |
| *L. lurida* | 44 | MAXENT | 0.45 | 0.84 | 0.44 | 0.53 |
| *L. lurida* | 44 | RF | 0.53 | 0.97 | 0.53 | 0.85 |
| *L. lurida* | 45 | GBM | 0.56 | 0.86 | 0.55 | 0.57 |
| *L. lurida* | 45 | MAXENT | 0.47 | 0.83 | 0.47 | 0.52 |
| *L. lurida* | 45 | RF | 0.48 | 0.97 | 0.48 | 0.81 |
| *L. lurida* | 46 | GBM | 0.51 | 0.93 | 0.51 | 0.70 |
| *L. lurida* | 46 | MAXENT | 0.44 | 0.81 | 0.43 | 0.49 |
| *L. lurida* | 46 | RF | 0.51 | 0.98 | 0.51 | 0.83 |
| *L. lurida* | 47 | GBM | 0.50 | 0.86 | 0.50 | 0.56 |
| *L. lurida* | 47 | MAXENT | 0.45 | 0.84 | 0.44 | 0.52 |
| *L. lurida* | 47 | RF | 0.50 | 0.96 | 0.49 | 0.80 |
| *L. lurida* | 48 | GBM | 0.50 | 0.90 | 0.50 | 0.69 |
| *L. lurida* | 48 | MAXENT | 0.45 | 0.84 | 0.44 | 0.55 |
| *L. lurida* | 48 | RF | 0.53 | 0.97 | 0.54 | 0.80 |
| *L. lurida* | 49 | GBM | 0.53 | 0.91 | 0.53 | 0.67 |
| *L. lurida* | 49 | MAXENT | 0.41 | 0.85 | 0.40 | 0.54 |
| *L. lurida* | 49 | RF | 0.51 | 0.98 | 0.52 | 0.84 |
| *L. lurida* | 50 | GBM | 0.54 | 0.92 | 0.53 | 0.69 |
| *L. lurida* | 50 | MAXENT | 0.46 | 0.84 | 0.46 | 0.57 |
| *L. lurida* | 50 | RF | 0.49 | 0.98 | 0.56 | 0.84 |
| *L. lurida* | 51 | GBM | 0.53 | 0.92 | 0.54 | 0.69 |
| *L. lurida* | 51 | MAXENT | 0.42 | 0.86 | 0.44 | 0.58 |
| *L. lurida* | 51 | RF | 0.51 | 0.97 | 0.52 | 0.83 |
| *L. lurida* | 52 | GBM | 0.54 | 0.92 | 0.51 | 0.72 |
| *L. lurida* | 52 | MAXENT | 0.41 | 0.84 | 0.40 | 0.54 |
| *L. lurida* | 52 | RF | 0.48 | 0.98 | 0.47 | 0.88 |
| *L. lurida* | 53 | GBM | 0.53 | 0.93 | 0.52 | 0.72 |
| *L. lurida* | 53 | MAXENT | 0.44 | 0.82 | 0.44 | 0.50 |
| *L. lurida* | 53 | RF | 0.48 | 0.99 | 0.48 | 0.89 |
| *L. lurida* | 54 | GBM | 0.50 | 0.90 | 0.50 | 0.67 |
| *L. lurida* | 54 | MAXENT | 0.45 | 0.81 | 0.46 | 0.49 |
| *L. lurida* | 54 | RF | 0.51 | 0.97 | 0.51 | 0.82 |
| *L. lurida* | 55 | GBM | 0.48 | 0.90 | 0.48 | 0.68 |
| *L. lurida* | 55 | MAXENT | 0.40 | 0.81 | 0.39 | 0.49 |
| *L. lurida* | 55 | RF | 0.50 | 0.98 | 0.51 | 0.86 |
| *L. lurida* | 56 | GBM | 0.52 | 0.88 | 0.53 | 0.61 |
| *L. lurida* | 56 | MAXENT | 0.48 | 0.84 | 0.47 | 0.55 |
| *L. lurida* | 56 | RF | 0.51 | 0.97 | 0.52 | 0.80 |
| *L. lurida* | 57 | GBM | 0.46 | 0.90 | 0.51 | 0.63 |
| *L. lurida* | 57 | MAXENT | 0.36 | 0.84 | 0.43 | 0.53 |
| *L. lurida* | 57 | RF | 0.49 | 0.97 | 0.49 | 0.80 |
| *L. lurida* | 58 | GBM | 0.52 | 0.90 | 0.52 | 0.69 |
| *L. lurida* | 58 | MAXENT | 0.48 | 0.83 | 0.47 | 0.51 |
| *L. lurida* | 58 | RF | 0.52 | 0.97 | 0.53 | 0.86 |
| *L. lurida* | 59 | GBM | 0.54 | 0.88 | 0.54 | 0.60 |
| *L. lurida* | 59 | MAXENT | 0.47 | 0.83 | 0.47 | 0.55 |
| *L. lurida* | 59 | RF | 0.50 | 0.96 | 0.50 | 0.77 |
| *L. lurida* | 60 | GBM | 0.51 | 0.86 | 0.51 | 0.57 |
| *L. lurida* | 60 | MAXENT | 0.34 | 0.82 | 0.34 | 0.51 |
| *L. lurida* | 60 | RF | 0.50 | 0.96 | 0.49 | 0.80 |
| *L. lurida* | 61 | GBM | 0.51 | 0.88 | 0.53 | 0.61 |
| *L. lurida* | 61 | MAXENT | 0.37 | 0.84 | 0.36 | 0.55 |
| *L. lurida* | 61 | RF | 0.50 | 0.96 | 0.51 | 0.83 |
| *L. lurida* | 62 | GBM | 0.56 | 0.92 | 0.56 | 0.68 |
| *L. lurida* | 62 | MAXENT | 0.50 | 0.81 | 0.49 | 0.47 |
| *L. lurida* | 62 | RF | 0.47 | 0.97 | 0.47 | 0.82 |
| *L. lurida* | 63 | GBM | 0.50 | 0.93 | 0.50 | 0.73 |
| *L. lurida* | 63 | MAXENT | 0.44 | 0.82 | 0.43 | 0.51 |
| *L. lurida* | 63 | RF | 0.50 | 0.98 | 0.49 | 0.87 |
| *L. lurida* | 64 | GBM | 0.50 | 0.92 | 0.49 | 0.70 |
| *L. lurida* | 64 | MAXENT | 0.46 | 0.80 | 0.46 | 0.45 |
| *L. lurida* | 64 | RF | 0.48 | 0.99 | 0.47 | 0.88 |
| *L. lurida* | 65 | GBM | 0.53 | 0.89 | 0.53 | 0.62 |
| *L. lurida* | 65 | MAXENT | 0.51 | 0.86 | 0.51 | 0.58 |
| *L. lurida* | 65 | RF | 0.48 | 0.97 | 0.52 | 0.82 |
| *L. lurida* | 66 | GBM | 0.53 | 0.89 | 0.53 | 0.66 |
| *L. lurida* | 66 | MAXENT | 0.44 | 0.83 | 0.43 | 0.54 |
| *L. lurida* | 66 | RF | 0.48 | 0.97 | 0.48 | 0.83 |
| *L. lurida* | 67 | GBM | 0.52 | 0.91 | 0.52 | 0.63 |
| *L. lurida* | 67 | MAXENT | 0.46 | 0.81 | 0.46 | 0.48 |
| *L. lurida* | 67 | RF | 0.46 | 0.98 | 0.46 | 0.84 |
| *L. lurida* | 68 | GBM | 0.55 | 0.90 | 0.54 | 0.64 |
| *L. lurida* | 68 | MAXENT | 0.47 | 0.86 | 0.44 | 0.54 |
| *L. lurida* | 68 | RF | 0.49 | 0.96 | 0.48 | 0.77 |
| *L. lurida* | 69 | GBM | 0.54 | 0.87 | 0.54 | 0.59 |
| *L. lurida* | 69 | MAXENT | 0.44 | 0.85 | 0.44 | 0.61 |
| *L. lurida* | 69 | RF | 0.48 | 0.96 | 0.48 | 0.78 |
| *L. lurida* | 70 | GBM | 0.53 | 0.92 | 0.53 | 0.67 |
| *L. lurida* | 70 | MAXENT | 0.46 | 0.84 | 0.46 | 0.56 |
| *L. lurida* | 70 | RF | 0.48 | 0.97 | 0.49 | 0.81 |
| *L. lurida* | 71 | GBM | 0.53 | 0.89 | 0.54 | 0.64 |
| *L. lurida* | 71 | MAXENT | 0.44 | 0.82 | 0.43 | 0.52 |
| *L. lurida* | 71 | RF | 0.51 | 0.97 | 0.51 | 0.85 |
| *L. lurida* | 72 | GBM | 0.54 | 0.91 | 0.53 | 0.62 |
| *L. lurida* | 72 | MAXENT | 0.45 | 0.81 | 0.47 | 0.48 |
| *L. lurida* | 72 | RF | 0.49 | 0.98 | 0.49 | 0.85 |
| *L. lurida* | 73 | GBM | 0.49 | 0.91 | 0.49 | 0.62 |
| *L. lurida* | 73 | MAXENT | 0.42 | 0.81 | 0.41 | 0.47 |
| *L. lurida* | 73 | RF | 0.49 | 0.98 | 0.50 | 0.84 |
| *L. lurida* | 74 | GBM | 0.54 | 0.88 | 0.54 | 0.65 |
| *L. lurida* | 74 | MAXENT | 0.37 | 0.85 | 0.37 | 0.52 |
| *L. lurida* | 74 | RF | 0.48 | 0.97 | 0.48 | 0.82 |
| *L. lurida* | 75 | GBM | 0.52 | 0.91 | 0.52 | 0.66 |
| *L. lurida* | 75 | MAXENT | 0.40 | 0.83 | 0.40 | 0.53 |
| *L. lurida* | 75 | RF | 0.50 | 0.98 | 0.48 | 0.85 |
| *L. lurida* | 76 | GBM | 0.52 | 0.88 | 0.52 | 0.58 |
| *L. lurida* | 76 | MAXENT | 0.41 | 0.81 | 0.40 | 0.45 |
| *L. lurida* | 76 | RF | 0.52 | 0.96 | 0.51 | 0.78 |
| *L. lurida* | 77 | GBM | 0.54 | 0.91 | 0.53 | 0.69 |
| *L. lurida* | 77 | MAXENT | 0.38 | 0.81 | 0.36 | 0.47 |
| *L. lurida* | 77 | RF | 0.48 | 0.98 | 0.48 | 0.86 |
| *L. lurida* | 78 | GBM | 0.54 | 0.91 | 0.54 | 0.72 |
| *L. lurida* | 78 | MAXENT | 0.39 | 0.84 | 0.38 | 0.55 |
| *L. lurida* | 78 | RF | 0.49 | 0.98 | 0.49 | 0.84 |
| *L. lurida* | 79 | GBM | 0.50 | 0.91 | 0.50 | 0.68 |
| *L. lurida* | 79 | MAXENT | 0.47 | 0.82 | 0.47 | 0.51 |
| *L. lurida* | 79 | RF | 0.47 | 0.98 | 0.49 | 0.83 |
| *L. lurida* | 80 | GBM | 0.52 | 0.92 | 0.50 | 0.73 |
| *L. lurida* | 80 | MAXENT | 0.44 | 0.86 | 0.43 | 0.59 |
| *L. lurida* | 80 | RF | 0.51 | 0.97 | 0.51 | 0.83 |
| *L. lurida* | 81 | GBM | 0.50 | 0.89 | 0.50 | 0.60 |
| *L. lurida* | 81 | MAXENT | 0.34 | 0.84 | 0.32 | 0.53 |
| *L. lurida* | 81 | RF | 0.49 | 0.96 | 0.49 | 0.79 |
| *L. lurida* | 82 | GBM | 0.52 | 0.90 | 0.53 | 0.66 |
| *L. lurida* | 82 | MAXENT | 0.39 | 0.81 | 0.39 | 0.47 |
| *L. lurida* | 82 | RF | 0.51 | 0.97 | 0.51 | 0.83 |
| *L. lurida* | 83 | GBM | 0.55 | 0.92 | 0.55 | 0.67 |
| *L. lurida* | 83 | MAXENT | 0.48 | 0.83 | 0.47 | 0.52 |
| *L. lurida* | 83 | RF | 0.52 | 0.98 | 0.52 | 0.85 |
| *L. lurida* | 84 | GBM | 0.52 | 0.91 | 0.52 | 0.69 |
| *L. lurida* | 84 | MAXENT | 0.42 | 0.82 | 0.41 | 0.56 |
| *L. lurida* | 84 | RF | 0.51 | 0.98 | 0.51 | 0.85 |
| *L. lurida* | 85 | GBM | 0.52 | 0.90 | 0.52 | 0.64 |
| *L. lurida* | 85 | MAXENT | 0.49 | 0.86 | 0.48 | 0.60 |
| *L. lurida* | 85 | RF | 0.52 | 0.97 | 0.53 | 0.83 |
| *L. lurida* | 86 | GBM | 0.51 | 0.91 | 0.51 | 0.68 |
| *L. lurida* | 86 | MAXENT | 0.41 | 0.86 | 0.42 | 0.56 |
| *L. lurida* | 86 | RF | 0.49 | 0.98 | 0.51 | 0.85 |
| *L. lurida* | 87 | GBM | 0.54 | 0.91 | 0.54 | 0.67 |
| *L. lurida* | 87 | MAXENT | 0.48 | 0.83 | 0.48 | 0.55 |
| *L. lurida* | 87 | RF | 0.52 | 0.98 | 0.49 | 0.84 |
| *L. lurida* | 88 | GBM | 0.53 | 0.88 | 0.53 | 0.60 |
| *L. lurida* | 88 | MAXENT | 0.37 | 0.84 | 0.37 | 0.55 |
| *L. lurida* | 88 | RF | 0.47 | 0.97 | 0.46 | 0.80 |
| *L. lurida* | 89 | GBM | 0.51 | 0.90 | 0.51 | 0.68 |
| *L. lurida* | 89 | MAXENT | 0.50 | 0.82 | 0.49 | 0.48 |
| *L. lurida* | 89 | RF | 0.51 | 0.97 | 0.51 | 0.82 |
| *L. lurida* | 90 | GBM | 0.51 | 0.92 | 0.53 | 0.68 |
| *L. lurida* | 90 | MAXENT | 0.38 | 0.79 | 0.38 | 0.45 |
| *L. lurida* | 90 | RF | 0.46 | 0.98 | 0.47 | 0.83 |
| *L. lurida* | 91 | GBM | 0.50 | 0.88 | 0.50 | 0.57 |
| *L. lurida* | 91 | MAXENT | 0.46 | 0.85 | 0.46 | 0.60 |
| *L. lurida* | 91 | RF | 0.50 | 0.96 | 0.51 | 0.76 |
| *L. lurida* | 92 | GBM | 0.53 | 0.90 | 0.53 | 0.61 |
| *L. lurida* | 92 | MAXENT | 0.41 | 0.86 | 0.40 | 0.58 |
| *L. lurida* | 92 | RF | 0.47 | 0.97 | 0.49 | 0.82 |
| *L. lurida* | 93 | GBM | 0.52 | 0.94 | 0.52 | 0.74 |
| *L. lurida* | 93 | MAXENT | 0.48 | 0.83 | 0.47 | 0.55 |
| *L. lurida* | 93 | RF | 0.51 | 0.98 | 0.51 | 0.88 |
| *L. lurida* | 94 | GBM | 0.53 | 0.92 | 0.53 | 0.67 |
| *L. lurida* | 94 | MAXENT | 0.49 | 0.85 | 0.48 | 0.56 |
| *L. lurida* | 94 | RF | 0.49 | 0.98 | 0.48 | 0.85 |
| *L. lurida* | 95 | GBM | 0.54 | 0.88 | 0.54 | 0.64 |
| *L. lurida* | 95 | MAXENT | 0.49 | 0.82 | 0.48 | 0.48 |
| *L. lurida* | 95 | RF | 0.49 | 0.95 | 0.49 | 0.78 |
| *L. lurida* | 96 | GBM | 0.55 | 0.90 | 0.54 | 0.71 |
| *L. lurida* | 96 | MAXENT | 0.38 | 0.81 | 0.37 | 0.51 |
| *L. lurida* | 96 | RF | 0.48 | 0.97 | 0.50 | 0.84 |
| *L. lurida* | 97 | GBM | 0.49 | 0.91 | 0.50 | 0.69 |
| *L. lurida* | 97 | MAXENT | 0.43 | 0.81 | 0.42 | 0.52 |
| *L. lurida* | 97 | RF | 0.52 | 0.97 | 0.52 | 0.85 |
| *L. lurida* | 98 | GBM | 0.52 | 0.89 | 0.52 | 0.69 |
| *L. lurida* | 98 | MAXENT | 0.48 | 0.83 | 0.48 | 0.55 |
| *L. lurida* | 98 | RF | 0.48 | 0.98 | 0.47 | 0.84 |
| *L. lurida* | 99 | GBM | 0.48 | 0.90 | 0.48 | 0.66 |
| *L. lurida* | 99 | MAXENT | 0.41 | 0.80 | 0.41 | 0.46 |
| *L. lurida* | 99 | RF | 0.51 | 0.98 | 0.52 | 0.84 |
| *L. lurida* | 100 | GBM | 0.55 | 0.89 | 0.55 | 0.64 |
| *L. lurida* | 100 | MAXENT | 0.39 | 0.82 | 0.39 | 0.48 |
| *L. lurida* | 100 | RF | 0.47 | 0.98 | 0.47 | 0.84 |
| *N. spurca* | 1 | GBM | 0.63 | 0.93 | 0.63 | 0.67 |
| *N. spurca* | 1 | MAXENT | 0.26 | 0.81 | 0.25 | 0.55 |
| *N. spurca* | 1 | RF | 0.46 | 0.98 | 0.49 | 0.86 |
| *N. spurca* | 2 | GBM | 0.61 | 0.89 | 0.63 | 0.61 |
| *N. spurca* | 2 | MAXENT | 0.27 | 0.84 | 0.26 | 0.57 |
| *N. spurca* | 2 | RF | 0.48 | 0.97 | 0.48 | 0.82 |
| *N. spurca* | 3 | GBM | 0.57 | 0.89 | 0.57 | 0.59 |
| *N. spurca* | 3 | MAXENT | 0.32 | 0.84 | 0.31 | 0.57 |
| *N. spurca* | 3 | RF | 0.48 | 0.98 | 0.47 | 0.82 |
| *N. spurca* | 4 | GBM | 0.61 | 0.94 | 0.61 | 0.71 |
| *N. spurca* | 4 | MAXENT | 0.23 | 0.80 | 0.23 | 0.54 |
| *N. spurca* | 4 | RF | 0.53 | 0.99 | 0.53 | 0.89 |
| *N. spurca* | 5 | GBM | 0.63 | 0.84 | 0.62 | 0.50 |
| *N. spurca* | 5 | MAXENT | 0.28 | 0.82 | 0.28 | 0.55 |
| *N. spurca* | 5 | RF | 0.52 | 0.95 | 0.54 | 0.76 |
| *N. spurca* | 6 | GBM | 0.60 | 0.91 | 0.59 | 0.70 |
| *N. spurca* | 6 | MAXENT | 0.25 | 0.84 | 0.26 | 0.57 |
| *N. spurca* | 6 | RF | 0.48 | 0.98 | 0.49 | 0.84 |
| *N. spurca* | 7 | GBM | 0.59 | 0.91 | 0.63 | 0.70 |
| *N. spurca* | 7 | MAXENT | 0.30 | 0.85 | 0.31 | 0.60 |
| *N. spurca* | 7 | RF | 0.49 | 0.96 | 0.51 | 0.75 |
| *N. spurca* | 8 | GBM | 0.63 | 0.89 | 0.63 | 0.60 |
| *N. spurca* | 8 | MAXENT | 0.30 | 0.81 | 0.30 | 0.55 |
| *N. spurca* | 8 | RF | 0.52 | 0.96 | 0.53 | 0.76 |
| *N. spurca* | 9 | GBM | 0.62 | 0.88 | 0.61 | 0.57 |
| *N. spurca* | 9 | MAXENT | 0.31 | 0.82 | 0.30 | 0.58 |
| *N. spurca* | 9 | RF | 0.51 | 0.95 | 0.52 | 0.71 |
| *N. spurca* | 10 | GBM | 0.60 | 0.92 | 0.60 | 0.71 |
| *N. spurca* | 10 | MAXENT | 0.30 | 0.80 | 0.30 | 0.54 |
| *N. spurca* | 10 | RF | 0.50 | 0.97 | 0.51 | 0.81 |
| *N. spurca* | 11 | GBM | 0.60 | 0.90 | 0.61 | 0.61 |
| *N. spurca* | 11 | MAXENT | 0.30 | 0.83 | 0.29 | 0.56 |
| *N. spurca* | 11 | RF | 0.49 | 0.96 | 0.49 | 0.79 |
| *N. spurca* | 12 | GBM | 0.64 | 0.88 | 0.64 | 0.57 |
| *N. spurca* | 12 | MAXENT | 0.27 | 0.86 | 0.27 | 0.57 |
| *N. spurca* | 12 | RF | 0.51 | 0.95 | 0.53 | 0.72 |
| *N. spurca* | 13 | GBM | 0.60 | 0.88 | 0.61 | 0.61 |
| *N. spurca* | 13 | MAXENT | 0.28 | 0.82 | 0.28 | 0.55 |
| *N. spurca* | 13 | RF | 0.47 | 0.96 | 0.47 | 0.81 |
| *N. spurca* | 14 | GBM | 0.61 | 0.89 | 0.61 | 0.67 |
| *N. spurca* | 14 | MAXENT | 0.27 | 0.81 | 0.27 | 0.53 |
| *N. spurca* | 14 | RF | 0.50 | 0.96 | 0.51 | 0.80 |
| *N. spurca* | 15 | GBM | 0.58 | 0.91 | 0.60 | 0.63 |
| *N. spurca* | 15 | MAXENT | 0.26 | 0.82 | 0.26 | 0.61 |
| *N. spurca* | 15 | RF | 0.45 | 0.97 | 0.59 | 0.78 |
| *N. spurca* | 16 | GBM | 0.62 | 0.90 | 0.62 | 0.64 |
| *N. spurca* | 16 | MAXENT | 0.23 | 0.80 | 0.31 | 0.54 |
| *N. spurca* | 16 | RF | 0.47 | 0.98 | 0.47 | 0.82 |
| *N. spurca* | 17 | GBM | 0.63 | 0.89 | 0.62 | 0.60 |
| *N. spurca* | 17 | MAXENT | 0.27 | 0.79 | 0.27 | 0.53 |
| *N. spurca* | 17 | RF | 0.50 | 0.98 | 0.51 | 0.85 |
| *N. spurca* | 18 | GBM | 0.60 | 0.95 | 0.60 | 0.77 |
| *N. spurca* | 18 | MAXENT | 0.27 | 0.82 | 0.27 | 0.57 |
| *N. spurca* | 18 | RF | 0.54 | 0.99 | 0.54 | 0.88 |
| *N. spurca* | 19 | GBM | 0.62 | 0.87 | 0.62 | 0.60 |
| *N. spurca* | 19 | MAXENT | 0.25 | 0.82 | 0.24 | 0.55 |
| *N. spurca* | 19 | RF | 0.50 | 0.97 | 0.51 | 0.77 |
| *N. spurca* | 20 | GBM | 0.62 | 0.91 | 0.62 | 0.63 |
| *N. spurca* | 20 | MAXENT | 0.31 | 0.86 | 0.31 | 0.62 |
| *N. spurca* | 20 | RF | 0.54 | 0.96 | 0.55 | 0.77 |
| *N. spurca* | 21 | GBM | 0.62 | 0.92 | 0.62 | 0.71 |
| *N. spurca* | 21 | MAXENT | 0.27 | 0.81 | 0.27 | 0.53 |
| *N. spurca* | 21 | RF | 0.53 | 0.98 | 0.55 | 0.85 |
| *N. spurca* | 22 | GBM | 0.59 | 0.91 | 0.58 | 0.72 |
| *N. spurca* | 22 | MAXENT | 0.26 | 0.80 | 0.26 | 0.53 |
| *N. spurca* | 22 | RF | 0.53 | 0.97 | 0.54 | 0.84 |
| *N. spurca* | 23 | GBM | 0.62 | 0.91 | 0.62 | 0.66 |
| *N. spurca* | 23 | MAXENT | 0.28 | 0.84 | 0.28 | 0.59 |
| *N. spurca* | 23 | RF | 0.51 | 0.97 | 0.51 | 0.82 |
| *N. spurca* | 24 | GBM | 0.62 | 0.90 | 0.62 | 0.64 |
| *N. spurca* | 24 | MAXENT | 0.28 | 0.84 | 0.29 | 0.60 |
| *N. spurca* | 24 | RF | 0.52 | 0.97 | 0.52 | 0.81 |
| *N. spurca* | 25 | GBM | 0.56 | 0.87 | 0.56 | 0.58 |
| *N. spurca* | 25 | MAXENT | 0.24 | 0.83 | 0.23 | 0.59 |
| *N. spurca* | 25 | RF | 0.48 | 0.95 | 0.48 | 0.78 |
| *N. spurca* | 26 | GBM | 0.62 | 0.90 | 0.61 | 0.62 |
| *N. spurca* | 26 | MAXENT | 0.27 | 0.82 | 0.29 | 0.61 |
| *N. spurca* | 26 | RF | 0.48 | 0.98 | 0.48 | 0.82 |
| *N. spurca* | 27 | GBM | 0.62 | 0.90 | 0.61 | 0.68 |
| *N. spurca* | 27 | MAXENT | 0.28 | 0.81 | 0.27 | 0.55 |
| *N. spurca* | 27 | RF | 0.54 | 0.97 | 0.54 | 0.83 |
| *N. spurca* | 28 | GBM | 0.58 | 0.91 | 0.60 | 0.69 |
| *N. spurca* | 28 | MAXENT | 0.32 | 0.83 | 0.32 | 0.55 |
| *N. spurca* | 28 | RF | 0.52 | 0.97 | 0.52 | 0.81 |
| *N. spurca* | 29 | GBM | 0.57 | 0.86 | 0.57 | 0.53 |
| *N. spurca* | 29 | MAXENT | 0.30 | 0.81 | 0.30 | 0.55 |
| *N. spurca* | 29 | RF | 0.46 | 0.97 | 0.47 | 0.70 |
| *N. spurca* | 30 | GBM | 0.65 | 0.89 | 0.65 | 0.58 |
| *N. spurca* | 30 | MAXENT | 0.25 | 0.85 | 0.24 | 0.61 |
| *N. spurca* | 30 | RF | 0.51 | 0.98 | 0.53 | 0.82 |
| *N. spurca* | 31 | GBM | 0.62 | 0.90 | 0.62 | 0.64 |
| *N. spurca* | 31 | MAXENT | 0.25 | 0.81 | 0.25 | 0.53 |
| *N. spurca* | 31 | RF | 0.51 | 0.98 | 0.51 | 0.82 |
| *N. spurca* | 32 | GBM | 0.58 | 0.93 | 0.59 | 0.70 |
| *N. spurca* | 32 | MAXENT | 0.27 | 0.83 | 0.27 | 0.58 |
| *N. spurca* | 32 | RF | 0.51 | 0.97 | 0.51 | 0.83 |
| *N. spurca* | 33 | GBM | 0.60 | 0.88 | 0.60 | 0.59 |
| *N. spurca* | 33 | MAXENT | 0.27 | 0.86 | 0.26 | 0.60 |
| *N. spurca* | 33 | RF | 0.49 | 0.95 | 0.50 | 0.77 |
| *N. spurca* | 34 | GBM | 0.62 | 0.88 | 0.62 | 0.59 |
| *N. spurca* | 34 | MAXENT | 0.27 | 0.79 | 0.26 | 0.53 |
| *N. spurca* | 34 | RF | 0.50 | 0.95 | 0.51 | 0.79 |
| *N. spurca* | 35 | GBM | 0.63 | 0.91 | 0.63 | 0.66 |
| *N. spurca* | 35 | MAXENT | 0.27 | 0.83 | 0.26 | 0.60 |
| *N. spurca* | 35 | RF | 0.50 | 0.98 | 0.51 | 0.81 |
| *N. spurca* | 36 | GBM | 0.61 | 0.90 | 0.62 | 0.62 |
| *N. spurca* | 36 | MAXENT | 0.26 | 0.84 | 0.26 | 0.56 |
| *N. spurca* | 36 | RF | 0.47 | 0.97 | 0.47 | 0.77 |
| *N. spurca* | 37 | GBM | 0.58 | 0.88 | 0.57 | 0.62 |
| *N. spurca* | 37 | MAXENT | 0.24 | 0.83 | 0.23 | 0.59 |
| *N. spurca* | 37 | RF | 0.50 | 0.97 | 0.57 | 0.78 |
| *N. spurca* | 38 | GBM | 0.63 | 0.92 | 0.63 | 0.63 |
| *N. spurca* | 38 | MAXENT | 0.25 | 0.82 | 0.24 | 0.55 |
| *N. spurca* | 38 | RF | 0.49 | 0.97 | 0.49 | 0.80 |
| *N. spurca* | 39 | GBM | 0.61 | 0.90 | 0.61 | 0.62 |
| *N. spurca* | 39 | MAXENT | 0.26 | 0.82 | 0.26 | 0.58 |
| *N. spurca* | 39 | RF | 0.49 | 0.96 | 0.49 | 0.78 |
| *N. spurca* | 40 | GBM | 0.58 | 0.91 | 0.58 | 0.64 |
| *N. spurca* | 40 | MAXENT | 0.27 | 0.80 | 0.27 | 0.55 |
| *N. spurca* | 40 | RF | 0.54 | 0.97 | 0.54 | 0.82 |
| *N. spurca* | 41 | GBM | 0.61 | 0.88 | 0.60 | 0.63 |
| *N. spurca* | 41 | MAXENT | 0.30 | 0.81 | 0.30 | 0.57 |
| *N. spurca* | 41 | RF | 0.50 | 0.97 | 0.50 | 0.76 |
| *N. spurca* | 42 | GBM | 0.63 | 0.90 | 0.60 | 0.66 |
| *N. spurca* | 42 | MAXENT | 0.32 | 0.82 | 0.31 | 0.59 |
| *N. spurca* | 42 | RF | 0.50 | 0.98 | 0.53 | 0.81 |
| *N. spurca* | 43 | GBM | 0.59 | 0.92 | 0.59 | 0.72 |
| *N. spurca* | 43 | MAXENT | 0.27 | 0.81 | 0.29 | 0.54 |
| *N. spurca* | 43 | RF | 0.49 | 0.98 | 0.49 | 0.85 |
| *N. spurca* | 44 | GBM | 0.57 | 0.92 | 0.60 | 0.67 |
| *N. spurca* | 44 | MAXENT | 0.27 | 0.83 | 0.27 | 0.57 |
| *N. spurca* | 44 | RF | 0.57 | 0.97 | 0.57 | 0.81 |
| *N. spurca* | 45 | GBM | 0.61 | 0.90 | 0.61 | 0.62 |
| *N. spurca* | 45 | MAXENT | 0.28 | 0.85 | 0.27 | 0.57 |
| *N. spurca* | 45 | RF | 0.51 | 0.97 | 0.51 | 0.76 |
| *N. spurca* | 46 | GBM | 0.64 | 0.91 | 0.64 | 0.64 |
| *N. spurca* | 46 | MAXENT | 0.25 | 0.83 | 0.24 | 0.55 |
| *N. spurca* | 46 | RF | 0.49 | 0.97 | 0.49 | 0.83 |
| *N. spurca* | 47 | GBM | 0.58 | 0.88 | 0.58 | 0.56 |
| *N. spurca* | 47 | MAXENT | 0.25 | 0.80 | 0.25 | 0.52 |
| *N. spurca* | 47 | RF | 0.48 | 0.97 | 0.48 | 0.76 |
| *N. spurca* | 48 | GBM | 0.61 | 0.90 | 0.60 | 0.58 |
| *N. spurca* | 48 | MAXENT | 0.30 | 0.83 | 0.29 | 0.60 |
| *N. spurca* | 48 | RF | 0.56 | 0.97 | 0.57 | 0.81 |
| *N. spurca* | 49 | GBM | 0.62 | 0.91 | 0.62 | 0.65 |
| *N. spurca* | 49 | MAXENT | 0.25 | 0.84 | 0.25 | 0.58 |
| *N. spurca* | 49 | RF | 0.50 | 0.97 | 0.52 | 0.84 |
| *N. spurca* | 50 | GBM | 0.63 | 0.87 | 0.63 | 0.52 |
| *N. spurca* | 50 | MAXENT | 0.25 | 0.84 | 0.25 | 0.55 |
| *N. spurca* | 50 | RF | 0.48 | 0.95 | 0.49 | 0.72 |
| *N. spurca* | 51 | GBM | 0.57 | 0.88 | 0.57 | 0.59 |
| *N. spurca* | 51 | MAXENT | 0.28 | 0.80 | 0.28 | 0.54 |
| *N. spurca* | 51 | RF | 0.54 | 0.97 | 0.54 | 0.81 |
| *N. spurca* | 52 | GBM | 0.63 | 0.90 | 0.61 | 0.67 |
| *N. spurca* | 52 | MAXENT | 0.21 | 0.82 | 0.30 | 0.56 |
| *N. spurca* | 52 | RF | 0.49 | 0.97 | 0.50 | 0.78 |
| *N. spurca* | 53 | GBM | 0.60 | 0.89 | 0.61 | 0.64 |
| *N. spurca* | 53 | MAXENT | 0.31 | 0.83 | 0.27 | 0.57 |
| *N. spurca* | 53 | RF | 0.51 | 0.96 | 0.51 | 0.76 |
| *N. spurca* | 54 | GBM | 0.57 | 0.89 | 0.57 | 0.67 |
| *N. spurca* | 54 | MAXENT | 0.24 | 0.78 | 0.24 | 0.51 |
| *N. spurca* | 54 | RF | 0.49 | 0.97 | 0.48 | 0.79 |
| *N. spurca* | 55 | GBM | 0.59 | 0.93 | 0.58 | 0.68 |
| *N. spurca* | 55 | MAXENT | 0.24 | 0.82 | 0.23 | 0.54 |
| *N. spurca* | 55 | RF | 0.50 | 0.98 | 0.51 | 0.75 |
| *N. spurca* | 56 | GBM | 0.62 | 0.91 | 0.62 | 0.66 |
| *N. spurca* | 56 | MAXENT | 0.27 | 0.82 | 0.27 | 0.56 |
| *N. spurca* | 56 | RF | 0.55 | 0.96 | 0.55 | 0.79 |
| *N. spurca* | 57 | GBM | 0.63 | 0.91 | 0.63 | 0.65 |
| *N. spurca* | 57 | MAXENT | 0.26 | 0.86 | 0.27 | 0.61 |
| *N. spurca* | 57 | RF | 0.47 | 0.97 | 0.50 | 0.84 |
| *N. spurca* | 58 | GBM | 0.62 | 0.91 | 0.61 | 0.66 |
| *N. spurca* | 58 | MAXENT | 0.25 | 0.83 | 0.25 | 0.58 |
| *N. spurca* | 58 | RF | 0.52 | 0.97 | 0.53 | 0.74 |
| *N. spurca* | 59 | GBM | 0.63 | 0.87 | 0.63 | 0.57 |
| *N. spurca* | 59 | MAXENT | 0.26 | 0.85 | 0.26 | 0.62 |
| *N. spurca* | 59 | RF | 0.52 | 0.95 | 0.53 | 0.75 |
| *N. spurca* | 60 | GBM | 0.60 | 0.90 | 0.60 | 0.59 |
| *N. spurca* | 60 | MAXENT | 0.31 | 0.83 | 0.32 | 0.57 |
| *N. spurca* | 60 | RF | 0.50 | 0.97 | 0.49 | 0.84 |
| *N. spurca* | 61 | GBM | 0.60 | 0.89 | 0.59 | 0.60 |
| *N. spurca* | 61 | MAXENT | 0.26 | 0.84 | 0.26 | 0.58 |
| *N. spurca* | 61 | RF | 0.52 | 0.95 | 0.53 | 0.77 |
| *N. spurca* | 62 | GBM | 0.62 | 0.87 | 0.62 | 0.54 |
| *N. spurca* | 62 | MAXENT | 0.29 | 0.86 | 0.29 | 0.59 |
| *N. spurca* | 62 | RF | 0.46 | 0.96 | 0.45 | 0.80 |
| *N. spurca* | 63 | GBM | 0.59 | 0.85 | 0.59 | 0.56 |
| *N. spurca* | 63 | MAXENT | 0.24 | 0.85 | 0.24 | 0.61 |
| *N. spurca* | 63 | RF | 0.52 | 0.97 | 0.52 | 0.79 |
| *N. spurca* | 64 | GBM | 0.60 | 0.91 | 0.60 | 0.65 |
| *N. spurca* | 64 | MAXENT | 0.28 | 0.86 | 0.28 | 0.58 |
| *N. spurca* | 64 | RF | 0.51 | 0.97 | 0.52 | 0.81 |
| *N. spurca* | 65 | GBM | 0.64 | 0.92 | 0.63 | 0.66 |
| *N. spurca* | 65 | MAXENT | 0.29 | 0.79 | 0.29 | 0.52 |
| *N. spurca* | 65 | RF | 0.57 | 0.98 | 0.52 | 0.86 |
| *N. spurca* | 66 | GBM | 0.60 | 0.90 | 0.60 | 0.63 |
| *N. spurca* | 66 | MAXENT | 0.23 | 0.81 | 0.23 | 0.54 |
| *N. spurca* | 66 | RF | 0.51 | 0.97 | 0.51 | 0.76 |
| *N. spurca* | 67 | GBM | 0.62 | 0.89 | 0.61 | 0.63 |
| *N. spurca* | 67 | MAXENT | 0.26 | 0.85 | 0.28 | 0.60 |
| *N. spurca* | 67 | RF | 0.48 | 0.97 | 0.48 | 0.81 |
| *N. spurca* | 68 | GBM | 0.62 | 0.92 | 0.62 | 0.66 |
| *N. spurca* | 68 | MAXENT | 0.31 | 0.80 | 0.31 | 0.55 |
| *N. spurca* | 68 | RF | 0.50 | 0.97 | 0.50 | 0.83 |
| *N. spurca* | 69 | GBM | 0.62 | 0.93 | 0.63 | 0.72 |
| *N. spurca* | 69 | MAXENT | 0.23 | 0.81 | 0.36 | 0.53 |
| *N. spurca* | 69 | RF | 0.48 | 0.98 | 0.47 | 0.83 |
| *N. spurca* | 70 | GBM | 0.63 | 0.88 | 0.62 | 0.63 |
| *N. spurca* | 70 | MAXENT | 0.29 | 0.80 | 0.29 | 0.51 |
| *N. spurca* | 70 | RF | 0.54 | 0.96 | 0.55 | 0.78 |
| *N. spurca* | 71 | GBM | 0.62 | 0.90 | 0.62 | 0.61 |
| *N. spurca* | 71 | MAXENT | 0.29 | 0.80 | 0.30 | 0.53 |
| *N. spurca* | 71 | RF | 0.50 | 0.97 | 0.51 | 0.76 |
| *N. spurca* | 72 | GBM | 0.58 | 0.91 | 0.57 | 0.66 |
| *N. spurca* | 72 | MAXENT | 0.25 | 0.84 | 0.33 | 0.59 |
| *N. spurca* | 72 | RF | 0.52 | 0.97 | 0.53 | 0.85 |
| *N. spurca* | 73 | GBM | 0.63 | 0.93 | 0.62 | 0.69 |
| *N. spurca* | 73 | MAXENT | 0.21 | 0.85 | 0.21 | 0.59 |
| *N. spurca* | 73 | RF | 0.54 | 0.98 | 0.54 | 0.88 |
| *N. spurca* | 74 | GBM | 0.63 | 0.88 | 0.62 | 0.56 |
| *N. spurca* | 74 | MAXENT | 0.26 | 0.81 | 0.28 | 0.54 |
| *N. spurca* | 74 | RF | 0.47 | 0.94 | 0.47 | 0.70 |
| *N. spurca* | 75 | GBM | 0.61 | 0.88 | 0.61 | 0.60 |
| *N. spurca* | 75 | MAXENT | 0.27 | 0.84 | 0.27 | 0.60 |
| *N. spurca* | 75 | RF | 0.49 | 0.97 | 0.48 | 0.82 |
| *N. spurca* | 76 | GBM | 0.59 | 0.89 | 0.60 | 0.61 |
| *N. spurca* | 76 | MAXENT | 0.29 | 0.83 | 0.29 | 0.59 |
| *N. spurca* | 76 | RF | 0.49 | 0.96 | 0.48 | 0.72 |
| *N. spurca* | 77 | GBM | 0.60 | 0.90 | 0.61 | 0.64 |
| *N. spurca* | 77 | MAXENT | 0.30 | 0.82 | 0.30 | 0.54 |
| *N. spurca* | 77 | RF | 0.50 | 0.97 | 0.52 | 0.80 |
| *N. spurca* | 78 | GBM | 0.62 | 0.91 | 0.61 | 0.63 |
| *N. spurca* | 78 | MAXENT | 0.28 | 0.80 | 0.27 | 0.55 |
| *N. spurca* | 78 | RF | 0.49 | 0.99 | 0.48 | 0.88 |
| *N. spurca* | 79 | GBM | 0.61 | 0.90 | 0.60 | 0.68 |
| *N. spurca* | 79 | MAXENT | 0.28 | 0.82 | 0.27 | 0.56 |
| *N. spurca* | 79 | RF | 0.51 | 0.98 | 0.53 | 0.83 |
| *N. spurca* | 80 | GBM | 0.62 | 0.87 | 0.62 | 0.60 |
| *N. spurca* | 80 | MAXENT | 0.24 | 0.82 | 0.24 | 0.57 |
| *N. spurca* | 80 | RF | 0.45 | 0.95 | 0.46 | 0.76 |
| *N. spurca* | 81 | GBM | 0.60 | 0.91 | 0.60 | 0.65 |
| *N. spurca* | 81 | MAXENT | 0.30 | 0.80 | 0.29 | 0.55 |
| *N. spurca* | 81 | RF | 0.50 | 0.98 | 0.49 | 0.81 |
| *N. spurca* | 82 | GBM | 0.60 | 0.90 | 0.61 | 0.68 |
| *N. spurca* | 82 | MAXENT | 0.32 | 0.85 | 0.31 | 0.60 |
| *N. spurca* | 82 | RF | 0.52 | 0.98 | 0.52 | 0.84 |
| *N. spurca* | 83 | GBM | 0.62 | 0.90 | 0.63 | 0.61 |
| *N. spurca* | 83 | MAXENT | 0.26 | 0.80 | 0.26 | 0.52 |
| *N. spurca* | 83 | RF | 0.54 | 0.98 | 0.55 | 0.86 |
| *N. spurca* | 84 | GBM | 0.62 | 0.92 | 0.62 | 0.67 |
| *N. spurca* | 84 | MAXENT | 0.28 | 0.80 | 0.28 | 0.55 |
| *N. spurca* | 84 | RF | 0.56 | 0.97 | 0.56 | 0.81 |
| *N. spurca* | 85 | GBM | 0.64 | 0.89 | 0.64 | 0.62 |
| *N. spurca* | 85 | MAXENT | 0.25 | 0.85 | 0.25 | 0.62 |
| *N. spurca* | 85 | RF | 0.51 | 0.96 | 0.52 | 0.76 |
| *N. spurca* | 86 | GBM | 0.62 | 0.90 | 0.63 | 0.60 |
| *N. spurca* | 86 | MAXENT | 0.29 | 0.81 | 0.27 | 0.55 |
| *N. spurca* | 86 | RF | 0.47 | 0.97 | 0.47 | 0.83 |
| *N. spurca* | 87 | GBM | 0.59 | 0.90 | 0.60 | 0.64 |
| *N. spurca* | 87 | MAXENT | 0.31 | 0.85 | 0.31 | 0.57 |
| *N. spurca* | 87 | RF | 0.49 | 0.97 | 0.48 | 0.83 |
| *N. spurca* | 88 | GBM | 0.63 | 0.89 | 0.62 | 0.58 |
| *N. spurca* | 88 | MAXENT | 0.26 | 0.84 | 0.25 | 0.58 |
| *N. spurca* | 88 | RF | 0.53 | 0.96 | 0.54 | 0.81 |
| *N. spurca* | 89 | GBM | 0.60 | 0.90 | 0.60 | 0.61 |
| *N. spurca* | 89 | MAXENT | 0.23 | 0.82 | 0.23 | 0.51 |
| *N. spurca* | 89 | RF | 0.47 | 0.97 | 0.49 | 0.77 |
| *N. spurca* | 90 | GBM | 0.63 | 0.89 | 0.63 | 0.57 |
| *N. spurca* | 90 | MAXENT | 0.28 | 0.83 | 0.27 | 0.57 |
| *N. spurca* | 90 | RF | 0.49 | 0.97 | 0.47 | 0.76 |
| *N. spurca* | 91 | GBM | 0.58 | 0.91 | 0.58 | 0.68 |
| *N. spurca* | 91 | MAXENT | 0.33 | 0.85 | 0.32 | 0.58 |
| *N. spurca* | 91 | RF | 0.48 | 0.97 | 0.48 | 0.80 |
| *N. spurca* | 92 | GBM | 0.59 | 0.91 | 0.59 | 0.61 |
| *N. spurca* | 92 | MAXENT | 0.28 | 0.80 | 0.27 | 0.55 |
| *N. spurca* | 92 | RF | 0.48 | 0.98 | 0.49 | 0.82 |
| *N. spurca* | 93 | GBM | 0.58 | 0.93 | 0.60 | 0.71 |
| *N. spurca* | 93 | MAXENT | 0.31 | 0.81 | 0.31 | 0.57 |
| *N. spurca* | 93 | RF | 0.49 | 0.98 | 0.52 | 0.87 |
| *N. spurca* | 94 | GBM | 0.63 | 0.87 | 0.63 | 0.59 |
| *N. spurca* | 94 | MAXENT | 0.28 | 0.83 | 0.28 | 0.58 |
| *N. spurca* | 94 | RF | 0.52 | 0.96 | 0.50 | 0.77 |
| *N. spurca* | 95 | GBM | 0.64 | 0.91 | 0.62 | 0.64 |
| *N. spurca* | 95 | MAXENT | 0.25 | 0.81 | 0.25 | 0.53 |
| *N. spurca* | 95 | RF | 0.49 | 0.97 | 0.53 | 0.84 |
| *N. spurca* | 96 | GBM | 0.63 | 0.90 | 0.62 | 0.66 |
| *N. spurca* | 96 | MAXENT | 0.24 | 0.83 | 0.24 | 0.56 |
| *N. spurca* | 96 | RF | 0.48 | 0.98 | 0.48 | 0.85 |
| *N. spurca* | 97 | GBM | 0.63 | 0.89 | 0.63 | 0.68 |
| *N. spurca* | 97 | MAXENT | 0.28 | 0.86 | 0.28 | 0.63 |
| *N. spurca* | 97 | RF | 0.49 | 0.97 | 0.49 | 0.79 |
| *N. spurca* | 98 | GBM | 0.62 | 0.88 | 0.62 | 0.62 |
| *N. spurca* | 98 | MAXENT | 0.27 | 0.83 | 0.27 | 0.57 |
| *N. spurca* | 98 | RF | 0.51 | 0.97 | 0.51 | 0.81 |
| *N. spurca* | 99 | GBM | 0.60 | 0.91 | 0.57 | 0.66 |
| *N. spurca* | 99 | MAXENT | 0.32 | 0.81 | 0.31 | 0.58 |
| *N. spurca* | 99 | RF | 0.48 | 0.96 | 0.48 | 0.77 |
| *N. spurca* | 100 | GBM | 0.62 | 0.87 | 0.62 | 0.57 |
| *N. spurca* | 100 | MAXENT | 0.28 | 0.80 | 0.27 | 0.54 |
| *N. spurca* | 100 | RF | 0.55 | 0.94 | 0.55 | 0.71 |
| *T. scrobilator* | 1 | GBM | 0.50 | 0.90 | 0.50 | 0.65 |
| *T. scrobilator* | 1 | MAXENT | 0.37 | 0.76 | 0.37 | 0.48 |
| *T. scrobilator* | 1 | RF | 0.49 | 0.96 | 0.49 | 0.79 |
| *T. scrobilator* | 2 | GBM | 0.55 | 0.89 | 0.55 | 0.67 |
| *T. scrobilator* | 2 | MAXENT | 0.39 | 0.81 | 0.38 | 0.55 |
| *T. scrobilator* | 2 | RF | 0.52 | 0.96 | 0.56 | 0.71 |
| *T. scrobilator* | 3 | GBM | 0.56 | 0.86 | 0.56 | 0.56 |
| *T. scrobilator* | 3 | MAXENT | 0.40 | 0.78 | 0.38 | 0.49 |
| *T. scrobilator* | 3 | RF | 0.49 | 0.94 | 0.49 | 0.75 |
| *T. scrobilator* | 4 | GBM | 0.48 | 0.84 | 0.49 | 0.62 |
| *T. scrobilator* | 4 | MAXENT | 0.39 | 0.80 | 0.38 | 0.41 |
| *T. scrobilator* | 4 | RF | 0.51 | 0.96 | 0.52 | 0.77 |
| *T. scrobilator* | 5 | GBM | 0.48 | 0.88 | 0.48 | 0.61 |
| *T. scrobilator* | 5 | MAXENT | 0.36 | 0.79 | 0.38 | 0.48 |
| *T. scrobilator* | 5 | RF | 0.56 | 0.95 | 0.56 | 0.80 |
| *T. scrobilator* | 6 | GBM | 0.48 | 0.87 | 0.48 | 0.60 |
| *T. scrobilator* | 6 | MAXENT | 0.38 | 0.78 | 0.37 | 0.49 |
| *T. scrobilator* | 6 | RF | 0.53 | 0.94 | 0.53 | 0.77 |
| *T. scrobilator* | 7 | GBM | 0.59 | 0.89 | 0.58 | 0.62 |
| *T. scrobilator* | 7 | MAXENT | 0.41 | 0.80 | 0.40 | 0.52 |
| *T. scrobilator* | 7 | RF | 0.53 | 0.94 | 0.53 | 0.71 |
| *T. scrobilator* | 8 | GBM | 0.50 | 0.88 | 0.50 | 0.64 |
| *T. scrobilator* | 8 | MAXENT | 0.39 | 0.82 | 0.37 | 0.47 |
| *T. scrobilator* | 8 | RF | 0.54 | 0.92 | 0.54 | 0.65 |
| *T. scrobilator* | 9 | GBM | 0.58 | 0.88 | 0.58 | 0.61 |
| *T. scrobilator* | 9 | MAXENT | 0.40 | 0.83 | 0.40 | 0.54 |
| *T. scrobilator* | 9 | RF | 0.51 | 0.95 | 0.52 | 0.73 |
| *T. scrobilator* | 10 | GBM | 0.47 | 0.81 | 0.47 | 0.42 |
| *T. scrobilator* | 10 | MAXENT | 0.39 | 0.77 | 0.37 | 0.44 |
| *T. scrobilator* | 10 | RF | 0.50 | 0.89 | 0.51 | 0.62 |
| *T. scrobilator* | 11 | GBM | 0.54 | 0.91 | 0.55 | 0.67 |
| *T. scrobilator* | 11 | MAXENT | 0.40 | 0.78 | 0.40 | 0.46 |
| *T. scrobilator* | 11 | RF | 0.51 | 0.98 | 0.51 | 0.85 |
| *T. scrobilator* | 12 | GBM | 0.45 | 0.90 | 0.45 | 0.61 |
| *T. scrobilator* | 12 | MAXENT | 0.40 | 0.80 | 0.40 | 0.54 |
| *T. scrobilator* | 12 | RF | 0.52 | 0.93 | 0.53 | 0.71 |
| *T. scrobilator* | 13 | GBM | 0.47 | 0.85 | 0.50 | 0.58 |
| *T. scrobilator* | 13 | MAXENT | 0.39 | 0.81 | 0.39 | 0.51 |
| *T. scrobilator* | 13 | RF | 0.53 | 0.94 | 0.53 | 0.73 |
| *T. scrobilator* | 14 | GBM | 0.47 | 0.86 | 0.48 | 0.58 |
| *T. scrobilator* | 14 | MAXENT | 0.36 | 0.76 | 0.36 | 0.46 |
| *T. scrobilator* | 14 | RF | 0.51 | 0.94 | 0.50 | 0.77 |
| *T. scrobilator* | 15 | GBM | 0.48 | 0.88 | 0.47 | 0.55 |
| *T. scrobilator* | 15 | MAXENT | 0.37 | 0.75 | 0.37 | 0.43 |
| *T. scrobilator* | 15 | RF | 0.52 | 0.93 | 0.54 | 0.65 |
| *T. scrobilator* | 16 | GBM | 0.53 | 0.87 | 0.53 | 0.56 |
| *T. scrobilator* | 16 | MAXENT | 0.39 | 0.79 | 0.38 | 0.49 |
| *T. scrobilator* | 16 | RF | 0.52 | 0.93 | 0.54 | 0.64 |
| *T. scrobilator* | 17 | GBM | 0.47 | 0.87 | 0.47 | 0.58 |
| *T. scrobilator* | 17 | MAXENT | 0.39 | 0.81 | 0.38 | 0.52 |
| *T. scrobilator* | 17 | RF | 0.55 | 0.93 | 0.55 | 0.69 |
| *T. scrobilator* | 18 | GBM | 0.55 | 0.86 | 0.54 | 0.56 |
| *T. scrobilator* | 18 | MAXENT | 0.39 | 0.85 | 0.41 | 0.51 |
| *T. scrobilator* | 18 | RF | 0.51 | 0.96 | 0.52 | 0.77 |
| *T. scrobilator* | 19 | GBM | 0.47 | 0.90 | 0.47 | 0.61 |
| *T. scrobilator* | 19 | MAXENT | 0.40 | 0.79 | 0.40 | 0.46 |
| *T. scrobilator* | 19 | RF | 0.55 | 0.95 | 0.54 | 0.80 |
| *T. scrobilator* | 20 | GBM | 0.56 | 0.84 | 0.56 | 0.60 |
| *T. scrobilator* | 20 | MAXENT | 0.40 | 0.79 | 0.40 | 0.54 |
| *T. scrobilator* | 20 | RF | 0.52 | 0.94 | 0.53 | 0.73 |
| *T. scrobilator* | 21 | GBM | 0.54 | 0.87 | 0.50 | 0.62 |
| *T. scrobilator* | 21 | MAXENT | 0.38 | 0.78 | 0.40 | 0.43 |
| *T. scrobilator* | 21 | RF | 0.52 | 0.96 | 0.54 | 0.79 |
| *T. scrobilator* | 22 | GBM | 0.46 | 0.86 | 0.46 | 0.65 |
| *T. scrobilator* | 22 | MAXENT | 0.40 | 0.77 | 0.40 | 0.45 |
| *T. scrobilator* | 22 | RF | 0.52 | 0.92 | 0.53 | 0.67 |
| *T. scrobilator* | 23 | GBM | 0.48 | 0.86 | 0.47 | 0.58 |
| *T. scrobilator* | 23 | MAXENT | 0.38 | 0.80 | 0.38 | 0.52 |
| *T. scrobilator* | 23 | RF | 0.48 | 0.94 | 0.48 | 0.75 |
| *T. scrobilator* | 24 | GBM | 0.48 | 0.88 | 0.47 | 0.60 |
| *T. scrobilator* | 24 | MAXENT | 0.39 | 0.78 | 0.39 | 0.43 |
| *T. scrobilator* | 24 | RF | 0.48 | 0.95 | 0.48 | 0.75 |
| *T. scrobilator* | 25 | GBM | 0.52 | 0.87 | 0.54 | 0.63 |
| *T. scrobilator* | 25 | MAXENT | 0.37 | 0.83 | 0.37 | 0.52 |
| *T. scrobilator* | 25 | RF | 0.53 | 0.93 | 0.54 | 0.77 |
| *T. scrobilator* | 26 | GBM | 0.47 | 0.90 | 0.48 | 0.62 |
| *T. scrobilator* | 26 | MAXENT | 0.36 | 0.79 | 0.36 | 0.49 |
| *T. scrobilator* | 26 | RF | 0.53 | 0.94 | 0.53 | 0.71 |
| *T. scrobilator* | 27 | GBM | 0.49 | 0.88 | 0.49 | 0.52 |
| *T. scrobilator* | 27 | MAXENT | 0.38 | 0.83 | 0.39 | 0.51 |
| *T. scrobilator* | 27 | RF | 0.55 | 0.93 | 0.55 | 0.73 |
| *T. scrobilator* | 28 | GBM | 0.53 | 0.84 | 0.53 | 0.54 |
| *T. scrobilator* | 28 | MAXENT | 0.38 | 0.81 | 0.38 | 0.53 |
| *T. scrobilator* | 28 | RF | 0.52 | 0.94 | 0.54 | 0.69 |
| *T. scrobilator* | 29 | GBM | 0.55 | 0.91 | 0.55 | 0.65 |
| *T. scrobilator* | 29 | MAXENT | 0.38 | 0.80 | 0.38 | 0.49 |
| *T. scrobilator* | 29 | RF | 0.51 | 0.96 | 0.51 | 0.80 |
| *T. scrobilator* | 30 | GBM | 0.55 | 0.89 | 0.54 | 0.62 |
| *T. scrobilator* | 30 | MAXENT | 0.40 | 0.77 | 0.39 | 0.48 |
| *T. scrobilator* | 30 | RF | 0.52 | 0.94 | 0.52 | 0.73 |
| *T. scrobilator* | 31 | GBM | 0.51 | 0.86 | 0.52 | 0.65 |
| *T. scrobilator* | 31 | MAXENT | 0.39 | 0.78 | 0.40 | 0.41 |
| *T. scrobilator* | 31 | RF | 0.51 | 0.94 | 0.53 | 0.77 |
| *T. scrobilator* | 32 | GBM | 0.52 | 0.92 | 0.46 | 0.73 |
| *T. scrobilator* | 32 | MAXENT | 0.42 | 0.81 | 0.39 | 0.52 |
| *T. scrobilator* | 32 | RF | 0.53 | 0.95 | 0.53 | 0.77 |
| *T. scrobilator* | 33 | GBM | 0.56 | 0.84 | 0.53 | 0.42 |
| *T. scrobilator* | 33 | MAXENT | 0.37 | 0.83 | 0.39 | 0.53 |
| *T. scrobilator* | 33 | RF | 0.52 | 0.94 | 0.54 | 0.73 |
| *T. scrobilator* | 34 | GBM | 0.48 | 0.87 | 0.49 | 0.64 |
| *T. scrobilator* | 34 | MAXENT | 0.38 | 0.79 | 0.38 | 0.53 |
| *T. scrobilator* | 34 | RF | 0.53 | 0.95 | 0.54 | 0.69 |
| *T. scrobilator* | 35 | GBM | 0.53 | 0.93 | 0.52 | 0.71 |
| *T. scrobilator* | 35 | MAXENT | 0.38 | 0.80 | 0.39 | 0.49 |
| *T. scrobilator* | 35 | RF | 0.49 | 0.94 | 0.51 | 0.69 |
| *T. scrobilator* | 36 | GBM | 0.48 | 0.87 | 0.48 | 0.58 |
| *T. scrobilator* | 36 | MAXENT | 0.39 | 0.82 | 0.38 | 0.54 |
| *T. scrobilator* | 36 | RF | 0.53 | 0.94 | 0.55 | 0.73 |
| *T. scrobilator* | 37 | GBM | 0.52 | 0.92 | 0.49 | 0.64 |
| *T. scrobilator* | 37 | MAXENT | 0.36 | 0.73 | 0.37 | 0.45 |
| *T. scrobilator* | 37 | RF | 0.49 | 0.97 | 0.48 | 0.79 |
| *T. scrobilator* | 38 | GBM | 0.51 | 0.81 | 0.52 | 0.44 |
| *T. scrobilator* | 38 | MAXENT | 0.40 | 0.81 | 0.40 | 0.54 |
| *T. scrobilator* | 38 | RF | 0.53 | 0.89 | 0.53 | 0.60 |
| *T. scrobilator* | 39 | GBM | 0.44 | 0.83 | 0.43 | 0.55 |
| *T. scrobilator* | 39 | MAXENT | 0.39 | 0.79 | 0.39 | 0.45 |
| *T. scrobilator* | 39 | RF | 0.50 | 0.92 | 0.53 | 0.75 |
| *T. scrobilator* | 40 | GBM | 0.46 | 0.86 | 0.46 | 0.62 |
| *T. scrobilator* | 40 | MAXENT | 0.40 | 0.80 | 0.39 | 0.48 |
| *T. scrobilator* | 40 | RF | 0.48 | 0.97 | 0.48 | 0.77 |
| *T. scrobilator* | 41 | GBM | 0.44 | 0.85 | 0.45 | 0.52 |
| *T. scrobilator* | 41 | MAXENT | 0.37 | 0.81 | 0.39 | 0.49 |
| *T. scrobilator* | 41 | RF | 0.47 | 0.92 | 0.53 | 0.71 |
| *T. scrobilator* | 42 | GBM | 0.54 | 0.90 | 0.55 | 0.63 |
| *T. scrobilator* | 42 | MAXENT | 0.38 | 0.82 | 0.38 | 0.53 |
| *T. scrobilator* | 42 | RF | 0.55 | 0.96 | 0.55 | 0.75 |
| *T. scrobilator* | 43 | GBM | 0.48 | 0.85 | 0.48 | 0.58 |
| *T. scrobilator* | 43 | MAXENT | 0.36 | 0.82 | 0.36 | 0.52 |
| *T. scrobilator* | 43 | RF | 0.53 | 0.93 | 0.53 | 0.65 |
| *T. scrobilator* | 44 | GBM | 0.48 | 0.88 | 0.48 | 0.60 |
| *T. scrobilator* | 44 | MAXENT | 0.38 | 0.77 | 0.41 | 0.45 |
| *T. scrobilator* | 44 | RF | 0.53 | 0.95 | 0.53 | 0.77 |
| *T. scrobilator* | 45 | GBM | 0.53 | 0.89 | 0.53 | 0.59 |
| *T. scrobilator* | 45 | MAXENT | 0.40 | 0.81 | 0.40 | 0.51 |
| *T. scrobilator* | 45 | RF | 0.52 | 0.95 | 0.52 | 0.77 |
| *T. scrobilator* | 46 | GBM | 0.49 | 0.86 | 0.49 | 0.65 |
| *T. scrobilator* | 46 | MAXENT | 0.38 | 0.77 | 0.38 | 0.51 |
| *T. scrobilator* | 46 | RF | 0.55 | 0.94 | 0.53 | 0.69 |
| *T. scrobilator* | 47 | GBM | 0.55 | 0.87 | 0.54 | 0.60 |
| *T. scrobilator* | 47 | MAXENT | 0.38 | 0.82 | 0.38 | 0.48 |
| *T. scrobilator* | 47 | RF | 0.51 | 0.93 | 0.53 | 0.64 |
| *T. scrobilator* | 48 | GBM | 0.49 | 0.82 | 0.50 | 0.62 |
| *T. scrobilator* | 48 | MAXENT | 0.40 | 0.79 | 0.41 | 0.46 |
| *T. scrobilator* | 48 | RF | 0.52 | 0.93 | 0.53 | 0.75 |
| *T. scrobilator* | 49 | GBM | 0.44 | 0.86 | 0.47 | 0.61 |
| *T. scrobilator* | 49 | MAXENT | 0.38 | 0.76 | 0.38 | 0.50 |
| *T. scrobilator* | 49 | RF | 0.55 | 0.93 | 0.55 | 0.75 |
| *T. scrobilator* | 50 | GBM | 0.52 | 0.92 | 0.52 | 0.69 |
| *T. scrobilator* | 50 | MAXENT | 0.38 | 0.79 | 0.38 | 0.52 |
| *T. scrobilator* | 50 | RF | 0.46 | 0.97 | 0.55 | 0.79 |
| *T. scrobilator* | 51 | GBM | 0.48 | 0.93 | 0.48 | 0.65 |
| *T. scrobilator* | 51 | MAXENT | 0.36 | 0.76 | 0.36 | 0.42 |
| *T. scrobilator* | 51 | RF | 0.53 | 0.97 | 0.54 | 0.77 |
| *T. scrobilator* | 52 | GBM | 0.51 | 0.89 | 0.51 | 0.69 |
| *T. scrobilator* | 52 | MAXENT | 0.40 | 0.80 | 0.39 | 0.50 |
| *T. scrobilator* | 52 | RF | 0.56 | 0.96 | 0.56 | 0.77 |
| *T. scrobilator* | 53 | GBM | 0.53 | 0.88 | 0.54 | 0.54 |
| *T. scrobilator* | 53 | MAXENT | 0.38 | 0.79 | 0.40 | 0.47 |
| *T. scrobilator* | 53 | RF | 0.53 | 0.94 | 0.55 | 0.67 |
| *T. scrobilator* | 54 | GBM | 0.54 | 0.85 | 0.54 | 0.58 |
| *T. scrobilator* | 54 | MAXENT | 0.39 | 0.81 | 0.39 | 0.50 |
| *T. scrobilator* | 54 | RF | 0.49 | 0.91 | 0.51 | 0.62 |
| *T. scrobilator* | 55 | GBM | 0.59 | 0.84 | 0.45 | 0.49 |
| *T. scrobilator* | 55 | MAXENT | 0.37 | 0.77 | 0.36 | 0.49 |
| *T. scrobilator* | 55 | RF | 0.49 | 0.90 | 0.49 | 0.61 |
| *T. scrobilator* | 56 | GBM | 0.59 | 0.91 | 0.60 | 0.67 |
| *T. scrobilator* | 56 | MAXENT | 0.40 | 0.79 | 0.40 | 0.45 |
| *T. scrobilator* | 56 | RF | 0.52 | 0.96 | 0.54 | 0.73 |
| *T. scrobilator* | 57 | GBM | 0.46 | 0.89 | 0.46 | 0.65 |
| *T. scrobilator* | 57 | MAXENT | 0.39 | 0.77 | 0.39 | 0.50 |
| *T. scrobilator* | 57 | RF | 0.53 | 0.97 | 0.55 | 0.81 |
| *T. scrobilator* | 58 | GBM | 0.53 | 0.85 | 0.53 | 0.56 |
| *T. scrobilator* | 58 | MAXENT | 0.38 | 0.82 | 0.38 | 0.53 |
| *T. scrobilator* | 58 | RF | 0.53 | 0.94 | 0.51 | 0.71 |
| *T. scrobilator* | 59 | GBM | 0.46 | 0.86 | 0.45 | 0.63 |
| *T. scrobilator* | 59 | MAXENT | 0.38 | 0.82 | 0.38 | 0.53 |
| *T. scrobilator* | 59 | RF | 0.53 | 0.95 | 0.54 | 0.80 |
| *T. scrobilator* | 60 | GBM | 0.46 | 0.78 | 0.51 | 0.50 |
| *T. scrobilator* | 60 | MAXENT | 0.40 | 0.83 | 0.40 | 0.55 |
| *T. scrobilator* | 60 | RF | 0.52 | 0.90 | 0.53 | 0.58 |
| *T. scrobilator* | 61 | GBM | 0.44 | 0.86 | 0.46 | 0.52 |
| *T. scrobilator* | 61 | MAXENT | 0.39 | 0.80 | 0.39 | 0.50 |
| *T. scrobilator* | 61 | RF | 0.54 | 0.91 | 0.54 | 0.65 |
| *T. scrobilator* | 62 | GBM | 0.49 | 0.84 | 0.50 | 0.55 |
| *T. scrobilator* | 62 | MAXENT | 0.39 | 0.78 | 0.41 | 0.41 |
| *T. scrobilator* | 62 | RF | 0.54 | 0.95 | 0.54 | 0.73 |
| *T. scrobilator* | 63 | GBM | 0.48 | 0.91 | 0.48 | 0.71 |
| *T. scrobilator* | 63 | MAXENT | 0.38 | 0.84 | 0.38 | 0.55 |
| *T. scrobilator* | 63 | RF | 0.52 | 0.96 | 0.53 | 0.79 |
| *T. scrobilator* | 64 | GBM | 0.50 | 0.83 | 0.50 | 0.58 |
| *T. scrobilator* | 64 | MAXENT | 0.39 | 0.81 | 0.41 | 0.49 |
| *T. scrobilator* | 64 | RF | 0.54 | 0.93 | 0.54 | 0.67 |
| *T. scrobilator* | 65 | GBM | 0.54 | 0.95 | 0.54 | 0.77 |
| *T. scrobilator* | 65 | MAXENT | 0.38 | 0.78 | 0.38 | 0.48 |
| *T. scrobilator* | 65 | RF | 0.51 | 0.97 | 0.54 | 0.77 |
| *T. scrobilator* | 66 | GBM | 0.49 | 0.88 | 0.49 | 0.60 |
| *T. scrobilator* | 66 | MAXENT | 0.40 | 0.76 | 0.40 | 0.39 |
| *T. scrobilator* | 66 | RF | 0.51 | 0.94 | 0.51 | 0.69 |
| *T. scrobilator* | 67 | GBM | 0.47 | 0.87 | 0.50 | 0.50 |
| *T. scrobilator* | 67 | MAXENT | 0.38 | 0.82 | 0.37 | 0.55 |
| *T. scrobilator* | 67 | RF | 0.53 | 0.94 | 0.53 | 0.73 |
| *T. scrobilator* | 68 | GBM | 0.47 | 0.83 | 0.48 | 0.56 |
| *T. scrobilator* | 68 | MAXENT | 0.37 | 0.75 | 0.37 | 0.47 |
| *T. scrobilator* | 68 | RF | 0.54 | 0.91 | 0.54 | 0.69 |
| *T. scrobilator* | 69 | GBM | 0.47 | 0.84 | 0.47 | 0.55 |
| *T. scrobilator* | 69 | MAXENT | 0.41 | 0.78 | 0.39 | 0.42 |
| *T. scrobilator* | 69 | RF | 0.54 | 0.91 | 0.55 | 0.63 |
| *T. scrobilator* | 70 | GBM | 0.51 | 0.90 | 0.55 | 0.62 |
| *T. scrobilator* | 70 | MAXENT | 0.38 | 0.80 | 0.39 | 0.53 |
| *T. scrobilator* | 70 | RF | 0.47 | 0.95 | 0.47 | 0.73 |
| *T. scrobilator* | 71 | GBM | 0.46 | 0.84 | 0.45 | 0.50 |
| *T. scrobilator* | 71 | MAXENT | 0.40 | 0.82 | 0.40 | 0.46 |
| *T. scrobilator* | 71 | RF | 0.51 | 0.93 | 0.52 | 0.67 |
| *T. scrobilator* | 72 | GBM | 0.52 | 0.85 | 0.52 | 0.53 |
| *T. scrobilator* | 72 | MAXENT | 0.38 | 0.78 | 0.39 | 0.49 |
| *T. scrobilator* | 72 | RF | 0.48 | 0.94 | 0.47 | 0.61 |
| *T. scrobilator* | 73 | GBM | 0.49 | 0.90 | 0.52 | 0.64 |
| *T. scrobilator* | 73 | MAXENT | 0.38 | 0.79 | 0.39 | 0.50 |
| *T. scrobilator* | 73 | RF | 0.54 | 0.96 | 0.54 | 0.73 |
| *T. scrobilator* | 74 | GBM | 0.43 | 0.87 | 0.43 | 0.71 |
| *T. scrobilator* | 74 | MAXENT | 0.38 | 0.77 | 0.37 | 0.47 |
| *T. scrobilator* | 74 | RF | 0.54 | 0.95 | 0.55 | 0.73 |
| *T. scrobilator* | 75 | GBM | 0.48 | 0.86 | 0.48 | 0.59 |
| *T. scrobilator* | 75 | MAXENT | 0.41 | 0.77 | 0.41 | 0.45 |
| *T. scrobilator* | 75 | RF | 0.50 | 0.94 | 0.51 | 0.75 |
| *T. scrobilator* | 76 | GBM | 0.50 | 0.91 | 0.50 | 0.71 |
| *T. scrobilator* | 76 | MAXENT | 0.39 | 0.80 | 0.39 | 0.48 |
| *T. scrobilator* | 76 | RF | 0.53 | 0.94 | 0.54 | 0.75 |
| *T. scrobilator* | 77 | GBM | 0.54 | 0.87 | 0.54 | 0.56 |
| *T. scrobilator* | 77 | MAXENT | 0.39 | 0.82 | 0.37 | 0.52 |
| *T. scrobilator* | 77 | RF | 0.51 | 0.93 | 0.54 | 0.75 |
| *T. scrobilator* | 78 | GBM | 0.50 | 0.90 | 0.49 | 0.60 |
| *T. scrobilator* | 78 | MAXENT | 0.38 | 0.82 | 0.38 | 0.50 |
| *T. scrobilator* | 78 | RF | 0.51 | 0.93 | 0.50 | 0.67 |
| *T. scrobilator* | 79 | GBM | 0.56 | 0.88 | 0.56 | 0.63 |
| *T. scrobilator* | 79 | MAXENT | 0.37 | 0.80 | 0.37 | 0.49 |
| *T. scrobilator* | 79 | RF | 0.54 | 0.96 | 0.54 | 0.77 |
| *T. scrobilator* | 80 | GBM | 0.52 | 0.88 | 0.53 | 0.64 |
| *T. scrobilator* | 80 | MAXENT | 0.39 | 0.77 | 0.39 | 0.51 |
| *T. scrobilator* | 80 | RF | 0.54 | 0.94 | 0.54 | 0.79 |
| *T. scrobilator* | 81 | GBM | 0.47 | 0.81 | 0.47 | 0.58 |
| *T. scrobilator* | 81 | MAXENT | 0.38 | 0.80 | 0.38 | 0.49 |
| *T. scrobilator* | 81 | RF | 0.53 | 0.96 | 0.54 | 0.73 |
| *T. scrobilator* | 82 | GBM | 0.49 | 0.85 | 0.49 | 0.65 |
| *T. scrobilator* | 82 | MAXENT | 0.38 | 0.82 | 0.38 | 0.54 |
| *T. scrobilator* | 82 | RF | 0.51 | 0.94 | 0.52 | 0.63 |
| *T. scrobilator* | 83 | GBM | 0.56 | 0.91 | 0.55 | 0.64 |
| *T. scrobilator* | 83 | MAXENT | 0.39 | 0.77 | 0.39 | 0.47 |
| *T. scrobilator* | 83 | RF | 0.49 | 0.94 | 0.48 | 0.69 |
| *T. scrobilator* | 84 | GBM | 0.52 | 0.84 | 0.54 | 0.56 |
| *T. scrobilator* | 84 | MAXENT | 0.38 | 0.79 | 0.38 | 0.51 |
| *T. scrobilator* | 84 | RF | 0.53 | 0.89 | 0.54 | 0.64 |
| *T. scrobilator* | 85 | GBM | 0.48 | 0.88 | 0.48 | 0.53 |
| *T. scrobilator* | 85 | MAXENT | 0.33 | 0.71 | 0.33 | 0.39 |
| *T. scrobilator* | 85 | RF | 0.53 | 0.93 | 0.54 | 0.67 |
| *T. scrobilator* | 86 | GBM | 0.47 | 0.90 | 0.47 | 0.64 |
| *T. scrobilator* | 86 | MAXENT | 0.39 | 0.83 | 0.37 | 0.49 |
| *T. scrobilator* | 86 | RF | 0.54 | 0.95 | 0.54 | 0.73 |
| *T. scrobilator* | 87 | GBM | 0.49 | 0.82 | 0.49 | 0.46 |
| *T. scrobilator* | 87 | MAXENT | 0.38 | 0.79 | 0.38 | 0.50 |
| *T. scrobilator* | 87 | RF | 0.48 | 0.93 | 0.56 | 0.69 |
| *T. scrobilator* | 88 | GBM | 0.48 | 0.91 | 0.48 | 0.77 |
| *T. scrobilator* | 88 | MAXENT | 0.40 | 0.79 | 0.40 | 0.50 |
| *T. scrobilator* | 88 | RF | 0.48 | 0.97 | 0.48 | 0.81 |
| *T. scrobilator* | 89 | GBM | 0.51 | 0.89 | 0.51 | 0.63 |
| *T. scrobilator* | 89 | MAXENT | 0.40 | 0.80 | 0.40 | 0.48 |
| *T. scrobilator* | 89 | RF | 0.53 | 0.94 | 0.54 | 0.69 |
| *T. scrobilator* | 90 | GBM | 0.54 | 0.91 | 0.54 | 0.64 |
| *T. scrobilator* | 90 | MAXENT | 0.40 | 0.83 | 0.40 | 0.52 |
| *T. scrobilator* | 90 | RF | 0.54 | 0.94 | 0.55 | 0.79 |
| *T. scrobilator* | 91 | GBM | 0.47 | 0.82 | 0.48 | 0.54 |
| *T. scrobilator* | 91 | MAXENT | 0.38 | 0.78 | 0.38 | 0.50 |
| *T. scrobilator* | 91 | RF | 0.55 | 0.91 | 0.55 | 0.67 |
| *T. scrobilator* | 92 | GBM | 0.46 | 0.86 | 0.46 | 0.61 |
| *T. scrobilator* | 92 | MAXENT | 0.36 | 0.80 | 0.36 | 0.48 |
| *T. scrobilator* | 92 | RF | 0.54 | 0.92 | 0.54 | 0.67 |
| *T. scrobilator* | 93 | GBM | 0.56 | 0.85 | 0.55 | 0.56 |
| *T. scrobilator* | 93 | MAXENT | 0.38 | 0.75 | 0.39 | 0.42 |
| *T. scrobilator* | 93 | RF | 0.55 | 0.91 | 0.54 | 0.64 |
| *T. scrobilator* | 94 | GBM | 0.49 | 0.92 | 0.50 | 0.73 |
| *T. scrobilator* | 94 | MAXENT | 0.39 | 0.81 | 0.39 | 0.51 |
| *T. scrobilator* | 94 | RF | 0.55 | 0.99 | 0.55 | 0.87 |
| *T. scrobilator* | 95 | GBM | 0.46 | 0.90 | 0.53 | 0.67 |
| *T. scrobilator* | 95 | MAXENT | 0.40 | 0.82 | 0.40 | 0.50 |
| *T. scrobilator* | 95 | RF | 0.52 | 0.98 | 0.52 | 0.82 |
| *T. scrobilator* | 96 | GBM | 0.51 | 0.82 | 0.54 | 0.44 |
| *T. scrobilator* | 96 | MAXENT | 0.41 | 0.77 | 0.40 | 0.48 |
| *T. scrobilator* | 96 | RF | 0.52 | 0.90 | 0.52 | 0.64 |
| *T. scrobilator* | 97 | GBM | 0.47 | 0.91 | 0.49 | 0.71 |
| *T. scrobilator* | 97 | MAXENT | 0.39 | 0.79 | 0.39 | 0.50 |
| *T. scrobilator* | 97 | RF | 0.49 | 0.97 | 0.48 | 0.87 |
| *T. scrobilator* | 98 | GBM | 0.49 | 0.87 | 0.48 | 0.58 |
| *T. scrobilator* | 98 | MAXENT | 0.38 | 0.81 | 0.38 | 0.52 |
| *T. scrobilator* | 98 | RF | 0.50 | 0.93 | 0.52 | 0.69 |
| *T. scrobilator* | 99 | GBM | 0.48 | 0.91 | 0.48 | 0.69 |
| *T. scrobilator* | 99 | MAXENT | 0.36 | 0.81 | 0.36 | 0.49 |
| *T. scrobilator* | 99 | RF | 0.52 | 0.94 | 0.53 | 0.71 |
| *T. scrobilator* | 100 | GBM | 0.49 | 0.85 | 0.49 | 0.62 |
| *T. scrobilator* | 100 | MAXENT | 0.38 | 0.81 | 0.38 | 0.54 |
| *T. scrobilator* | 100 | RF | 0.46 | 0.92 | 0.47 | 0.73 |
| *Z. pyrum* | 1 | GBM | 0.48 | 0.83 | 0.48 | 0.53 |
| *Z. pyrum* | 1 | MAXENT | 0.25 | 0.86 | 0.26 | 0.62 |
| *Z. pyrum* | 1 | RF | 0.51 | 0.92 | 0.51 | 0.63 |
| *Z. pyrum* | 2 | GBM | 0.51 | 0.91 | 0.51 | 0.63 |
| *Z. pyrum* | 2 | MAXENT | 0.25 | 0.86 | 0.27 | 0.57 |
| *Z. pyrum* | 2 | RF | 0.51 | 0.94 | 0.52 | 0.69 |
| *Z. pyrum* | 3 | GBM | 0.49 | 0.90 | 0.48 | 0.58 |
| *Z. pyrum* | 3 | MAXENT | 0.23 | 0.85 | 0.23 | 0.51 |
| *Z. pyrum* | 3 | RF | 0.49 | 0.93 | 0.48 | 0.67 |
| *Z. pyrum* | 4 | GBM | 0.50 | 0.94 | 0.49 | 0.77 |
| *Z. pyrum* | 4 | MAXENT | 0.24 | 0.85 | 0.24 | 0.57 |
| *Z. pyrum* | 4 | RF | 0.49 | 0.96 | 0.49 | 0.80 |
| *Z. pyrum* | 5 | GBM | 0.50 | 0.93 | 0.50 | 0.66 |
| *Z. pyrum* | 5 | MAXENT | 0.24 | 0.87 | 0.24 | 0.58 |
| *Z. pyrum* | 5 | RF | 0.55 | 0.95 | 0.55 | 0.69 |
| *Z. pyrum* | 6 | GBM | 0.51 | 0.89 | 0.52 | 0.61 |
| *Z. pyrum* | 6 | MAXENT | 0.29 | 0.81 | 0.28 | 0.45 |
| *Z. pyrum* | 6 | RF | 0.51 | 0.94 | 0.52 | 0.73 |
| *Z. pyrum* | 7 | GBM | 0.48 | 0.89 | 0.49 | 0.69 |
| *Z. pyrum* | 7 | MAXENT | 0.26 | 0.89 | 0.26 | 0.65 |
| *Z. pyrum* | 7 | RF | 0.52 | 0.95 | 0.53 | 0.79 |
| *Z. pyrum* | 8 | GBM | 0.48 | 0.87 | 0.49 | 0.61 |
| *Z. pyrum* | 8 | MAXENT | 0.25 | 0.81 | 0.24 | 0.51 |
| *Z. pyrum* | 8 | RF | 0.53 | 0.92 | 0.53 | 0.73 |
| *Z. pyrum* | 9 | GBM | 0.43 | 0.92 | 0.43 | 0.74 |
| *Z. pyrum* | 9 | MAXENT | 0.26 | 0.84 | 0.26 | 0.51 |
| *Z. pyrum* | 9 | RF | 0.50 | 0.95 | 0.51 | 0.77 |
| *Z. pyrum* | 10 | GBM | 0.51 | 0.90 | 0.51 | 0.69 |
| *Z. pyrum* | 10 | MAXENT | 0.29 | 0.88 | 0.26 | 0.61 |
| *Z. pyrum* | 10 | RF | 0.52 | 0.96 | 0.52 | 0.80 |
| *Z. pyrum* | 11 | GBM | 0.51 | 0.89 | 0.50 | 0.61 |
| *Z. pyrum* | 11 | MAXENT | 0.23 | 0.85 | 0.22 | 0.51 |
| *Z. pyrum* | 11 | RF | 0.50 | 0.93 | 0.51 | 0.70 |
| *Z. pyrum* | 12 | GBM | 0.50 | 0.94 | 0.51 | 0.75 |
| *Z. pyrum* | 12 | MAXENT | 0.28 | 0.84 | 0.27 | 0.51 |
| *Z. pyrum* | 12 | RF | 0.47 | 0.96 | 0.50 | 0.72 |
| *Z. pyrum* | 13 | GBM | 0.52 | 0.92 | 0.50 | 0.64 |
| *Z. pyrum* | 13 | MAXENT | 0.24 | 0.85 | 0.23 | 0.57 |
| *Z. pyrum* | 13 | RF | 0.53 | 0.93 | 0.53 | 0.72 |
| *Z. pyrum* | 14 | GBM | 0.42 | 0.92 | 0.43 | 0.77 |
| *Z. pyrum* | 14 | MAXENT | 0.30 | 0.84 | 0.29 | 0.49 |
| *Z. pyrum* | 14 | RF | 0.54 | 0.95 | 0.54 | 0.80 |
| *Z. pyrum* | 15 | GBM | 0.52 | 0.90 | 0.52 | 0.66 |
| *Z. pyrum* | 15 | MAXENT | 0.28 | 0.86 | 0.27 | 0.57 |
| *Z. pyrum* | 15 | RF | 0.49 | 0.93 | 0.52 | 0.70 |
| *Z. pyrum* | 16 | GBM | 0.52 | 0.92 | 0.52 | 0.70 |
| *Z. pyrum* | 16 | MAXENT | 0.25 | 0.87 | 0.23 | 0.64 |
| *Z. pyrum* | 16 | RF | 0.51 | 0.95 | 0.51 | 0.73 |
| *Z. pyrum* | 17 | GBM | 0.51 | 0.87 | 0.51 | 0.57 |
| *Z. pyrum* | 17 | MAXENT | 0.24 | 0.88 | 0.23 | 0.64 |
| *Z. pyrum* | 17 | RF | 0.51 | 0.94 | 0.52 | 0.68 |
| *Z. pyrum* | 18 | GBM | 0.49 | 0.87 | 0.49 | 0.59 |
| *Z. pyrum* | 18 | MAXENT | 0.26 | 0.85 | 0.38 | 0.47 |
| *Z. pyrum* | 18 | RF | 0.49 | 0.93 | 0.51 | 0.67 |
| *Z. pyrum* | 19 | GBM | 0.50 | 0.87 | 0.50 | 0.62 |
| *Z. pyrum* | 19 | MAXENT | 0.25 | 0.86 | 0.24 | 0.62 |
| *Z. pyrum* | 19 | RF | 0.52 | 0.93 | 0.52 | 0.65 |
| *Z. pyrum* | 20 | GBM | 0.49 | 0.88 | 0.49 | 0.61 |
| *Z. pyrum* | 20 | MAXENT | 0.25 | 0.85 | 0.24 | 0.57 |
| *Z. pyrum* | 20 | RF | 0.52 | 0.93 | 0.52 | 0.70 |
| *Z. pyrum* | 21 | GBM | 0.50 | 0.87 | 0.50 | 0.56 |
| *Z. pyrum* | 21 | MAXENT | 0.29 | 0.85 | 0.28 | 0.54 |
| *Z. pyrum* | 21 | RF | 0.51 | 0.93 | 0.51 | 0.72 |
| *Z. pyrum* | 22 | GBM | 0.51 | 0.95 | 0.51 | 0.72 |
| *Z. pyrum* | 22 | MAXENT | 0.25 | 0.85 | 0.24 | 0.58 |
| *Z. pyrum* | 22 | RF | 0.49 | 0.97 | 0.49 | 0.79 |
| *Z. pyrum* | 23 | GBM | 0.50 | 0.91 | 0.50 | 0.69 |
| *Z. pyrum* | 23 | MAXENT | 0.25 | 0.86 | 0.27 | 0.59 |
| *Z. pyrum* | 23 | RF | 0.52 | 0.92 | 0.53 | 0.66 |
| *Z. pyrum* | 24 | GBM | 0.51 | 0.90 | 0.51 | 0.66 |
| *Z. pyrum* | 24 | MAXENT | 0.26 | 0.89 | 0.26 | 0.67 |
| *Z. pyrum* | 24 | RF | 0.48 | 0.97 | 0.49 | 0.85 |
| *Z. pyrum* | 25 | GBM | 0.54 | 0.90 | 0.54 | 0.61 |
| *Z. pyrum* | 25 | MAXENT | 0.29 | 0.88 | 0.29 | 0.59 |
| *Z. pyrum* | 25 | RF | 0.53 | 0.94 | 0.54 | 0.72 |
| *Z. pyrum* | 26 | GBM | 0.54 | 0.90 | 0.54 | 0.64 |
| *Z. pyrum* | 26 | MAXENT | 0.24 | 0.88 | 0.23 | 0.61 |
| *Z. pyrum* | 26 | RF | 0.49 | 0.96 | 0.48 | 0.80 |
| *Z. pyrum* | 27 | GBM | 0.52 | 0.86 | 0.52 | 0.51 |
| *Z. pyrum* | 27 | MAXENT | 0.25 | 0.80 | 0.24 | 0.42 |
| *Z. pyrum* | 27 | RF | 0.51 | 0.91 | 0.52 | 0.60 |
| *Z. pyrum* | 28 | GBM | 0.49 | 0.93 | 0.49 | 0.63 |
| *Z. pyrum* | 28 | MAXENT | 0.27 | 0.84 | 0.27 | 0.58 |
| *Z. pyrum* | 28 | RF | 0.49 | 0.97 | 0.50 | 0.77 |
| *Z. pyrum* | 29 | GBM | 0.50 | 0.90 | 0.49 | 0.74 |
| *Z. pyrum* | 29 | MAXENT | 0.26 | 0.86 | 0.26 | 0.54 |
| *Z. pyrum* | 29 | RF | 0.54 | 0.95 | 0.54 | 0.75 |
| *Z. pyrum* | 30 | GBM | 0.48 | 0.87 | 0.48 | 0.61 |
| *Z. pyrum* | 30 | MAXENT | 0.25 | 0.84 | 0.26 | 0.57 |
| *Z. pyrum* | 30 | RF | 0.50 | 0.89 | 0.50 | 0.58 |
| *Z. pyrum* | 31 | GBM | 0.50 | 0.94 | 0.50 | 0.77 |
| *Z. pyrum* | 31 | MAXENT | 0.27 | 0.85 | 0.27 | 0.52 |
| *Z. pyrum* | 31 | RF | 0.52 | 0.97 | 0.53 | 0.84 |
| *Z. pyrum* | 32 | GBM | 0.51 | 0.88 | 0.51 | 0.62 |
| *Z. pyrum* | 32 | MAXENT | 0.28 | 0.83 | 0.28 | 0.52 |
| *Z. pyrum* | 32 | RF | 0.52 | 0.93 | 0.53 | 0.69 |
| *Z. pyrum* | 33 | GBM | 0.45 | 0.87 | 0.43 | 0.58 |
| *Z. pyrum* | 33 | MAXENT | 0.26 | 0.88 | 0.26 | 0.58 |
| *Z. pyrum* | 33 | RF | 0.52 | 0.93 | 0.52 | 0.69 |
| *Z. pyrum* | 34 | GBM | 0.53 | 0.91 | 0.54 | 0.62 |
| *Z. pyrum* | 34 | MAXENT | 0.25 | 0.85 | 0.25 | 0.55 |
| *Z. pyrum* | 34 | RF | 0.49 | 0.94 | 0.48 | 0.69 |
| *Z. pyrum* | 35 | GBM | 0.54 | 0.84 | 0.55 | 0.42 |
| *Z. pyrum* | 35 | MAXENT | 0.24 | 0.85 | 0.27 | 0.56 |
| *Z. pyrum* | 35 | RF | 0.48 | 0.90 | 0.51 | 0.56 |
| *Z. pyrum* | 36 | GBM | 0.47 | 0.90 | 0.47 | 0.67 |
| *Z. pyrum* | 36 | MAXENT | 0.24 | 0.86 | 0.24 | 0.57 |
| *Z. pyrum* | 36 | RF | 0.46 | 0.93 | 0.49 | 0.67 |
| *Z. pyrum* | 37 | GBM | 0.46 | 0.94 | 0.46 | 0.71 |
| *Z. pyrum* | 37 | MAXENT | 0.26 | 0.87 | 0.24 | 0.62 |
| *Z. pyrum* | 37 | RF | 0.52 | 0.97 | 0.52 | 0.77 |
| *Z. pyrum* | 38 | GBM | 0.51 | 0.87 | 0.51 | 0.56 |
| *Z. pyrum* | 38 | MAXENT | 0.27 | 0.88 | 0.25 | 0.62 |
| *Z. pyrum* | 38 | RF | 0.50 | 0.90 | 0.51 | 0.64 |
| *Z. pyrum* | 39 | GBM | 0.50 | 0.92 | 0.50 | 0.77 |
| *Z. pyrum* | 39 | MAXENT | 0.27 | 0.84 | 0.29 | 0.58 |
| *Z. pyrum* | 39 | RF | 0.53 | 0.95 | 0.54 | 0.77 |
| *Z. pyrum* | 40 | GBM | 0.53 | 0.90 | 0.54 | 0.61 |
| *Z. pyrum* | 40 | MAXENT | 0.24 | 0.84 | 0.24 | 0.53 |
| *Z. pyrum* | 40 | RF | 0.51 | 0.95 | 0.53 | 0.81 |
| *Z. pyrum* | 41 | GBM | 0.53 | 0.87 | 0.53 | 0.58 |
| *Z. pyrum* | 41 | MAXENT | 0.25 | 0.86 | 0.24 | 0.55 |
| *Z. pyrum* | 41 | RF | 0.54 | 0.93 | 0.54 | 0.64 |
| *Z. pyrum* | 42 | GBM | 0.51 | 0.92 | 0.51 | 0.68 |
| *Z. pyrum* | 42 | MAXENT | 0.29 | 0.87 | 0.28 | 0.58 |
| *Z. pyrum* | 42 | RF | 0.53 | 0.95 | 0.54 | 0.74 |
| *Z. pyrum* | 43 | GBM | 0.48 | 0.85 | 0.48 | 0.56 |
| *Z. pyrum* | 43 | MAXENT | 0.28 | 0.84 | 0.26 | 0.55 |
| *Z. pyrum* | 43 | RF | 0.52 | 0.92 | 0.52 | 0.66 |
| *Z. pyrum* | 44 | GBM | 0.50 | 0.89 | 0.50 | 0.65 |
| *Z. pyrum* | 44 | MAXENT | 0.25 | 0.87 | 0.28 | 0.60 |
| *Z. pyrum* | 44 | RF | 0.52 | 0.95 | 0.51 | 0.75 |
| *Z. pyrum* | 45 | GBM | 0.49 | 0.95 | 0.49 | 0.72 |
| *Z. pyrum* | 45 | MAXENT | 0.25 | 0.84 | 0.24 | 0.56 |
| *Z. pyrum* | 45 | RF | 0.51 | 0.95 | 0.50 | 0.73 |
| *Z. pyrum* | 46 | GBM | 0.51 | 0.87 | 0.51 | 0.61 |
| *Z. pyrum* | 46 | MAXENT | 0.24 | 0.84 | 0.24 | 0.51 |
| *Z. pyrum* | 46 | RF | 0.49 | 0.90 | 0.49 | 0.64 |
| *Z. pyrum* | 47 | GBM | 0.52 | 0.93 | 0.52 | 0.75 |
| *Z. pyrum* | 47 | MAXENT | 0.27 | 0.87 | 0.27 | 0.63 |
| *Z. pyrum* | 47 | RF | 0.53 | 0.97 | 0.53 | 0.88 |
| *Z. pyrum* | 48 | GBM | 0.47 | 0.87 | 0.47 | 0.56 |
| *Z. pyrum* | 48 | MAXENT | 0.24 | 0.89 | 0.24 | 0.67 |
| *Z. pyrum* | 48 | RF | 0.50 | 0.92 | 0.49 | 0.69 |
| *Z. pyrum* | 49 | GBM | 0.53 | 0.92 | 0.52 | 0.68 |
| *Z. pyrum* | 49 | MAXENT | 0.35 | 0.82 | 0.35 | 0.50 |
| *Z. pyrum* | 49 | RF | 0.51 | 0.96 | 0.51 | 0.75 |
| *Z. pyrum* | 50 | GBM | 0.50 | 0.91 | 0.50 | 0.64 |
| *Z. pyrum* | 50 | MAXENT | 0.27 | 0.85 | 0.26 | 0.56 |
| *Z. pyrum* | 50 | RF | 0.54 | 0.94 | 0.54 | 0.72 |
| *Z. pyrum* | 51 | GBM | 0.51 | 0.89 | 0.51 | 0.59 |
| *Z. pyrum* | 51 | MAXENT | 0.29 | 0.82 | 0.28 | 0.52 |
| *Z. pyrum* | 51 | RF | 0.49 | 0.94 | 0.49 | 0.69 |
| *Z. pyrum* | 52 | GBM | 0.47 | 0.89 | 0.48 | 0.65 |
| *Z. pyrum* | 52 | MAXENT | 0.25 | 0.86 | 0.25 | 0.60 |
| *Z. pyrum* | 52 | RF | 0.54 | 0.92 | 0.55 | 0.65 |
| *Z. pyrum* | 53 | GBM | 0.51 | 0.94 | 0.51 | 0.78 |
| *Z. pyrum* | 53 | MAXENT | 0.32 | 0.83 | 0.32 | 0.47 |
| *Z. pyrum* | 53 | RF | 0.51 | 0.97 | 0.51 | 0.84 |
| *Z. pyrum* | 54 | GBM | 0.44 | 0.89 | 0.44 | 0.65 |
| *Z. pyrum* | 54 | MAXENT | 0.24 | 0.90 | 0.24 | 0.68 |
| *Z. pyrum* | 54 | RF | 0.48 | 0.94 | 0.51 | 0.71 |
| *Z. pyrum* | 55 | GBM | 0.48 | 0.87 | 0.48 | 0.63 |
| *Z. pyrum* | 55 | MAXENT | 0.26 | 0.86 | 0.26 | 0.60 |
| *Z. pyrum* | 55 | RF | 0.51 | 0.92 | 0.51 | 0.64 |
| *Z. pyrum* | 56 | GBM | 0.52 | 0.91 | 0.52 | 0.58 |
| *Z. pyrum* | 56 | MAXENT | 0.26 | 0.83 | 0.26 | 0.52 |
| *Z. pyrum* | 56 | RF | 0.49 | 0.94 | 0.49 | 0.70 |
| *Z. pyrum* | 57 | GBM | 0.51 | 0.91 | 0.52 | 0.65 |
| *Z. pyrum* | 57 | MAXENT | 0.27 | 0.89 | 0.27 | 0.62 |
| *Z. pyrum* | 57 | RF | 0.53 | 0.96 | 0.53 | 0.69 |
| *Z. pyrum* | 58 | GBM | 0.50 | 0.92 | 0.50 | 0.72 |
| *Z. pyrum* | 58 | MAXENT | 0.27 | 0.86 | 0.27 | 0.57 |
| *Z. pyrum* | 58 | RF | 0.52 | 0.95 | 0.52 | 0.80 |
| *Z. pyrum* | 59 | GBM | 0.52 | 0.89 | 0.52 | 0.65 |
| *Z. pyrum* | 59 | MAXENT | 0.22 | 0.83 | 0.24 | 0.51 |
| *Z. pyrum* | 59 | RF | 0.51 | 0.94 | 0.54 | 0.71 |
| *Z. pyrum* | 60 | GBM | 0.48 | 0.87 | 0.49 | 0.63 |
| *Z. pyrum* | 60 | MAXENT | 0.25 | 0.84 | 0.27 | 0.60 |
| *Z. pyrum* | 60 | RF | 0.51 | 0.92 | 0.54 | 0.67 |
| *Z. pyrum* | 61 | GBM | 0.49 | 0.93 | 0.49 | 0.69 |
| *Z. pyrum* | 61 | MAXENT | 0.23 | 0.84 | 0.22 | 0.54 |
| *Z. pyrum* | 61 | RF | 0.53 | 0.96 | 0.54 | 0.81 |
| *Z. pyrum* | 62 | GBM | 0.53 | 0.85 | 0.53 | 0.55 |
| *Z. pyrum* | 62 | MAXENT | 0.31 | 0.83 | 0.30 | 0.50 |
| *Z. pyrum* | 62 | RF | 0.53 | 0.93 | 0.53 | 0.69 |
| *Z. pyrum* | 63 | GBM | 0.51 | 0.91 | 0.51 | 0.69 |
| *Z. pyrum* | 63 | MAXENT | 0.26 | 0.87 | 0.26 | 0.62 |
| *Z. pyrum* | 63 | RF | 0.52 | 0.95 | 0.53 | 0.73 |
| *Z. pyrum* | 64 | GBM | 0.49 | 0.91 | 0.51 | 0.55 |
| *Z. pyrum* | 64 | MAXENT | 0.26 | 0.87 | 0.26 | 0.58 |
| *Z. pyrum* | 64 | RF | 0.48 | 0.93 | 0.49 | 0.65 |
| *Z. pyrum* | 65 | GBM | 0.50 | 0.85 | 0.50 | 0.58 |
| *Z. pyrum* | 65 | MAXENT | 0.25 | 0.84 | 0.24 | 0.54 |
| *Z. pyrum* | 65 | RF | 0.52 | 0.90 | 0.49 | 0.67 |
| *Z. pyrum* | 66 | GBM | 0.52 | 0.93 | 0.53 | 0.69 |
| *Z. pyrum* | 66 | MAXENT | 0.22 | 0.85 | 0.24 | 0.56 |
| *Z. pyrum* | 66 | RF | 0.51 | 0.96 | 0.50 | 0.75 |
| *Z. pyrum* | 67 | GBM | 0.52 | 0.90 | 0.52 | 0.63 |
| *Z. pyrum* | 67 | MAXENT | 0.24 | 0.85 | 0.33 | 0.56 |
| *Z. pyrum* | 67 | RF | 0.51 | 0.95 | 0.51 | 0.66 |
| *Z. pyrum* | 68 | GBM | 0.48 | 0.92 | 0.48 | 0.69 |
| *Z. pyrum* | 68 | MAXENT | 0.24 | 0.85 | 0.23 | 0.63 |
| *Z. pyrum* | 68 | RF | 0.54 | 0.96 | 0.54 | 0.77 |
| *Z. pyrum* | 69 | GBM | 0.55 | 0.90 | 0.55 | 0.60 |
| *Z. pyrum* | 69 | MAXENT | 0.23 | 0.88 | 0.22 | 0.62 |
| *Z. pyrum* | 69 | RF | 0.52 | 0.92 | 0.52 | 0.69 |
| *Z. pyrum* | 70 | GBM | 0.53 | 0.87 | 0.53 | 0.61 |
| *Z. pyrum* | 70 | MAXENT | 0.27 | 0.86 | 0.27 | 0.59 |
| *Z. pyrum* | 70 | RF | 0.53 | 0.92 | 0.53 | 0.66 |
| *Z. pyrum* | 71 | GBM | 0.49 | 0.94 | 0.51 | 0.75 |
| *Z. pyrum* | 71 | MAXENT | 0.24 | 0.83 | 0.25 | 0.53 |
| *Z. pyrum* | 71 | RF | 0.51 | 0.98 | 0.51 | 0.83 |
| *Z. pyrum* | 72 | GBM | 0.53 | 0.86 | 0.53 | 0.46 |
| *Z. pyrum* | 72 | MAXENT | 0.25 | 0.88 | 0.25 | 0.62 |
| *Z. pyrum* | 72 | RF | 0.50 | 0.93 | 0.52 | 0.69 |
| *Z. pyrum* | 73 | GBM | 0.49 | 0.89 | 0.49 | 0.66 |
| *Z. pyrum* | 73 | MAXENT | 0.24 | 0.87 | 0.23 | 0.62 |
| *Z. pyrum* | 73 | RF | 0.55 | 0.92 | 0.55 | 0.73 |
| *Z. pyrum* | 74 | GBM | 0.51 | 0.93 | 0.51 | 0.74 |
| *Z. pyrum* | 74 | MAXENT | 0.26 | 0.87 | 0.27 | 0.59 |
| *Z. pyrum* | 74 | RF | 0.51 | 0.94 | 0.48 | 0.72 |
| *Z. pyrum* | 75 | GBM | 0.53 | 0.89 | 0.53 | 0.64 |
| *Z. pyrum* | 75 | MAXENT | 0.26 | 0.85 | 0.26 | 0.60 |
| *Z. pyrum* | 75 | RF | 0.55 | 0.91 | 0.55 | 0.69 |
| *Z. pyrum* | 76 | GBM | 0.50 | 0.87 | 0.50 | 0.59 |
| *Z. pyrum* | 76 | MAXENT | 0.29 | 0.81 | 0.29 | 0.46 |
| *Z. pyrum* | 76 | RF | 0.51 | 0.90 | 0.52 | 0.59 |
| *Z. pyrum* | 77 | GBM | 0.51 | 0.92 | 0.52 | 0.66 |
| *Z. pyrum* | 77 | MAXENT | 0.25 | 0.85 | 0.25 | 0.53 |
| *Z. pyrum* | 77 | RF | 0.51 | 0.95 | 0.52 | 0.75 |
| *Z. pyrum* | 78 | GBM | 0.51 | 0.89 | 0.51 | 0.72 |
| *Z. pyrum* | 78 | MAXENT | 0.27 | 0.85 | 0.26 | 0.60 |
| *Z. pyrum* | 78 | RF | 0.53 | 0.93 | 0.52 | 0.70 |
| *Z. pyrum* | 79 | GBM | 0.49 | 0.93 | 0.49 | 0.63 |
| *Z. pyrum* | 79 | MAXENT | 0.28 | 0.87 | 0.28 | 0.61 |
| *Z. pyrum* | 79 | RF | 0.49 | 0.96 | 0.49 | 0.72 |
| *Z. pyrum* | 80 | GBM | 0.46 | 0.88 | 0.47 | 0.56 |
| *Z. pyrum* | 80 | MAXENT | 0.32 | 0.87 | 0.30 | 0.64 |
| *Z. pyrum* | 80 | RF | 0.54 | 0.95 | 0.54 | 0.72 |
| *Z. pyrum* | 81 | GBM | 0.50 | 0.87 | 0.50 | 0.64 |
| *Z. pyrum* | 81 | MAXENT | 0.25 | 0.86 | 0.24 | 0.55 |
| *Z. pyrum* | 81 | RF | 0.50 | 0.91 | 0.49 | 0.66 |
| *Z. pyrum* | 82 | GBM | 0.50 | 0.95 | 0.50 | 0.79 |
| *Z. pyrum* | 82 | MAXENT | 0.26 | 0.85 | 0.26 | 0.61 |
| *Z. pyrum* | 82 | RF | 0.52 | 0.96 | 0.52 | 0.80 |
| *Z. pyrum* | 83 | GBM | 0.54 | 0.91 | 0.52 | 0.55 |
| *Z. pyrum* | 83 | MAXENT | 0.29 | 0.86 | 0.29 | 0.57 |
| *Z. pyrum* | 83 | RF | 0.49 | 0.96 | 0.51 | 0.80 |
| *Z. pyrum* | 84 | GBM | 0.48 | 0.88 | 0.50 | 0.59 |
| *Z. pyrum* | 84 | MAXENT | 0.25 | 0.85 | 0.24 | 0.60 |
| *Z. pyrum* | 84 | RF | 0.50 | 0.94 | 0.50 | 0.66 |
| *Z. pyrum* | 85 | GBM | 0.51 | 0.90 | 0.53 | 0.61 |
| *Z. pyrum* | 85 | MAXENT | 0.27 | 0.84 | 0.28 | 0.58 |
| *Z. pyrum* | 85 | RF | 0.52 | 0.95 | 0.53 | 0.73 |
| *Z. pyrum* | 86 | GBM | 0.52 | 0.94 | 0.51 | 0.67 |
| *Z. pyrum* | 86 | MAXENT | 0.26 | 0.88 | 0.26 | 0.60 |
| *Z. pyrum* | 86 | RF | 0.50 | 0.96 | 0.48 | 0.81 |
| *Z. pyrum* | 87 | GBM | 0.50 | 0.89 | 0.52 | 0.68 |
| *Z. pyrum* | 87 | MAXENT | 0.25 | 0.88 | 0.25 | 0.63 |
| *Z. pyrum* | 87 | RF | 0.50 | 0.94 | 0.52 | 0.74 |
| *Z. pyrum* | 88 | GBM | 0.51 | 0.90 | 0.51 | 0.66 |
| *Z. pyrum* | 88 | MAXENT | 0.23 | 0.83 | 0.22 | 0.58 |
| *Z. pyrum* | 88 | RF | 0.50 | 0.94 | 0.49 | 0.70 |
| *Z. pyrum* | 89 | GBM | 0.51 | 0.86 | 0.51 | 0.57 |
| *Z. pyrum* | 89 | MAXENT | 0.30 | 0.85 | 0.29 | 0.59 |
| *Z. pyrum* | 89 | RF | 0.50 | 0.91 | 0.53 | 0.63 |
| *Z. pyrum* | 90 | GBM | 0.50 | 0.90 | 0.49 | 0.70 |
| *Z. pyrum* | 90 | MAXENT | 0.25 | 0.84 | 0.24 | 0.57 |
| *Z. pyrum* | 90 | RF | 0.51 | 0.95 | 0.53 | 0.73 |
| *Z. pyrum* | 91 | GBM | 0.51 | 0.89 | 0.51 | 0.61 |
| *Z. pyrum* | 91 | MAXENT | 0.25 | 0.84 | 0.25 | 0.54 |
| *Z. pyrum* | 91 | RF | 0.48 | 0.93 | 0.49 | 0.72 |
| *Z. pyrum* | 92 | GBM | 0.51 | 0.87 | 0.52 | 0.59 |
| *Z. pyrum* | 92 | MAXENT | 0.25 | 0.89 | 0.24 | 0.62 |
| *Z. pyrum* | 92 | RF | 0.53 | 0.92 | 0.53 | 0.65 |
| *Z. pyrum* | 93 | GBM | 0.50 | 0.90 | 0.50 | 0.58 |
| *Z. pyrum* | 93 | MAXENT | 0.26 | 0.87 | 0.26 | 0.57 |
| *Z. pyrum* | 93 | RF | 0.54 | 0.94 | 0.54 | 0.66 |
| *Z. pyrum* | 94 | GBM | 0.50 | 0.92 | 0.50 | 0.69 |
| *Z. pyrum* | 94 | MAXENT | 0.34 | 0.85 | 0.34 | 0.56 |
| *Z. pyrum* | 94 | RF | 0.50 | 0.94 | 0.51 | 0.74 |
| *Z. pyrum* | 95 | GBM | 0.48 | 0.90 | 0.49 | 0.67 |
| *Z. pyrum* | 95 | MAXENT | 0.26 | 0.86 | 0.26 | 0.61 |
| *Z. pyrum* | 95 | RF | 0.55 | 0.94 | 0.55 | 0.73 |
| *Z. pyrum* | 96 | GBM | 0.50 | 0.93 | 0.50 | 0.73 |
| *Z. pyrum* | 96 | MAXENT | 0.32 | 0.87 | 0.32 | 0.66 |
| *Z. pyrum* | 96 | RF | 0.46 | 0.94 | 0.47 | 0.77 |
| *Z. pyrum* | 97 | GBM | 0.45 | 0.93 | 0.49 | 0.72 |
| *Z. pyrum* | 97 | MAXENT | 0.28 | 0.84 | 0.28 | 0.49 |
| *Z. pyrum* | 97 | RF | 0.52 | 0.96 | 0.52 | 0.83 |
| *Z. pyrum* | 98 | GBM | 0.43 | 0.88 | 0.48 | 0.64 |
| *Z. pyrum* | 98 | MAXENT | 0.26 | 0.87 | 0.26 | 0.58 |
| *Z. pyrum* | 98 | RF | 0.50 | 0.94 | 0.51 | 0.67 |
| *Z. pyrum* | 99 | GBM | 0.52 | 0.88 | 0.52 | 0.51 |
| *Z. pyrum* | 99 | MAXENT | 0.28 | 0.83 | 0.28 | 0.55 |
| *Z. pyrum* | 99 | RF | 0.50 | 0.92 | 0.51 | 0.68 |
| *Z. pyrum* | 100 | GBM | 0.49 | 0.89 | 0.49 | 0.64 |
| *Z. pyrum* | 100 | MAXENT | 0.25 | 0.85 | 0.24 | 0.57 |
| *Z. pyrum* | 100 | RF | 0.52 | 0.96 | 0.52 | 0.75 |
|  |  |  |  |  |  |  |

**Table S3** Shift distance (km) and direction (degrees) of potential distribution centroids between past (1850–2010), current (2010–2020) and future (2050–2060 and 2090–2100, under SSP2-4.5 and SSP5-8.5 scenarios) conditions.

| **Species** | **Transition** | **Distance (km)** | **Bearing (degrees)** | **Direction** |
| --- | --- | --- | --- | --- |
| *Luria lurida* | 1850-2010 -> 2010-2020 | 613.6204965 | 15.39733925 | NNE |
| *Luria lurida* | 2010-2020 -> 2050-2060 SSP2-4.5 | 1019.727375 | 185.162776 | S |
| *Luria lurida* | 2010-2020 -> 2050-2060 SSP5-8.5 | 790.7933719 | 193.4781431 | SSW |
| *Luria lurida* | 2010-2020 -> 2090-2100 SSP2-4.5 | 783.4268188 | 176.582368 | S |
| *Luria lurida* | 2010-2020 -> 2090-2100 SSP5-8.5 | 723.1532237 | 163.7790968 | SSE |
| *Naria spurca* | 1850-2010 -> 2010-2020 | 201.2268335 | 7.629830752 | N |
| *Naria spurca* | 2010-2020 -> 2050-2060 SSP2-4.5 | 738.0551957 | 90.51518839 | E |
| *Naria spurca* | 2010-2020 -> 2050-2060 SSP5-8.5 | 326.0109967 | 71.08564233 | ENE |
| *Naria spurca* | 2010-2020 -> 2090-2100 SSP2-4.5 | 494.1605508 | 68.03283861 | ENE |
| *Naria spurca* | 2010-2020 -> 2090-2100 SSP5-8.5 | 547.9701665 | 358.7629271 | N |
| *Zonaria pyrum* | 1850-2010 -> 2010-2020 | 85.56738613 | 160.7199806 | SSE |
| *Zonaria pyrum* | 2010-2020 -> 2050-2060 SSP2-4.5 | 149.6975839 | 166.6865778 | SSE |
| *Zonaria pyrum* | 2010-2020 -> 2050-2060 SSP5-8.5 | 553.1929746 | 280.6795305 | W |
| *Zonaria pyrum* | 2010-2020 -> 2090-2100 SSP2-4.5 | 618.0340431 | 282.6842112 | WNW |
| *Zonaria pyrum* | 2010-2020 -> 2090-2100 SSP5-8.5 | 907.7931614 | 280.7757193 | W |
| *Talisman scrobilator* | 1850-2010 -> 2010-2020 | 398.3917936 | 350.303452 | N |
| *Talisman scrobilator* | 2010-2020 -> 2050-2060 SSP2-4.5 | 1200.217233 | 238.5653171 | WSW |
| *Talisman scrobilator* | 2010-2020 -> 2050-2060 SSP5-8.5 | 1148.509186 | 237.413609 | WSW |
| *Talisman scrobilator* | 2010-2020 -> 2090-2100 SSP2-4.5 | 1083.358073 | 241.649729 | WSW |
| *Talisman scrobilator* | 2010-2020 -> 2090-2100 SSP5-8.5 | 899.1772378 | 248.3297552 | WSW |

## Figures


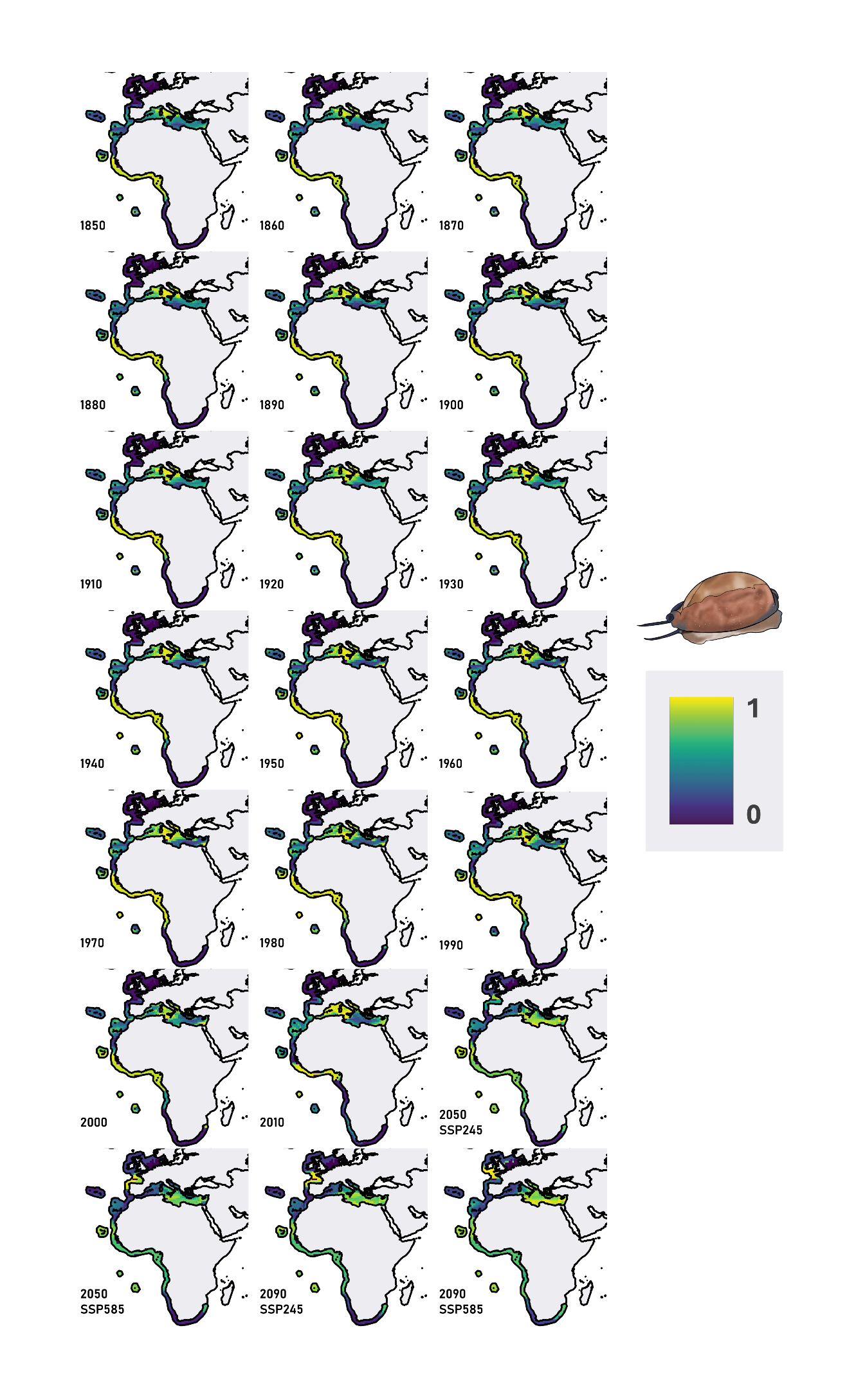


**Figure S1** Probability of occurrence of *L. lurida* from 1850 to 2020, and for 2050–2060 and 2090–2100 future scenarios (‘Middle of the Road’ SSP2-4.5 and ‘Fossil-fueled Development’ SSP5-8). [Species illustration by Noemi D’Ottavi]

*
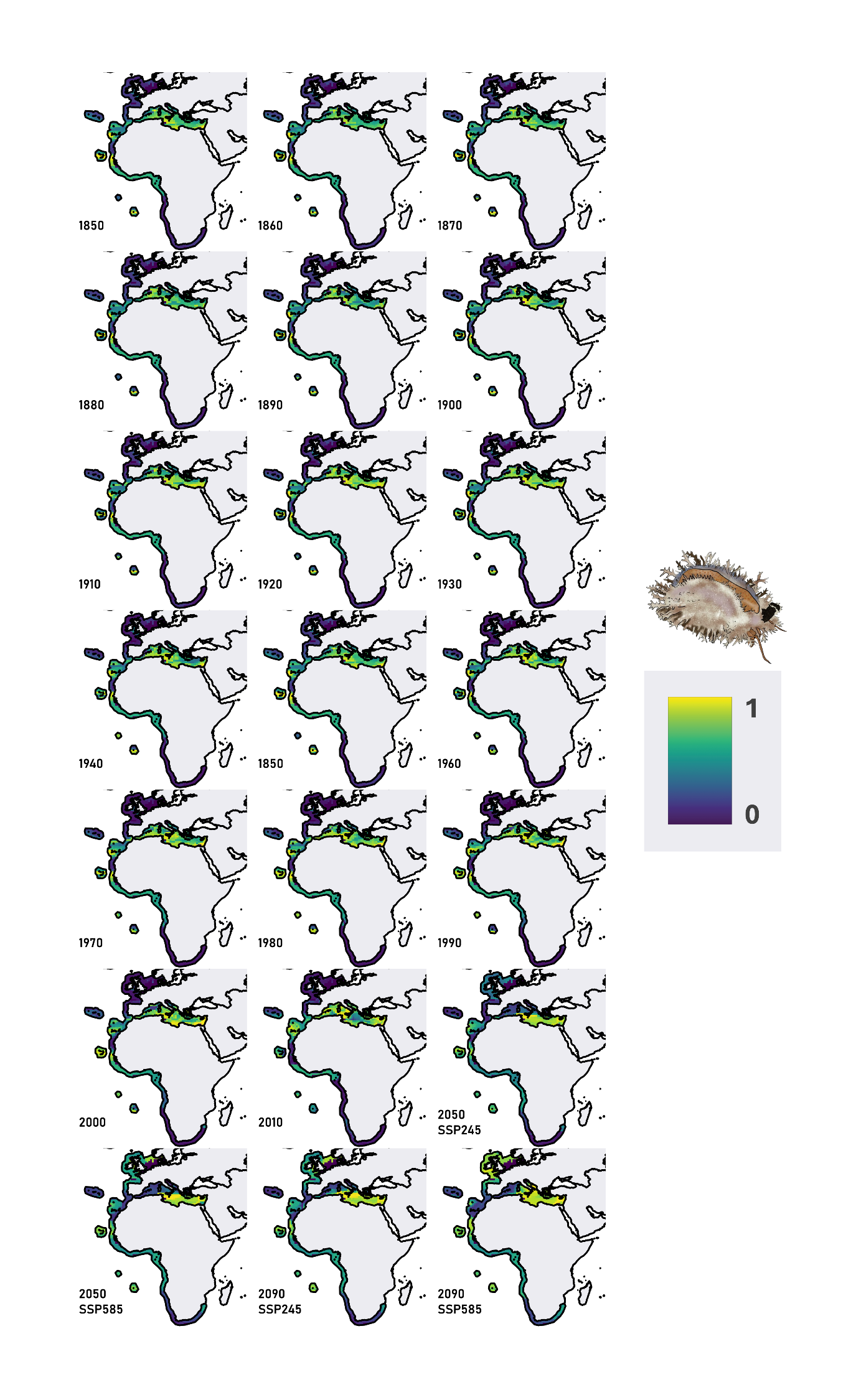
*

**Figure S2** Probability of occurrence of *N. spurca* from 1850 to 2020, and for 2050–2060 and 2090–2100 future scenarios (‘Middle of the Road’ SSP2-4.5 and ‘Fossil-fueled Development’ SSP5-8). [Species illustration by Noemi D’Ottavi]

*
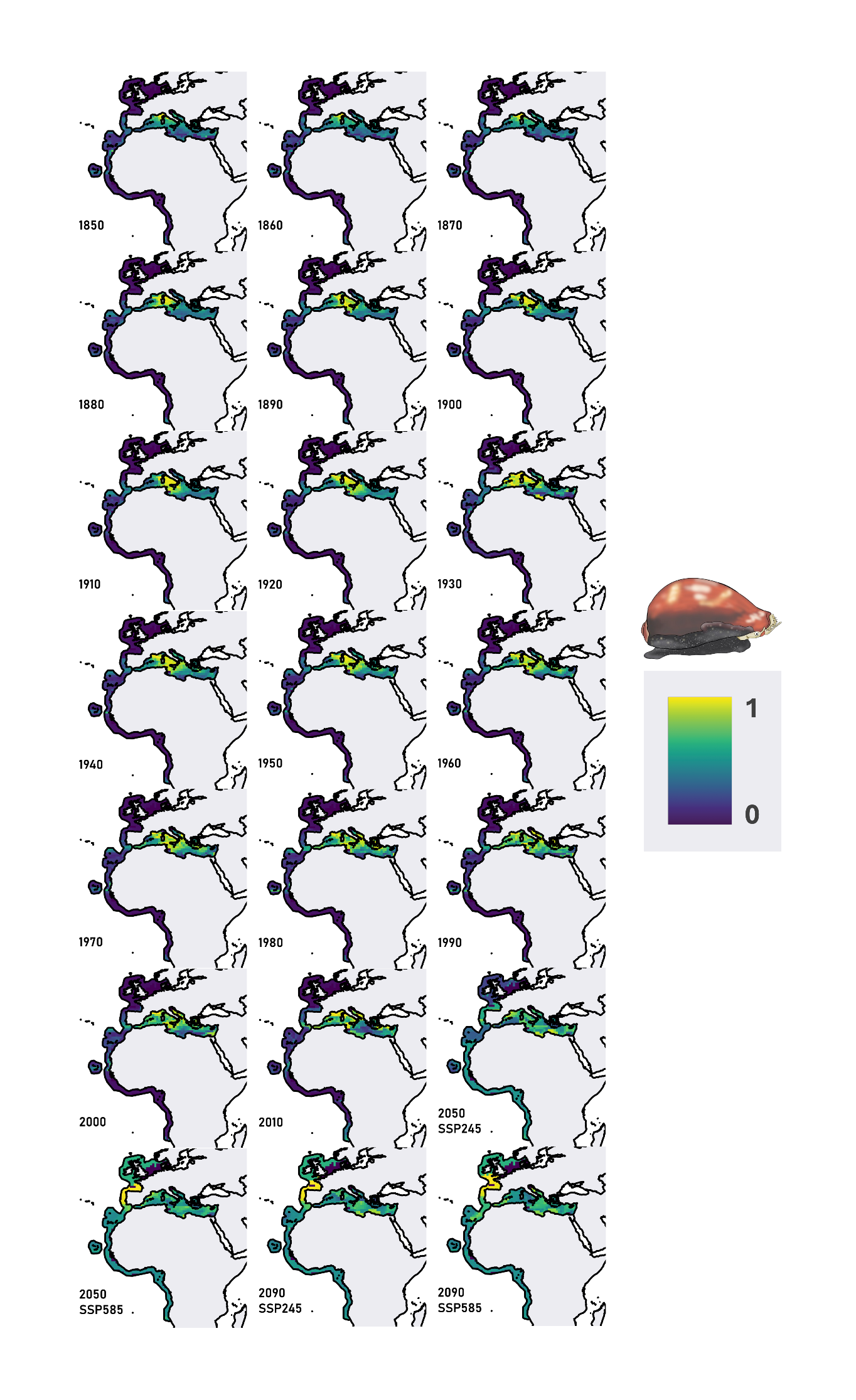
*

**Figure S3** Probability of occurrence of *Z. pyrum* from 1850 to 2020, and for 2050–2060 and 2090–2100 future scenarios (‘Middle of the Road’ SSP2-4.5 and ‘Fossil-fueled Development’ SSP5-8). [Species illustration by Noemi D’Ottavi]

*
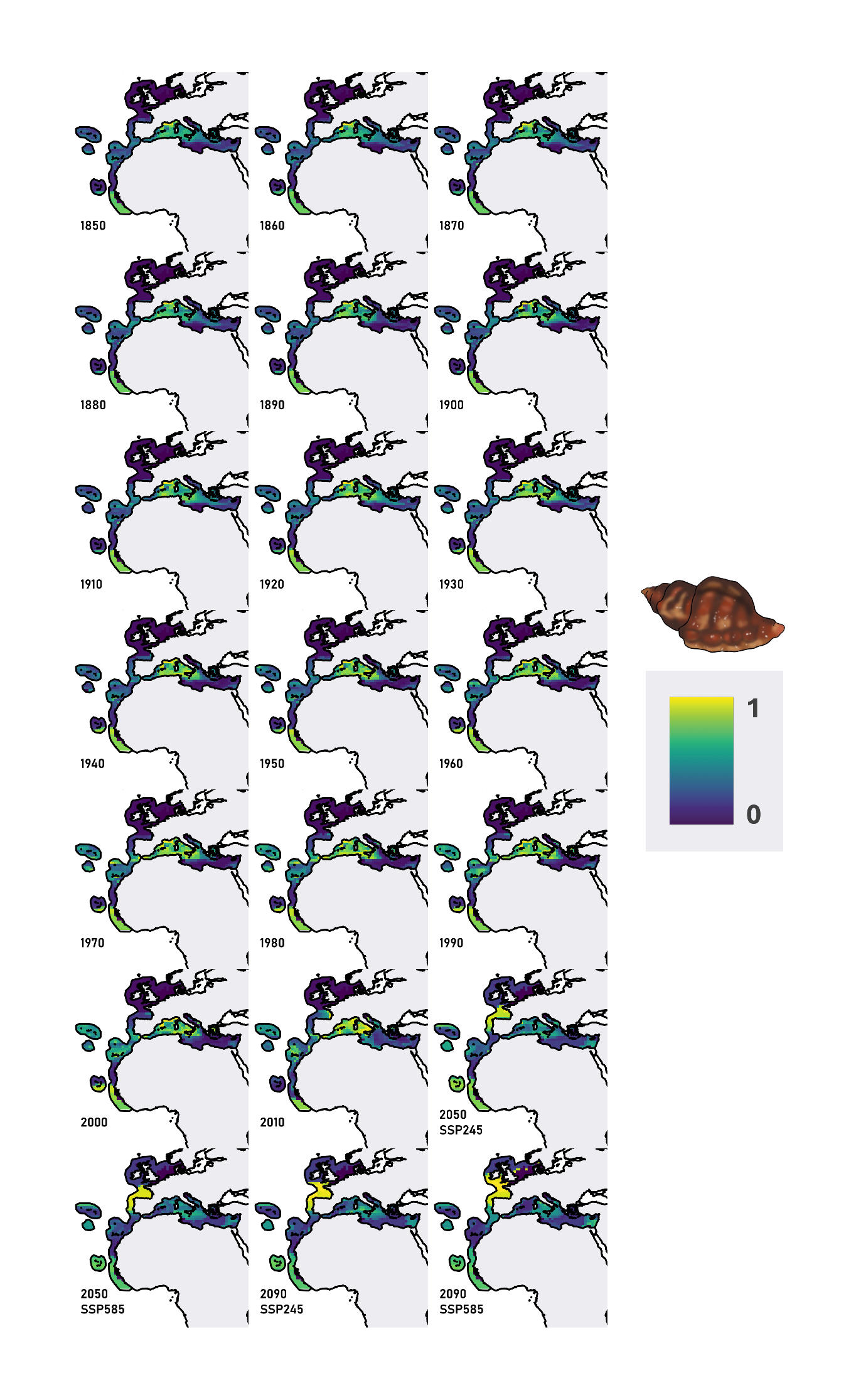
*

**Figure S4** Probability of occurrence of *T. scrobilator* from 1850 to 2020, and for 2050–2060 and 2090–2100 future scenarios (‘Middle of the Road’ SSP2-4.5 and ‘Fossil-fueled Development’ SSP5-8). [Species illustration by Noemi D’Ottavi]

*
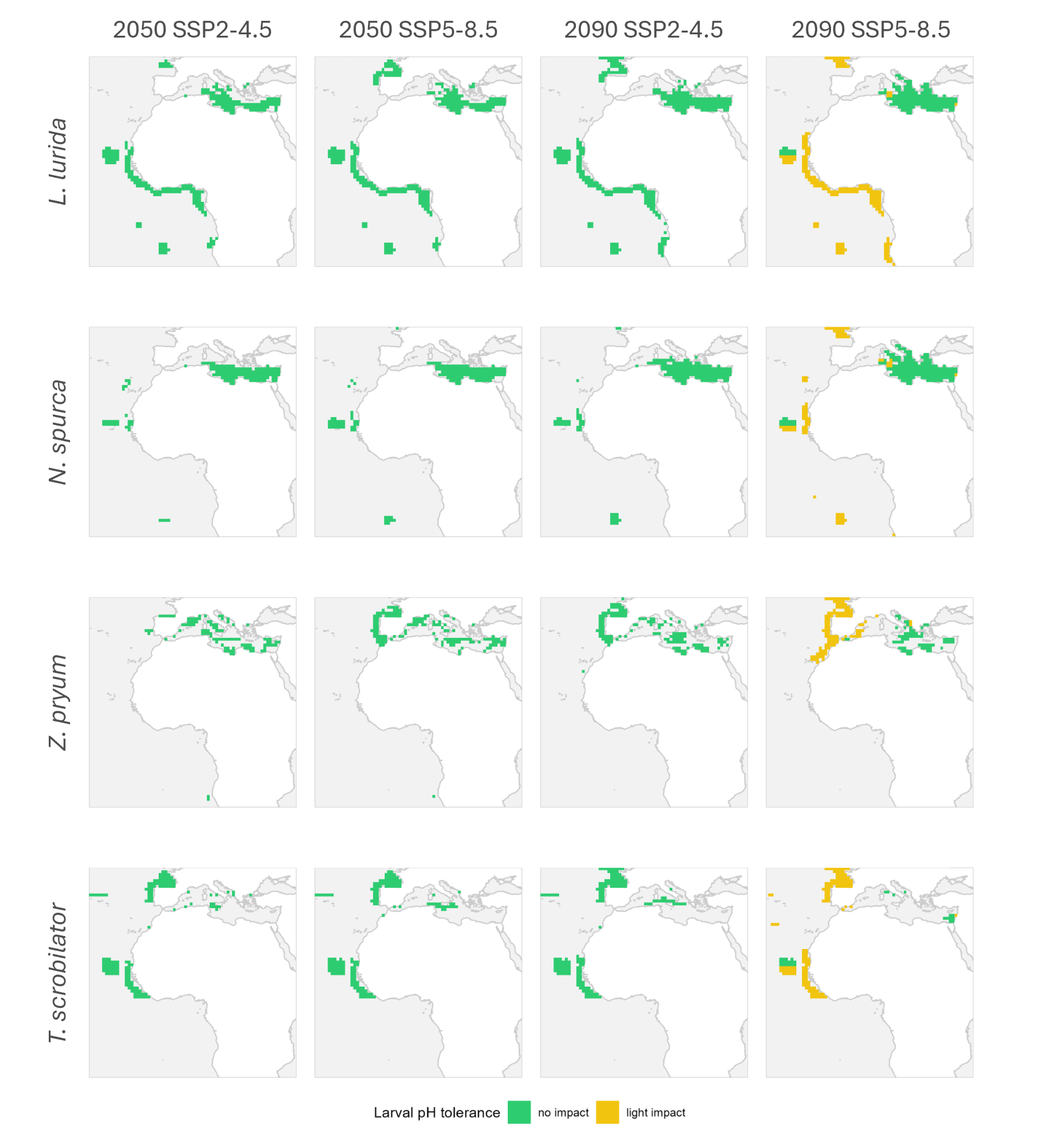
*

**Figure S5** Spatial mismatches between adult habitat suitability (the entire coloured area) and larval viability (no impact: pH ≥ 7.70, green; light impact: 7.55 ≤ pH < 7.70, yellow), incorporating larval tolerance to ocean acidification. No areas with medium impact (7.44 ≤ pH < 7.55) or severe impact (pH < 7.44) were found.
